# A mutualistic bacterium rescues a green alga from an antagonist

**DOI:** 10.1073/pnas.2401632121

**Published:** 2024-04-03

**Authors:** David Carrasco Flores, Vivien Hotter, Trang Vuong, Yu Hou, Yuko Bando, Kirstin Scherlach, Bertille Burgunter-Delamare, Ron Hermenau, Anna J. Komor, Prasad Aiyar, Magdalena Rose, Severin Sasso, Hans-Dieter Arndt, Christian Hertweck, Maria Mittag

**Affiliations:** ^a^Matthias Schleiden Institute of Genetics, Bioinformatics and Molecular Botany, General Botany, Friedrich Schiller University Jena, Jena 07743, Germany; ^b^Institute for Organic Chemistry and Macromolecular Chemistry, Organic Chemistry, Friedrich Schiller University Jena, Jena 07743, Germany; ^c^Department of Biomolecular Chemistry, Leibniz Institute for Natural Product Research and Infection Biology (Hans Knöll Institute), Jena 07745, Germany; ^d^Institute of Biology, Plant Physiology, Leipzig University, Leipzig 04103, Germany; ^e^Cluster of Excellence Balance of the Microverse, Friedrich Schiller University Jena, Jena 07743, Germany; ^f^Faculty of Biological Sciences, Friedrich Schiller University Jena, Jena 07743, Germany

**Keywords:** *Chlamydomonas reinhardtii*, cyclic lipopeptide, microbial interactions, *Mycetocola*, *Pseudomonas*

## Abstract

Photosynthetic protists, known as microalgae, are key contributors to primary production on Earth. Since early in evolution, they coexist with bacteria in nature, and their mode of interaction shapes ecosystems. We have recently shown that the bacterium *Pseudomonas protegens* acts algicidal on the microalga *Chlamydomonas reinhardtii.* It secretes a cyclic lipopeptide and a polyyne that deflagellate, blind, and lyse the algae [P. Aiyar *et al.*, *Nat. Commun.* **8**, 1756 (2017) and V. Hotter *et al.*, *Proc. Natl. Acad. Sci. U.S.A.* **118**, e2107695118 (2021)]. Here, we report about the bacterium *Mycetocola lacteus,* which establishes a mutualistic relationship with *C. reinhardtii* and acts as a helper. While *M. lacteus* enhances algal growth, it receives methionine as needed organic sulfur and the vitamins B_1_, B_3_, and B_5_ from the algae. In tripartite cultures with the alga and the antagonistic bacterium *P. protegens*, *M. lacteus* aids the algae in surviving the bacterial attack. By combining synthetic natural product chemistry with high-resolution mass spectrometry and an algal Ca^2+^ reporter line, we found that *M. lacteus* rescues the alga from the antagonistic bacterium by cleaving the ester bond of the cyclic lipopeptide involved. The resulting linearized seco acid does not trigger a cytosolic Ca^2+^ homeostasis imbalance that leads to algal deflagellation. Thus, the algae remain motile, can swim away from the antagonistic bacteria and survive the attack. All three involved genera cooccur in nature. Remarkably, related species of *Pseudomonas* and *Mycetocola* also act antagonistically against *C. reinhardtii* or as helper bacteria in tripartite cultures.

## Significance

Although they are of great ecological importance for global CO_2_ fixation and food webs, knowledge about microalgae and their molecular interplay with bacteria is limited. Here, we show how a *Chlamydomonas* microalga supports the growth of a mutualistic *Mycetocola* sp. that, in turn, protects the alga from an antagonistic *Pseudomonas* bacterium. We elucidate how two *Mycetocola* species inactivate a chemical mediator that is secreted by an antagonistic bacterium through cleavage of an ester bond. This change prevents the toxin-triggered increase of cytosolic Ca^2+^ levels in the alga and, thus, immobilization. The results provide an important step toward understanding multipartner bacterial-algal interactions. They highlight some common mechanistic principles used in algal-bacterial interactions of *Chlamydomonas*, *Pseudomonas* and *Mycetocola,* and other interkingdom relations.

Microalgae contribute significantly to primary production and are at the basis of food webs (1). In nature, they interact with a broad variety of microorganisms that influence their fitness (2). Despite the importance of these interactions for life on Earth, our knowledge about microbial interactions, including those involving microalgae, is still limited. Multiple studies have established the pivotal role of bacterial and fungal partners in algal growth promotion (3–6). For example, in both aquatic and soil habitats, bacteria can supply microalgae with vitamins B_1_ and/or B_12_ in exchange for photosynthate or other vitamins (7, 8). But bacteria can also inhibit algal growth by secreting algicidal compounds (9). Often, microbial interactions are studied exclusively in bipartite cultures. In nature, however, there are more than two microbial species present and communicate among each other. Hence, additional interaction partners must be considered to understand interactions in complex microbial consortia. The introduction of a third microbial member can change the type of interaction. For instance, the algicidal effect of the soil bacterium *Streptomyces iranensis* on the green microalga *Chlamydomonas reinhardtii* can be neutralized by introducing the fungus *Aspergillus nidulans*, which shields the alga from the bacterial attack (10). In other cases, so-called helper bacteria prevent antagonistic interactions. Fungi that are sensitive to bacterial phenazines can be rescued by a second bacterium, which sequesters these harmful compounds, acting as a sponge (11). Such helper bacteria can also destroy bacterial toxic cyclic lipopeptides (CLiPs) like tolaasin I by enzymatic cleavage (12), resulting in the inactivation of the chemical mediator (13).

In previous studies, we used the biciliate soil-dwelling *C. reinhardtii* as a model organism (14–16) for studying microbial interactions and analyzed its interactions with different soil bacteria, including the genus *Pseudomonas*. These Gammaproteobacteria are present ubiquitously in aquatic and terrestrial environments (17, 18) and were also found in synthetic communities of the phycosphere of *C. reinhardtii* (19). We found that the soil bacterium *Pseudomonas protegens* Pf-5 (abbreviated as *P. protegens* from here on) has a strong inhibitory effect on algal growth (20) with several secondary metabolites of *P. protegens* being involved (20–22). Some cause immobilization of *C. reinhardtii* by removing its cilia, a process known as deflagellation (23). The CLiP orfamide A is the primary and highly potent toxin causing Ca^2+^ homeostasis disruption and thus immobilization with an IC_50_ value of 4.1 µM (22). It also modifies algal morphology (20). Orfamide A causes a quick reaction (1-min range) and uses at least four different algal Ca^2+^ channels of the transient receptor potential type (24). Another compound, the polyyne protegencin, targets the alga’s eyespot, blinds and lyses the algal cells within several hours (21).

In this study, we introduced a third interaction partner, *Mycetocola lacteus*, a Gram-positive bacterium from the phylum Actinomycetota. Like the Gram-negative proteobacteria from the phylum Pseudomonadota, *Mycetocola* are widely present in nature. They have been found not only on mushrooms (12, 25, 26) but also in bacterial communities of soil, in the desert, or in the deep sea (27–29). *M. lacteus* was previously shown to be involved in fungal-bacterial interactions. It hydrolyzes the CLiP tolaasin I from *Pseudomonas tolaasii* involved in harming the mushroom *Agaricus bisporus* (12). Here, we show that *M. lacteus* acts as a helper bacterium and rescues the algal cells. It inactivates the CLiP orfamide A by cleavage, resulting in biologically inactive forms. Moreover, a related bacterium, *Mycetocola tolaasinivorans*, is able to cleave this CLiP. Remarkably, *M. lacteus* benefits from the presence of the algae. The algae secrete vitamins B_1_, B_3_, and B_5_ as well as organic sulfur in form of methionine, which are metabolites essential for the growth of *M. lacteus* or its sustained growth in case of B_1_. Thus, *C. reinhardtii* enables the growth of *M. lacteus*. In return, *M. lacteus* enhances algal growth. These findings offer valuable insights into the dynamic interplay between a mutualistic and an antagonistic bacterium, influencing the fitness of the algae. Furthermore, they reveal a shared principle for rendering chemical mediators inactive in tripartite cultures, encompassing fungal-bacterial as well as algal-bacterial interactions.

## Results

### The Bacterium *M. lacteus* and the Green Alga *C. reinhardtii* Benefit from Each Other in Coculture.

**Fig. 1.**
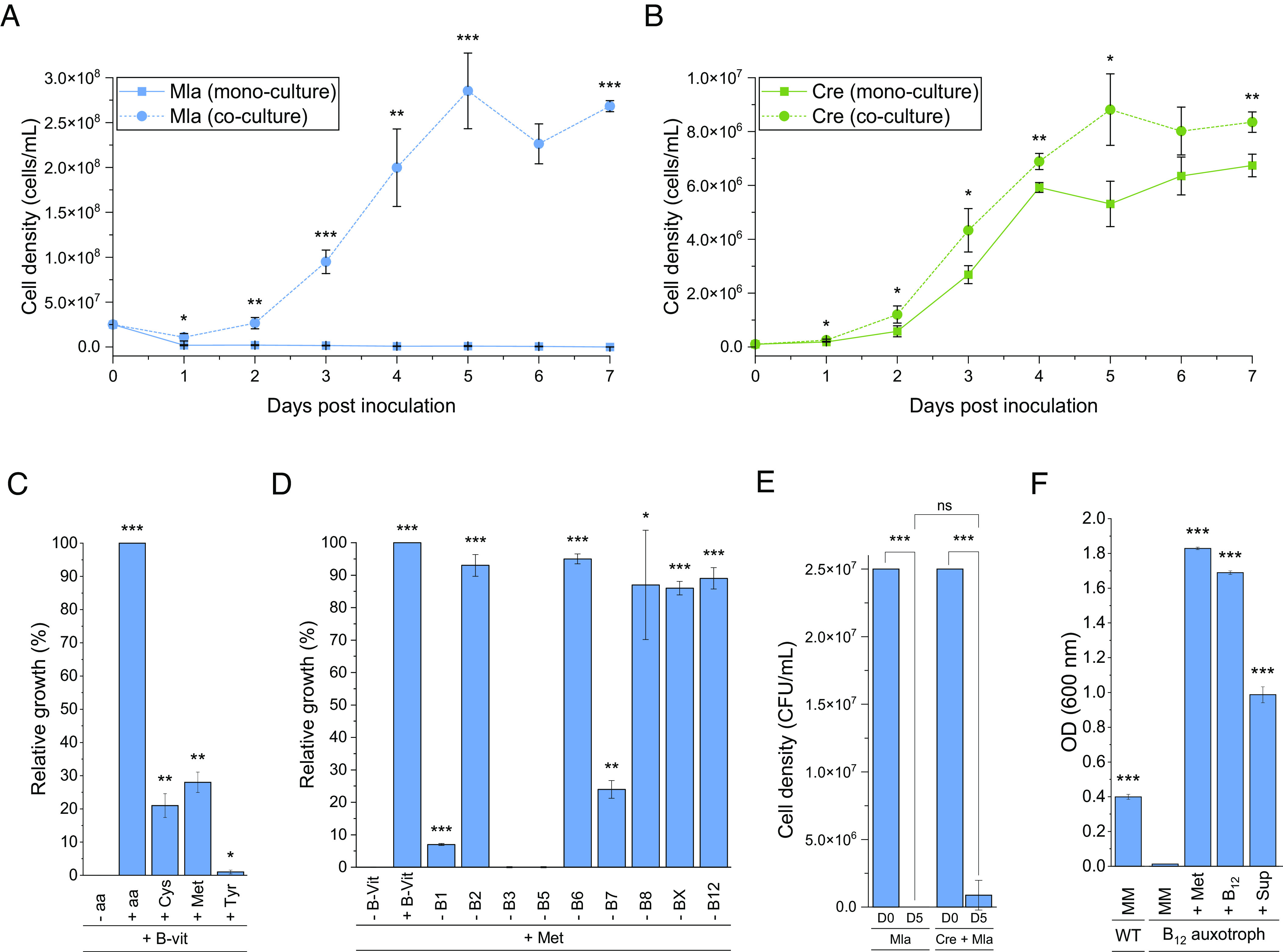
While *M. lacteus* needs methionine and some B vitamins from *C. reinhardtii*, it enhances algal growth. (*A*) Axenic *M. lacteus* cannot grow in TAP medium with 0.2% (w/v) glucose, but cocultivation with *C. reinhardtii* enables bacterial growth. The two organisms were cocultivated at a ratio of 1:250 (algae:bacteria) and grown for 7 d in TAP medium supplemented with 0.2% (w/v) glucose. Bacterial cell densities were calculated by serially diluting and plating on LB medium daily. Colonies were counted after 2 d of growth, and cell densities were determined considering the dilution factor. Mla: *M. lacteus*. Asterisks indicate significant differences between mono- and cocultures (*A* and *B*) and between negative controls and supplemented cultures (*C*–*E*) as calculated by Student’s *t* test (*P ≤ 0.05; **P ≤ 0.01; and ***P ≤ 0.001). The error bars indicate SDs with n = 3 biological replicates per time point and culture. (*B*) *M. lacteus* significantly enhances the growth of *C. reinhardtii* in TAP medium with 0.2% (w/v) glucose. Algal cells were counted daily using a Thoma cell counting chamber. Cre: *C. reinhardtii*; see legend of (*A*) for further details. (*C*) *M. lacteus* needs cysteine or methionine for its growth. TAP medium with 0.2% (w/v) glucose and a mixture of the nine B-vitamins was used as medium. To investigate the bacterium’s need for amino acids, each *M. lacteus* culture was supplemented with either no amino acids (−aa), a mixture of the 20 amino acids (+aa), or one amino acid at a time. For details, see *SI Appendix, Methods*. The experiments represent the average of three independent biological replicates with the error bars denoting the SD. (*D*) *M. lacteus* needs certain B vitamins for its growth. TAP medium supplemented with 0.2% (w/v) glucose and methionine was used as medium. To test the bacterial B vitamin requirements, *M. lacteus* cultures were grown with either no B vitamins (−B-Vit), the mixture of the nine B vitamins (+B-Vit) or eight out of the nine B vitamins (one different vitamin always absent). See legend (*C*) for further details. (*E*) *C. reinhardtii* does not provide glucose for *M. lacteus*. TP medium without acetate and glucose was used as growth medium for a monoculture of *M. lacteus* and a coculture together with *C. reinhardtii*. D0, inoculation; D5, 5 d of growth. (*F*) *C. reinhardtii* spent medium provides methionine and thus can complement an *E. coli* auxotroph mutant to produce B_12_. Comparison of the growth of *E. coli* K12 DH5α wild type (WT) in M9 MM with an *E. coli* auxotroph mutant for the production of vitamin B_12_ in M9 MM supplemented with either nothing, vitamin B_12_, methionine (Met), or *C. reinhardtii* spent medium (supernatant, Sup); see *SI Appendix, Methods* for details.

Before starting with tripartite cultures between *M. lacteus*, the algicidal bacterium *P. protegens* and *C. reinhardtii*, we investigated the interaction between the alga and *M. lacteus* in bipartite cultures. At first, we studied the growth of *M. lacteus* alone in a medium that is used for algal growth. The algal culture medium TAP (Tris-Acetate-Phosphate) (30) was supplemented with 0.2% (w/v) glucose, because several members of the genus *Mycetocola* including *M. lacteus* cannot use acetate as a carbon source (31). Although the glucose-complemented medium did not support the growth of *M. lacteus* (Fig. 1*A*), the bacteria grew when cocultured with the algal cells (Fig. 1*A*). In the coculture, the bacterium reached cell densities of about 10 times higher than its initial inoculation density within 4 to 5 d (Fig. 1*A*). *C. reinhardtii* itself grew well in the above-mentioned medium, but its growth was still significantly enhanced in the presence of *M. lacteus* (Fig. 1*B*). Thus, both *M. lacteus* and *C. reinhardtii* establish a mutualistic partnership and benefit from each other in coculture.

### *C. reinhardtii* Provides Vitamins B_1_, B_3_, and B_5_ for *M. lacteus* as Well as Organic Sulfur in Form of Methionine.

To understand how *C. reinhardtii* promotes the growth of *M. lacteus* in the selected medium, we investigated whether the algal cells could provide essential compounds for the bacterium’s growth such as amino acids or B vitamins. At first, TAP medium with 0.2% (w/v) glucose was supplemented with a mixture of nine B vitamins (B_1,_ B_2_, B_3_, B_5_, B_6_, B_7_, B_8_, B_10_, and B_12_) and the 20 proteinogenic amino acids. Indeed, *M. lacteus* was able to grow under these conditions, while it did not grow in the absence of either the amino acids or the B vitamin mix (Fig. 1 *C* and *D*).

To determine which amino acids are required for bacterial growth, *M. lacteus* was grown in the chosen medium supplemented with the nine B vitamins but only one of the 20 proteinogenic amino acids at a time. Out of the 20 amino acids, both methionine and cysteine supported the growth of *M. lacteus* (Fig. 1*C* and *SI Appendix*, Fig. S1*A*). In addition, *M. lacteus* grew marginally in the presence of tyrosine. All other individually tested proteinogenic amino acids could not sustain the growth of *M. lacteus* under the tested conditions (*SI Appendix*, Fig. S1*A*).

We also tested whether specific B vitamins are needed. For that, *M. lacteus* was grown in TAP medium supplemented with glucose and methionine, and eight of the nine B vitamins were omitted one at a time. Our data show that *M. lacteus* is auxotrophic for vitamins B_3_ and B_5_; it needs both vitamins to survive. In addition, the lack of vitamins B_1_ and B_7_ impaired the growth significantly, with growth rates 70% (B_7_) to 90% (B_1_) lower than compared to the control (Fig. 1*D* and *SI Appendix*, Fig. S1*B*).

Next, we examined whether *C. reinhardtii* can support the growth of *M. lacteus* by secreting glucose or another carbon source that can be used by *M. lacteus* (31). For this purpose, we grew *M. lacteus* in TP medium lacking any carbon source (*SI Appendix*, *Methods*) either in mono- (negative control) or in coculture with *C. reinhardtii*. As expected, *M. lacteus* does not grow in TP medium alone. All cells died after 5 d of incubation (Fig. 1*E*). Moreover, no significant growth of *M. lacteus* was found in a coculture with *C. reinhardtii* (Fig. 1*E*). Thus, the algal cells do not secrete glucose or another carbon source usable by *M. lacteus* in a sufficient amount to sustain bacterial growth.

It is known that *Chlamydomonas* species can secrete some B vitamins (*Discussion*). However, it was not evident whether they could release methionine or cysteine. We tested whether *C. reinhardtii* could secrete any of the two amino acids to the medium by analyzing the extracellular compounds released. Using high-performance liquid chromatography, we found that *C. reinhardtii* can secrete a compound with the same retention time as the derivatized methionine to the medium, but that seems not to be the case for cysteine (*SI Appendix*, Fig. S2).

To verify whether the compound secreted from the algal cells was indeed methionine, we used an auxotrophic *Escherichia coli* mutant that can only grow in the presence of vitamin B_12_, which is needed for methionine synthesis, or methionine itself. The mutant’s growth was supported by addition of i) methionine, ii) vitamin B_12_ as well as iii) the supernatant of a *C. reinhardtii* culture added to the bacterial minimal medium (MM) (Fig. 1*F*). As *C. reinhardtii* cannot produce vitamin B_12_ (32), the bacterial growth rescue must be due to algal secretion of methionine. Furthermore, we verified whether a cysteine auxotrophic *E. coli* mutant can be complemented with the supernatant of a *C. reinhardtii* culture (*SI Appendix*, Fig. S2*C*). This is not the case, corroborating that cysteine is not secreted by *C. reinhardtii*, in contrast to methionine.

Based on the observation that *M. lacteus* could not use the inorganic sulfate present in a TAP medium, and the fact that it needs methionine as a sulfur-containing amino acid, we checked whether the bacterium may be auxotrophic for this amino acid. An alternative is that it cannot reduce the sulfate from the TAP medium to organic sulfur compounds needed for cysteine and methionine biosynthesis. For this purpose, we analyzed whether *M. lacteus* can use organic sulfur compounds for its growth. *M. lacteus* was cultured in a TAP medium supplemented with glucose, vitamins B_1_, B_3_, B_5,_ and B_7_, and one organic sulfur source at a time, leaving away all proteinogenic amino acids. Our findings show that *M. lacteus* grows in the presence of β-mercaptoethanol, dithiothreitol, glutathione, lipoic acid, and dimethyl sulfoxide without the need of either methionine or B_12_ (*SI Appendix*, Fig. S3). Therefore, *M. lacteus* has no auxotrophy for methionine but is apparently unable to transform sulfate into organic sulfur compounds. The secreted algal methionine rescues bacterial growth by making reduced organic sulfur available.

Subsequently, we quantified the amount of methionine released by *C. reinhardtii* (*SI Appendix*, Fig. S4 *A*–*C*), along with assessing the nature and amount of B vitamins (*SI Appendix*, Fig. S4 *D*–*F*). For this purpose, the supernatants of algal monocultures were analyzed. *C. reinhardtii* releases about 12 to 18 ng methionine as well as 1 to 3 ng vitamins B_1_, B_3,_ and B_5_ per million algal cells within 5 d (*SI Appendix*, Table S1 and *Methods*). Vitamin B_7_ was not detected in the supernatant. The amounts of all secreted biomolecules were decreased in a supernatant of an algal coculture with *M. lacteus* compared to the algal monoculture (*SI Appendix*, Table S1), suggesting the uptake of these compounds by *M. lacteus*.

### Confronting Friend and Antagonist: Helper Bacteria Facilitate the Recovery of *C. reinhardtii* after an Attack by Antagonistic Bacteria.

**Fig. 2.**
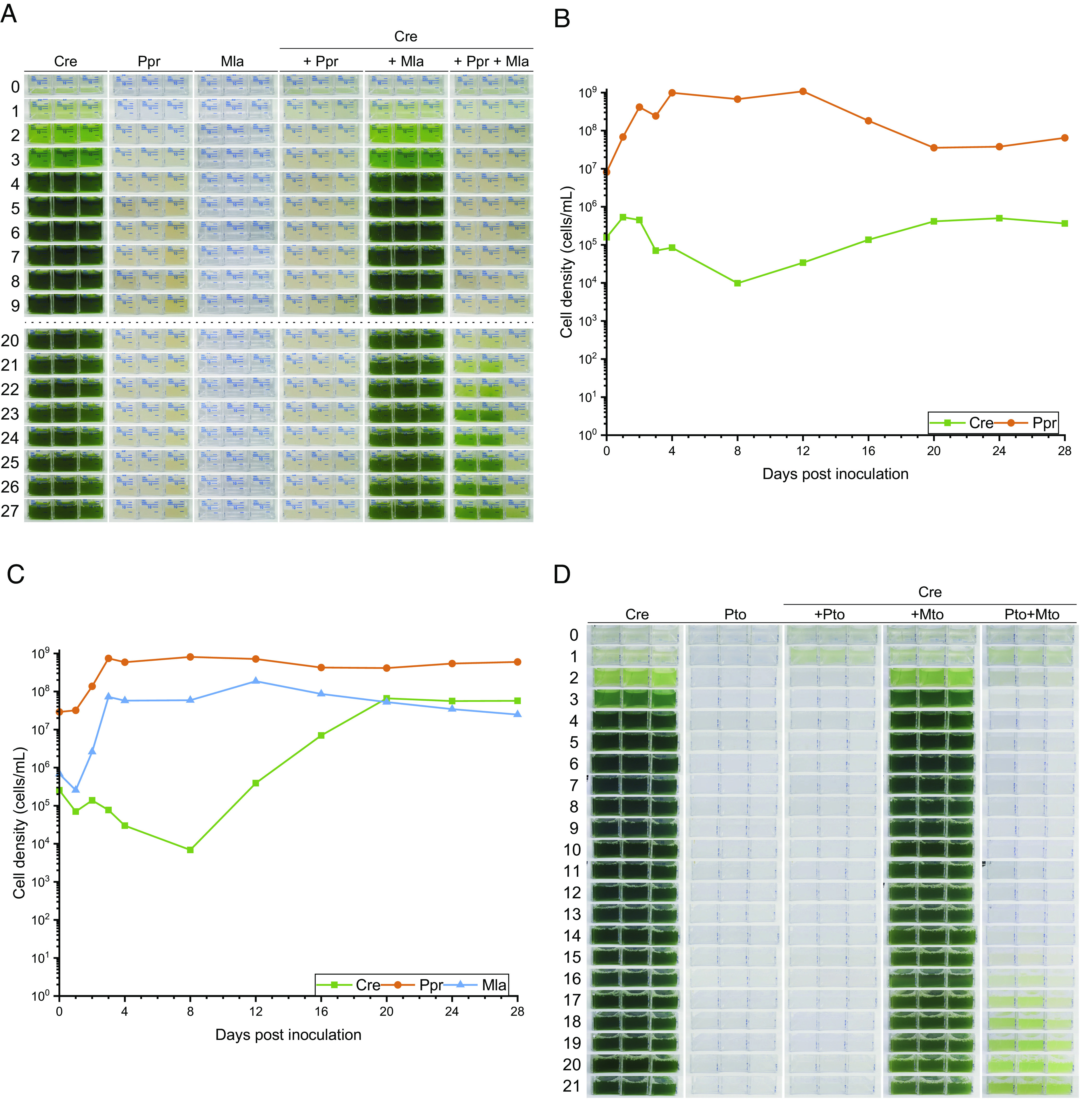
The helper bacteria *M. lacteus* and *M. tolaasinivorans*, respectively, aid *C. reinhardtii* in recovering from the antagonistic bacteria *P. protegens* and *P. tolaasii*. (*A*): Algal and bacterial monocultures as well as bi- and tripartite cultures. The organisms were cocultivated in TAP medium containing 0.1 mM phosphate supplemented with 0.2% (w/v) glucose at a ratio of 1:250:250 (Cre:Ppr:Mla). Equally treated cultures of *C. reinhardtii* (Cre), *P. protegens* (Ppr), and *M. lacteus* (Mla) served as references for the growth of the axenic cultures. Pictures from days 10 until 19 that equal day 9 can be seen in *SI Appendix*, Fig. S6 along with further replicates. (*B*): Algal and bacterial cell densities in a bipartite culture of *C. reinhardtii* and *P. protegens* over 28 d. For cultivation, see legend of (*A*). Cells from 10 mL cell suspension were harvested on days 0 to 4 postinoculation and then every fourth day. Genomic DNA was extracted and used for qPCR to determine cell densities [see (*A*) and *SI Appendix*, *Methods* for details as well as *SI Appendix*, Fig. S7]. (*C*): Algal and bacterial cell densities in a tripartite culture of *C. reinhardtii*, *P. protegens,* and *M. lacteus* over the course of 28 d. See (*B*) for further details. (*D*) Algal and bacterial monocultures as well as bi- and tripartite cultures. The organisms were cocultivated as described in (*A*), but *P. tolaasii* (Pto) and *M. tolaasinivorans* (Mto) were used as bacteria. Two further independent biological replicates are shown in *SI Appendix*, Fig. S8.

To find out whether *M. lacteus* may influence the antagonistic effect of the bacterium *P. protegens* on *C. reinhardtii*, we investigated tripartite cultures of the two different bacteria together with the algae. For the growth of the cultures, we used TAP medium supplemented with 0.2% glucose with low phosphate concentration (*SI Appendix, Methods*), observed to generate robust bacterial virulence in a related *Pseudomonas* species (33). Algal cell growth is halted upon the first day in coculture with *P. protegens* under these conditions (*SI Appendix*, Fig. S5*A*). Under low phosphate concentration, the growth of *M. lacteus* is only enabled in coculture with *C. reinhardtii* (*SI Appendix*, Fig. S5*B*) as seen before (Fig. 1*A*). The growth promotion of the algal cells by *M. lacteus* is reduced here (*SI Appendix*, Fig. S5*A*). Under low phosphate conditions, *P. protegens* grows to similar cell counts in mono- and cocultures with the algal cells (*SI Appendix*, Fig. S5 *C* and *D*). Under the conditions used, the algal cells did not recover in cocultures of *P. protegens* and *C. reinhardtii* within a 25 to 28-d period (Fig. 2*A* and *SI Appendix*, Figs. S6 and S7) in nearly all cases. In tripartite cultures along with *M. lacteus*, the algae recovered on average after about 20 d (Fig. 2*A*). Earlier or later recovery was also observed in other independent biological replicates (*SI Appendix*, Figs. S6 and S7*A*). Albeit the recovery could vary by some days, it should be noted that the bipartite culture of *P. protegens* and *C. reinhardtii* always recovered more slowly (in most cases never) than the tripartite cultures (Fig. 2*A* and *SI Appendix*, Figs. S6 and S7). Thus, *M. lacteus* not only promotes algal growth in a bipartite culture, where the algal cells grow well over the time of the experiment (Figs. 1*B* and 2*A*, Cre + Mla), but also functions as a helper bacterium promoting algal recovery in the tripartite culture (Fig. 2*A*, Cre + Ppr + Mla).

To get a deeper insight into the beneficial effect of *M. lacteus* on *C. reinhardtii* in the tripartite culture, we analyzed the cell number of each microorganism and compared it to the bipartite culture without a helper bacterium. For this purpose, we extracted genomic DNA from all involved organisms under different conditions. We used the genomic DNA to quantify *M. lacteus*, *P. protegens,* and *C. reinhardtii* with organism-specific primers via qPCR (34). The data obtained via qPCR was then converted into cell densities using correlation curves between cell density and Ct value (*SI Appendix*, Fig. S7 *B* and *C* and *Methods*). Cell densities in the bi- and tripartite cultures changed over time (Fig. 2 *B* and *C* and *SI Appendix*, Fig. S7). In the tripartite cultures (Fig. 2*C* and *SI Appendix*, Fig. S7*B*), both bacteria increased in cell density within the first 4 d of the experiment compared to inoculation density. It should be noted that the qPCR-calculated cell densities of the *M. lacteus* starting cultures were lower in all three replicates than the optically determined concentration of the stock before preparing the dilutions, yet the increase over the first 4 d was clearly visible in each case. In both the bipartite and tripartite cultures, the calculated cell densities of *C. reinhardtii* decreased during the first days and increased at the end of the second week (Fig. 2 *B* and *C* and *SI Appendix*, Fig. S7). At least a low amount of DNA from *C. reinhardtii* was detected via qPCR throughout the whole experiment, even when the alga could not be detected by visual observation based on the green color of the cultures and therefore was considered dead. It may be that some part of the genomic DNA of *C. reinhardtii* is derived from already lysed cells. However, the increase in algal cell density occurred earlier and was stronger in the tripartite cultures, whereas in the bipartite cultures, the effect was not visible at all or only very late. These data corroborate the role of *M. lacteus* as a helper bacterium.

We also examined whether related species of *Pseudomonas* and *Mycetocola* may have functions as algicidal and helper bacteria. As *Mycetocola* species have an inactivating effect on the CLiP tolaasin I (12), we investigated whether *C. reinhardtii* is influenced by *P. tolaasii*. Cocultures of *C. reinhardtii* with *P. tolaasii* revealed bleaching of the algal cells from the very beginning of the coculture (Fig. 2*D* and *SI Appendix*, Fig. S8) as observed with *P. protegens* (Fig. 2*A*). In tripartite cultures with *M. tolaasinivorans*, the algal cells recovered after 15 to 16 d (Fig. 2*D* and *SI Appendix*, Fig. S8). These data reveal that other *Pseudomonas* species can also act algicidal and that other *Mycetocola* species also function as helper bacteria. To find out whether these genera are closely together in nature, we reanalyzed some microbiota for their presence (*SI Appendix*, *Methods*) and found all three genera (*Chlamydomonas*, *Pseudomonas,* and *Mycetocola*) together (*Discussion*).

### The Helper Bacterium *M. lacteus* Linearizes Orfamide A at Its Ester Bond and Additionally Produces a Double-Cleaved Product.

**Fig. 3.**
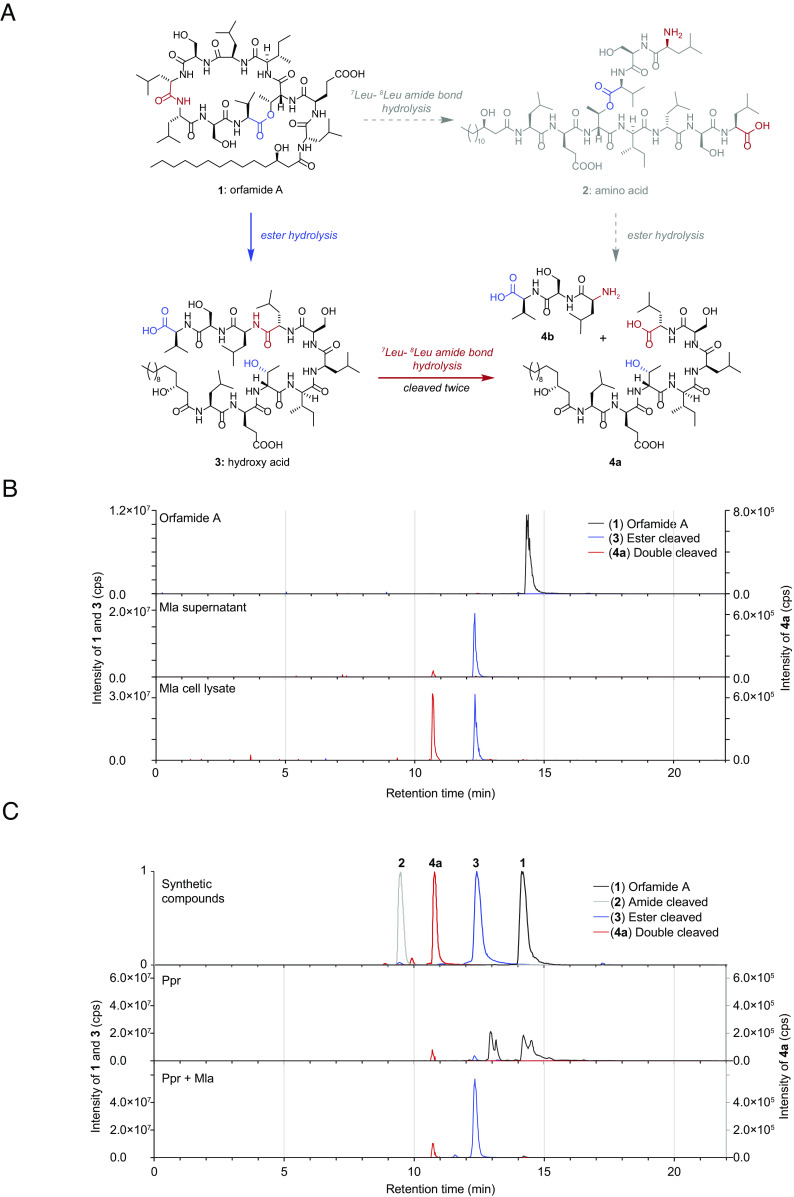
*M. lacteus* produces enzymes cleaving orfamide A into a linear form at its C-terminal ester bond and in a double-cleaved form lacking the three *C*-terminal amino acids. (*A*) Orfamide A (35) and putative degradation products of orfamide A that were chemically synthesized. (*B*): LC-HRMS showing the degradation of intact orfamide A (*m/z* 1295.84 [*M* + H]^+^; compound **1**) to either orfamide A with one cleavage (*m/z* 1313.85 [*M* + H]^+^; compound **2** or **3**) and orfamide A with two cleavages missing the *C*-terminal residues Leu-Ser-Val (*m/z* 1014.66 [*M* + H]^+^, compound **4a**) after 24 h incubation with the spent media (Mla supernatant) of the overnight culture of *M. lacteus* in LB or its cell lysate (see *SI Appendix*, *Methods* for details). (*C*): The *Upper* part shows LC-HRMS of chemically synthesized compounds, including intact orfamide A (*m/z* 1295.84 [*M* + H]^+^) and variants of the single cleaved degradation products (*m*/z 1313.85 [*M* + H]^+^) at the ester or amide bond as well as the double-cleaved degradation product missing the *C*-terminal residues Leu-Ser-Val (*m/z* 1014.66 [*M* + H]^+^). Below are cleaved orfamide A produced in bacterial coculture with *M. lacteus* (Mla) and *P. protegens* (Ppr) compared to a Ppr monoculture.

The CLiP orfamide A is a potent and fast-acting toxin of *P. protegens* (20, 22). As it was found that *M. lacteus* can cleave the CLiP tolaasin I enzymatically (12), we analyzed whether *M. lacteus* can also cleave orfamide A (Fig. 3*A*). Therefore, we incubated 20 μM orfamide A for 24 h with i) a *M. lacteus* cell lysate prepared from the cells of an overnight culture as well as with ii) the spent media where *M. lacteus* was grown overnight. The samples were analyzed using Liquid Chromatography-High Resolution Mass Spectrometry (LC-HRMS) for cleavage products of orfamide A.

In both cases, only minor traces of intact orfamide A with a *m/z* 1295.84 [*M* + H]^+^ (compound **1**, chemical formula: C_64_H_114_N_10_O_17_; exact mass: 1294.8363) were detected (Fig. 3 *A* and *B* and *SI Appendix*, Fig. S9). Instead, two major degradation products were observed (**3** and **4a**). In both extracts, a product with a mass shift of +18.01 Da (*m/z* 1313.85 [*M* + H]^+^) was observed, corresponding to the addition of water and therefore pointing to a hydrolysis reaction. In the cell lysate, the second cleavage product was as abundant with a mass shift of −281.18 Da (*m/z* 1014.66 [*M* + H]^+^; Fig. 3 *A* and *B* and *SI Appendix*, Fig. S9), corresponding to a product missing the C-terminal tripeptide. This suggestion was verified by MS/MS analyses (*SI Appendix*, Fig. S10).

To determine the position of the initially hydrolyzed bond, we chemically synthesized the putative cleavage products **2** (chemical formula: C_64_H_116_N_10_O_18_; exact mass: 1312.8469) and **3** (chemical formula: C_64_H_116_N_10_O_18_; exact mass: 1312.8469, in Fig. 3*A*). These compounds were used as references for comparative LC-HRMS/MS analysis (Fig. 3*C*, upper chromatogram; *SI Appendix*, Fig. S11). Furthermore, the double-cleaved product **4a** (chemical formula: C_50_H_91_N_7_O_14_; exact mass: 1013.6624), Fig. 3 *A* and *C*) was synthesized. It was found that orfamide A is hydrolyzed at the ester bond (compound **3**, Fig. 3*A*), which we further verified by comparing the MS/MS patterns of both ester- and amide-cleaved compounds with the patterns from the compounds in the *M. lacteus* supernatant and cell lysate, respectively (*SI Appendix*, Fig. S11 and *Methods*).

Moreover, we found that the ester cleaved form of orfamide A (compound **3**) is observed in a coculture of *P. protegens* and *M. lacteus*, as well as the double-cleaved form (compound **4a**), but in smaller amounts (Fig. 3*C* and *SI Appendix*, Fig. S12). The alterative compound **2** could not be detected, neither in the supernatant, nor in the cell lysate, suggesting that ester cleavage must precede peptide hydrolysis.

We also analyzed whether *M. tolaasinivorans* (spent medium and cell lysate) can cleave orfamide A (*SI Appendix*, Fig. S13). However, in this case, only the ester cleaved form was found in significant amounts in both cases. We detected no cleavage of orfamide A in the supernatant (spent medium) of *M. lacteus* and *M. tolaasinivorans* when heat-treated samples (*SI Appendix*, *Methods*) were used, suggesting that the secreted compound cleaving orfamide A is an enzyme (*SI Appendix*, Fig. S14). In the cell lysate, we observed a small amount of cleavage of orfamide A even after heat treatment, indicating that the length of the heat treatment may have been insufficient to fully inactivate the enzyme putatively involved in the process. The double-cleaved form has not been found in the cell lysate after heat treatment (*SI Appendix*, Fig. S14), suggesting again an enzymatic action.

To narrow down the enzymes potentially involved in the single and double cleavages, we performed a phylogenetic analysis of genes encoding hydrolases from *M. lacteus* that may be able to cleave orfamide A (*SI Appendix*, Fig. S15). It revealed 16 candidate genes in the genome of *M. lacteus* encoding mainly peptidases and esterases.

### The Helper Bacterium *M. lacteus* Biologically Inactivates Orfamide A by Ester Hydrolysis.

Our data so far indicate that two enzymes may be involved in the cleavage of orfamide A in *M. lacteus*. The first enzyme is released to the media. This hydrolase cleaves the ester bond of the macrolactone ring and produces the main cleavage product **3** in vivo in coculture of *P. protegens* and *M. lacteus* (Fig. 3*C*). The second enzyme seems to be located in the cytosol or at the inner membrane of *M. lacteus* because the corresponding cleavage product **4a** was only detectable in the cell lysate. This putative peptidase cleaves the three *C*-terminal residues Leu-Ser-Val but only after the macrolactone ring has been opened.

**Fig. 4.**
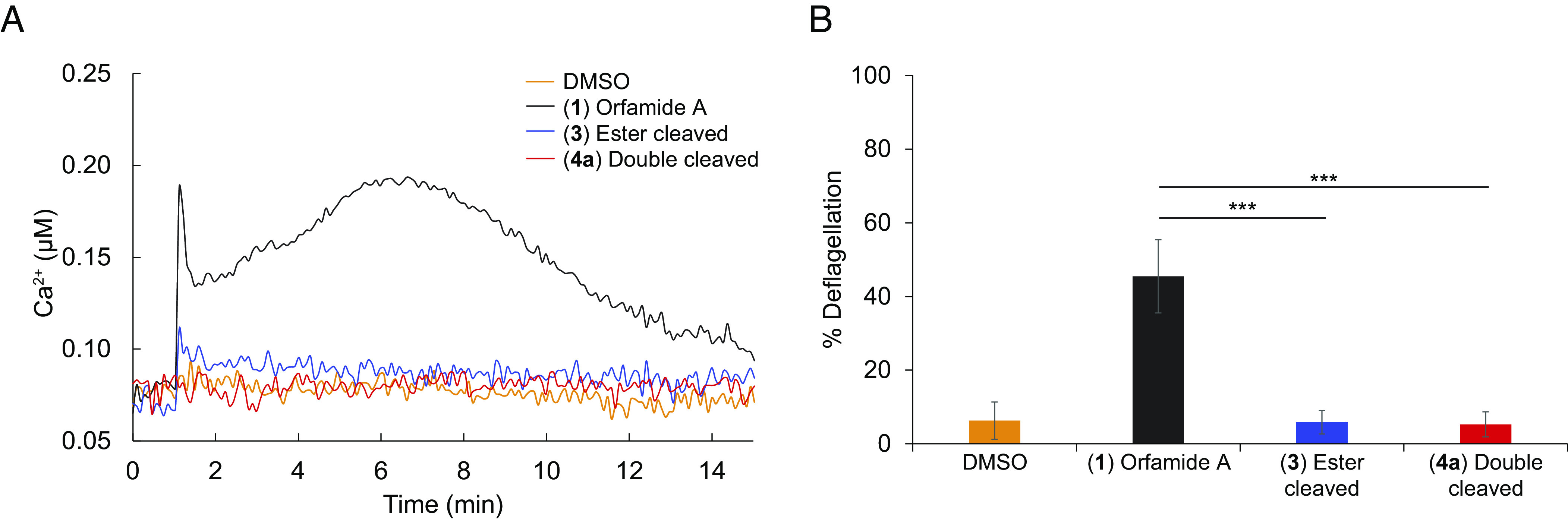
*M. lacteus* biologically inactivates orfamide A by cleavage. (*A*): The algal Ca^2+^ signature was investigated using a *C. reinhardtii* aequorin reporter line (see *SI Appendix, Methods* for details). Orfamide A (compound **1** from Fig. 3*A*) and its chemically synthesized degradation products (ester-cleaved compound **3** and double-cleaved compound **4a** from Fig. 3*A*) were dissolved in DMSO and further diluted in TAP medium to a final concentration of 5 μM. As control, DMSO proportional to 5 μM of compound was used. Each line represents the mean of three independent biological replicates, and each biological replicate includes three technical replicates. (*B*) Deflagellation rates of wild-type algal cells treated with 5 μM intact orfamide A and its degradation products (see panel *A* for details). DMSO proportional to 5 μM of compounds was used as control. More than 100 cells were counted for each sample. Each column represents the mean of at least three independent biological replicates. Error bars represent SDs; Student’s *t* test was performed; n.s., not significant and ***P < 0.001.

Finally, we characterized the biological activity of the determined single- and double-cleaved orfamide A derivatives by using the chemically synthesized reference compounds. For this purpose, we utilized a transgenic algal line expressing apo-aequorin as reporter (24) to measure cytosolic Ca^2+^ levels upon exposure to orfamide A and its derivatives. In contrast to the natural CLiP orfamide A (compound **1**), both the single- (compound **3** in Fig. 3*A*) and double-cleaved orfamide A variants (compound **4a** in Fig. 3*A*) did not trigger an increase in cytosolic Ca^2+^ levels (Fig. 4*A*). Accordingly, unlike natural orfamide A, these variants did not cause significant deflagellation (Fig. 4*B*). Our findings show that both cleavage products of orfamide A have lost their ability to interfere with the Ca^2+^ signaling pathways in *C. reinhardtii* and hence their toxicity.

## Discussion

**Fig. 5.**
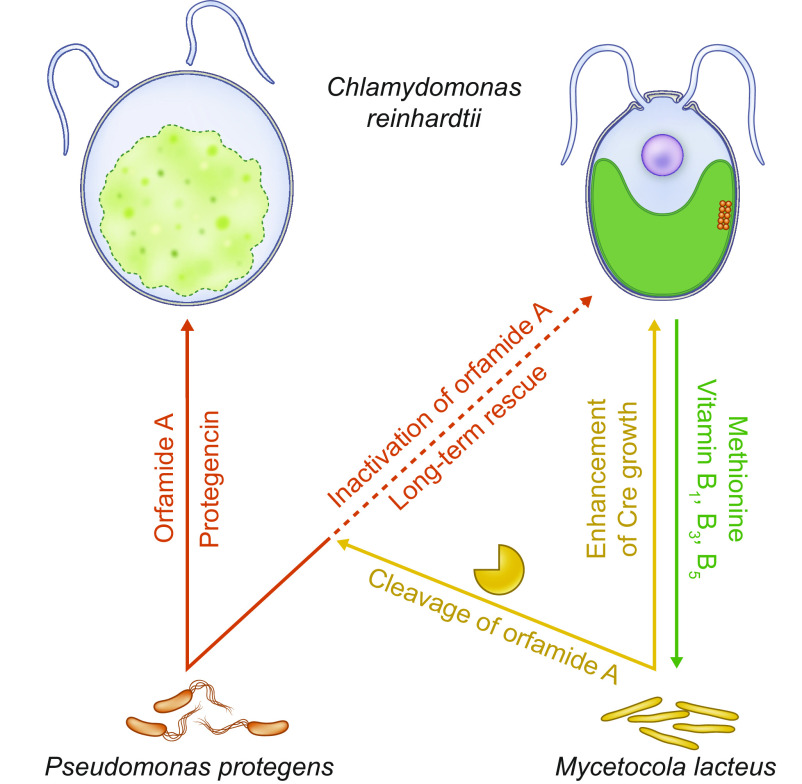
The helper bacterium *M. lacteus* uses metabolites secreted by *C. reinhardtii* for its growth, enhances algal growth, and rescues the alga from the attack by *P. protegens*. The antagonistic bacterium *P. protegens* deflagellates, bleaches, and lyses the alga (orange arrow). Pictures are illustrated based on a 24 h bacterial attack of the algae (21). *M. lacteus* has a dual function: i) it promotes the growth of *C. reinhardtii* (yellow arrow), and it ii) rescues the alga from *P. protegens* (dashed orange arrow) by cleaving orfamide A (yellow arrow), and thus inactivating this CLiP (dashed orange arrow). *Chlamydomonas* releases specific B vitamins and methionine, which can be utilized as an organic sulfur source to support the growth of *M. lacteus* (green arrow).

In the past years, the green microalga *C. reinhardtii* has emerged as a model to study microbial interactions (36). Here, we have shown that *M. lacteus* from the phylum Actinomycetota can live in a mutualistic partnership with this green alga. *M. lacteus* needs organic sulfur compounds and the vitamins B_1_, B_3_, B_5_, and B_7_ for growth, with vitamins B_3_ and B_5_ being essential and B_1_ being needed for sustained growth (Figs. 1 and 5). *C. reinhardtii* has been reported to secrete vitamins B_5_ (pantothenate), B_7_ (biotin), and B_9_ (folate) (37). The release of vitamin B_3_ (nicotinic acid) was shown earlier for the related *C. eugametos* (37–40). Moreover, the production and uptake of vitamin B_1_ by *C. reinhardtii* was known (40), but not its secretion. Here, we found that *C. reinhardtii* releases vitamins B_1_, B_3_, and B_5_. Our data suggest further that all are taken up by *M. lacteus* (*SI Appendix*, Table S1). We did not find any release of vitamin B_7_, which was analyzed before only in a heterologous system (37). In addition to the B vitamins, *M. lacteus* needs organic sulfur for growth, and it cannot use the sulfate present in the algal TAP medium. Notably, *C. reinhardtii* can secrete methionine into the medium. Auxotrophy for methionine has been reported for some bacteria and is associated with specific bacterial methionine-based affinity transporters (41). However, *M. lacteus* is not auxotrophic for methionine as it can utilize organic sulfur compounds to sustain its growth (*SI Appendix*, Fig. S3). In the coculture of *M. lacteus* and *C. reinhardtii*, the alga secretes methionine into the media, which the bacterial partner can use as a source of organic sulfur.

As in a true mutualistic partnership, *C. reinhardtii* profits from the presence of *M. lacteus*, as indicated by a significant increase in cell counts (Figs. 1*B* and 5). However, it is important to note that the algae can also grow without the bacterium. We do not yet know how the bacteria stimulate algal growth or which compounds are involved. One possibility is that CO_2_ released by the bacteria stimulates the algal growth as shown in some cases (42).

*M. lacteus* has a dual role for the algal cells. It not only supports algal growth but also functions as a helper bacterium in the tripartite cultures with the algicidal bacterium *P. protegens*. For these, it inactivates the chemical mediator orfamide A (Fig. 5). The initial step, suggested to be carried out by a heat-labile hydrolase enzyme released into the supernatant, inactivates the primary biological function of this CLiP. A second potential enzyme associated with the cell lysate results in the double-cleaved peak **4a**. This product does not show up after heat treatment, suggesting also in this case the action of a heat-labile enzyme (*SI Appendix*, Fig. S14). The observed cleavage site selectivity may be a result of residue stereochemistry. Peptidases that will hydrolyze amide bonds adjacent to D-amino acid residues are very rare. Only at the cleavage site observed, two L-amino acids occur consecutively. A phylogeny screen suggested at least 16 hydrolase enzymes of *M. lacteus* that may be potentially involved in cleaving orfamide A (*SI Appendix*, Fig. S15 and *Methods*). Linearization of the CLiP alone is enough to prevent an increase in cytosolic Ca^2+^ and the consequent deflagellation of the algal cells. Consequently, the algae remain motile and can swim away from the antagonistic bacterium. We hypothesize that the circling of the algal cells by *P. protegens* and their exposure to the bacterial toxic cocktail of secondary metabolites (20–22) is thus reduced. In addition, they can use their cilia to attach to surfaces such as the walls of culture vessels or soil particles in nature, where bacteria can attack only from one side and where the algae are thus less exposed to their toxins. Altogether, these steps contribute to enabling their recovery in the long term.

The two CLiPs pseudodesmin A and tolaasin I, both produced by *P. tolaasii*, are cleaved by enzymes of *M. lacteus* and *M. tolaasinivorans* as well, rescuing the mushroom *A. bisporus* from the attack of *P. tolaasii* (12). Interestingly, *P. tolaasii* acts as an antagonistic bacterium for *C. reinhardtii*. *M. tolaasinivorans* rescues *C. reinhardtii* (Fig. 2*D*), functioning as a helper bacterium in this case as well. Altogether, *M. lacteus* and *M. tolaasinivorans* (12) seem to produce enzymes that cleave and thus detoxify different bacterial CLiPs, of which some are secreted to the medium. Indeed, we found that *M. tolaasinivorans* linearizes orfamide A, but a double-cleaved product was not found in this case (*SI Appendix*, Fig. S13). Intriguingly, both *M. lacteus* and *M. tolaasinivorans* function as helper bacteria in bacterial-fungal (12) as well as in bacterial-algal interactions by using the same mechanistic principle. They both inactivate CLiPs by lactone hydrolysis. Given that orfamide A also immobilizes other ciliated Chlorophyceae algae (20), the described inactivation mechanism holds broad ecological significance. Moreover, all three genera (*Chlamydomonas*, *Pseudomonas* and *Mycetocola*) cooccur in nature. *C. reinhardtii* was first isolated in a potato field (30). Microbiota in the potato rhizosphere of High Andes include *Pseudomonas* as well as *Mycetocola* genera along with *Chlamydomonas* [(43); *SI Appendix*, *Methods*]. In this context, it is also worth noting that sugars including glucose are supplied via root exudates (44–46) in potato fields, as *C. reinhardtii* is not releasing any or sufficient carbon source for growth of *M. lacteus* (Fig. 1*E*). *M. lacteus* and *P. protegens* can both grow on sugars, including glucose as carbon source (31, 47). In addition, all three genera (*Chlamydomonas*, *Pseudomonas,* and *Mycetocola*) are present in wash water of another crop, rice (*SI Appendix*, *Methods*). Remarkably, a *C. reinhardtii* strain recruits actively both bacterial genera (*Pseudomonas* and *Mycetocola*) among others, when cultured in natural soil [(19); *SI Appendix, Methods*].

Investigating research to tripartite microbial interactions in addition to dual cultures is a first important step to decode the complexity of microbial interactions, along with their chemical mediators and ecological relevance in nature. Tripartite interactions with microorganisms have been studied so far only in a few cases, but these examples already highlight their ecological potential. Thus, interactions of three different bacterial species suggest that community composition and microbiome interactions shape the regulation of secondary metabolites (48), an emerging field in natural product research (13). The macroalga *Ulva,* involved in green algal blooms, can only grow and develop efficiently in the presence of two different types of bacteria and involved secondary metabolites (49). Maintenance of coral–algae–microbe interactions can be used to cope with ocean acidification (50). In the future, it will be essential to understand such multipartner microbial interactions, along with their provoked changes in secondary metabolites, to better understand microorganisms and their relevance for life on Earth. This holds especially true for algal–microbial interactions and their role in shaping algal fitness, as algae are key primary producers, providing O_2_ and fixing CO_2_ on Earth.

## Materials and Methods

The *C. reinhardtii* wild-type strain SAG 73.72 and bacteria (*P. protegens* PF-5, *P. tolaasii*, *M. lacteus* and *M. tolaasinivorans*, respectively) were grown either in mono-, bi-, or tripartite cultures in TAP medium (30) in the presence of 0.2% (w/v) glucose and in some cases at a lower phosphate concentration of 0.1 mM phosphate, unless otherwise stated. For some experiments, cultures were supplemented with B vitamins and/or amino acids, as indicated. Methionine and cysteine as well as B vitamins were analyzed by HPLC and respective *E. coli* mutants in case of the amino acids.

Orfamide A cleavage was analyzed by LC-HRMS (MS) measurements. A phylogeny of hydrolases from *M. lacteus* potentially cleaving orfamide A was performed (*SI* FASTA file Multiple Sequence Alignment_Phylogenetic Tree). Degradation products of orfamide A were chemically synthesized and used as standards and for functional analysis along with an aequorin-based Ca^2+^ assay.

Three microbiotas were reanalyzed for the presence of the genera *Pseudomonas* and *Mycetocola* along with *Chlamydomonas*.

A detailed description of materials and methods can be found in *SI Appendix*, together with additional figures and tables, and a Dataset S1.

## Supplementary Material

Appendix 01 (PDF)

Dataset S01 (TXT)

## Data Availability

*SI* FASTA file Multiple Sequence Alignment_Phylogenetic Tree can be found in Dataset S1. All other data are included in the manuscript and/or supporting information. Previously published sequencing data was also used for this work. The original publications for these data are available in *SI Appendix*. All of them are also available through the Sequence Read Archive of the International Nucleotide Sequence Database Collaboration (51).

## References

[1] C. B. Field, M. J. Behrenfeld, J. T. Randerson, P. Falkowski, Primary production of the biosphere: Integrating terrestrial and oceanic components. Science **281**, 237–240 (1998). 9657713 10.1126/science.281.5374.237

[2] E. Cirri, G. Pohnert, Algae-bacteria interactions that balance the planktonic microbiome. New Phytol. **223**, 100–106 (2019). 30825329 10.1111/nph.15765

[3] E. F. Y. Hom, A. W. Murray, Niche engineering demonstrates a latent capacity for fungal-algal mutualism. Science **345**, 94–98 (2014). 24994654 10.1126/science.1253320 PMC4409001

[4] M. B. Cooper, A. G. Smith, Exploring mutualistic interactions between microalgae and bacteria in the omics age. Curr. Opin. Plant Biol. **26**, 147–153 (2015). 26318329 10.1016/j.pbi.2015.07.003

[5] V. Calatrava , A new mutualism between *Chlamydomonas* and methylobacteria facilitates growth on amino acids and peptides. FEMS Microbiol. Lett. **365**, fny021 (2018). 10.1093/femsle/fny021 29385570

[6] S. Venkataram, H.-Y. Kuo, E. F. Y. Hom, S. Kryazhimskiy, Mutualism-enhancing mutations dominate early adaptation in a two-species microbial community. Nat. Ecol. Evol. **7**, 143–154 (2023). 36593292 10.1038/s41559-022-01923-8

[7] K. E. Helliwell , Fundamental shift in vitamin B_12_ eco-physiology of a model alga demonstrated by experimental evolution. ISME J. **9**, 1446–1455 (2015). 25526368 10.1038/ismej.2014.230 PMC4430296

[8] M. B. Cooper , Cross-exchange of B-vitamins underpins a mutualistic interaction between *Ostreococcus tauri* and *Dinoroseobacter shibae*. ISME J. **13**, 334–345 (2019). 30228381 10.1038/s41396-018-0274-y PMC6331578

[9] K. J. Coyne, Y. Wang, G. Johnson, Algicidal bacteria: A review of current knowledge and applications to control harmful algal blooms. Front. Microbiol. **13**, 871177 (2022). 35464927 10.3389/fmicb.2022.871177 PMC9022068

[10] M. K. C. Krespach , Lichen-like association of *Chlamydomonas reinhardtii* and *Aspergillus nidulans* protects algal cells from bacteria. ISME J. **14**, 2794–2805 (2020). 32753730 10.1038/s41396-020-0731-2 PMC7784976

[11] K. M. Dahlstrom, D. K. Newman, Soil bacteria protect fungi from phenazines by acting as toxin sponges. Curr. Biol. **32**, 275–288.e5 (2022). 34813731 10.1016/j.cub.2021.11.002 PMC8792240

[12] R. Hermenau, S. Kugel, A. J. Komor, C. Hertweck, Helper bacteria halt and disarm mushroom pathogens by linearizing structurally diverse cyclolipopeptides. Proc. Natl. Acad. Sci. U.S.A. **117**, 23802–23806 (2020). 32868430 10.1073/pnas.2006109117 PMC7519232

[13] P. Stallforth, M. Mittag, A. A. Brakhage, C. Hertweck, U. A. Hellmich, Functional modulation of chemical mediators in microbial communities. Trends Biochem. Sci. **48**, 71–81 (2023). 35981931 10.1016/j.tibs.2022.07.006

[14] S. Sasso, H. Stibor, M. Mittag, A. R. Grossman, From molecular manipulation of domesticated *Chlamydomonas reinhardtii* to survival in nature. Elife **7**, e39233 (2018). 30382941 10.7554/eLife.39233 PMC6211829

[15] P. A. Salomé, S. S. Merchant, A series of fortunate events: Introducing *Chlamydomonas* as a reference organism. Plant Cell **31**, 1682–1707 (2019). 31189738 10.1105/tpc.18.00952 PMC6713297

[16] R. J. Craig , The *Chlamydomonas* Genome Project, version 6: Reference assemblies for mating-type *plus* and *minus* strains reveal extensive structural mutation in the laboratory. Plant Cell **35**, 644–672 (2023). 36562730 10.1093/plcell/koac347 PMC9940879

[17] C. Hesse , Genome-based evolutionary history of *Pseudomonas* spp. Environ. Microbiol. **20**, 2142–2159 (2018). 29633519 10.1111/1462-2920.14130

[18] A. Peix, M.-H. Ramírez-Bahena, E. Velázquez, The current status on the taxonomy of *Pseudomonas* revisited: An update. Infect. Genet. Evol. **57**, 106–116 (2018). 29104095 10.1016/j.meegid.2017.10.026

[19] P. Durán , Shared features and reciprocal complementation of the *Chlamydomonas* and *Arabidopsis* microbiota. Nat. Commun. **13**, 406 (2022). 35058457 10.1038/s41467-022-28055-8 PMC8776852

[20] P. Aiyar , Antagonistic bacteria disrupt calcium homeostasis and immobilize algal cells. Nat. Commun. **8**, 1756 (2017). 29170415 10.1038/s41467-017-01547-8 PMC5701020

[21] V. Hotter , A polyyne toxin produced by an antagonistic bacterium blinds and lyses a *Chlamydomonad* alga. Proc. Natl. Acad. Sci. U.S.A. **118**, e2107695118 (2021). 34389682 10.1073/pnas.2107695118 PMC8379975

[22] M. M. Rose , The bacterium *Pseudomonas protegens* antagonizes the microalga *Chlamydomonas reinhardtii* using a blend of toxins. Environ. Microbiol. **23**, 5525–5540 (2021). 34347373 10.1111/1462-2920.15700

[23] D. Wu , Ciliogenesis requires sphingolipid-dependent membrane and axoneme interaction. Proc. Natl. Acad. Sci. U.S.A. **119**, e2201096119 (2022). 35895683 10.1073/pnas.2201096119 PMC9351462

[24] Y. Hou , A cyclic lipopeptide produced by an antagonistic bacterium relies on its tail and transient receptor potential-type Ca ^2+^ channels to immobilize a green alga. New Phytol. **237**, 1620–1635 (2023). 36464797 10.1111/nph.18658

[25] T. Tsukamoto, M. Takeuchi, O. Shida, H. Murata, A. Shirata, Proposal of *Mycetocola* gen. nov. in the family *Microbacteriaceae* and three new species, *Mycetocola saprophilus* sp. nov., *Mycetocola tolaasinivorans* sp. nov. and *Mycetocola lacteus* sp. nov., isolated from cultivated mushroom, *Pleurotus ostreatus*. Int. J. Syst. Evol. Microbiol. **51**, 937–944 (2001). 11411718 10.1099/00207713-51-3-937

[26] T. Tsukamoto, H. Murata, A. Shirata, Identification of non-pseudomonad bacteria from fruit bodies of wild agaricales fungi that detoxify tolaasin produced by *Pseudomonas tolaasii*. Biosci. Biotechnol. Biochem. **66**, 2201–2208 (2002). 12450133 10.1271/bbb.66.2201

[27] X. Luo , *Mycetocola manganoxydans* sp. nov., an actinobacterium isolated from the Taklamakan desert. Int. J. Syst. Evol. Microbiol. **62**, 2967–2970 (2012). 22286906 10.1099/ijs.0.038877-0

[28] S. A. Turlapati, R. Minocha, S. Long, J. Ramsdell, S. C. Minocha, Oligotyping reveals stronger relationship of organic soil bacterial community structure with N-amendments and soil chemistry in comparison to that of mineral soil at Harvard Forest, MA, USA. Front. Microbiol. **6**, 00049 (2015). 10.3389/fmicb.2015.00049 PMC4329816 25762982

[29] Y. Chen, T. Pan, G. Chai, Z. Li, Complete genome of *Mycetocola spongiae* MSC19T isolated from deep-sea sponge *Cacospongia mycofijiensis* indicates the adaptation to deep-sea environment and sponge-microbe symbioses. Mar. Genomics **63**, 100955 (2022). 35568404 10.1016/j.margen.2022.100955

[30] E. H. Harris, The Chlamydomonas Sourcebook: A Comprehensive Guide to Biology and Laboratory Use (Academic Press Inc, 1989). 10.1126/science.246.4936.1503-a 17756009

[31] L. Zhu , *Mycetocola miduiensis* sp. nov., a psychrotolerant bacterium isolated from Midui glacier. Int. J. Syst. Evol. Microbiol. **63**, 2661–2665 (2013). 23291895 10.1099/ijs.0.047985-0

[32] E. Kazamia , Mutualistic interactions between vitamin B_12_-dependent algae and heterotrophic bacteria exhibit regulation. Environ. Microbiol. **14**, 1466–1476 (2012). 22463064 10.1111/j.1462-2920.2012.02733.x

[33] M. Bains, L. Fernández, R. E. W. Hancock, Phosphate starvation promotes swarming motility and cytotoxicity of *Pseudomonas aeruginosa*. Appl. Environ. Microbiol. **78**, 6762–6768 (2012). 22773629 10.1128/AEM.01015-12 PMC3426718

[34] P. Kralik, M. Ricchi, A basic guide to real time PCR in microbial diagnostics: Definitions, parameters, and everything. Front. Microbiol. **8**, 00108 (2017). 10.3389/fmicb.2017.00108 PMC5288344 28210243

[35] Y. Bando , Total synthesis and structure correction of the cyclic lipodepsipeptide orfamide A. Chemistry **28**, e202104417 (2022). 35199896 10.1002/chem.202104417 PMC9311703

[36] V. Calatrava , *Chlamydomonas reinhardtii*, a reference organism to study algal–microbial interactions: Why can’t they be friends? Plants **12**, 788 (2023). 36840135 10.3390/plants12040788 PMC9965935

[37] S. Aaronson , The cell content and secretion of water-soluble vitamins by several freshwater algae. Arch. Microbiol. **112**, 57–59 (1977). 402897 10.1007/BF00446654

[38] K. Nakamura, C. S. Gowans, Nicotinic acid-excreting mutants in *Chlamydomonas*. Nature **202**, 826–827 (1964).

[39] D. J. Uhlik, C. S. Gowans, Synthesis of nicotinic acid in *Chlamydomonas eugametos*. Int. J. Biochem. **5**, 79–84 (1974).

[40] M. Moulin, G. T. D. T. Nguyen, M. A. Scaife, A. G. Smith, T. B. Fitzpatrick, Analysis of *Chlamydomonas* thiamin metabolism in vivo reveals riboswitch plasticity. Proc. Natl. Acad. Sci. U.S.A. **110**, 14622–14627 (2013). 23959877 10.1073/pnas.1307741110 PMC3767531

[41] J. A. Hernandez-Valdes, J. Van Gestel, O. P. Kuipers, A riboswitch gives rise to multi-generational phenotypic heterogeneity in an auxotrophic bacterium. Nat. Commun. **11**, 1203 (2020). 32139702 10.1038/s41467-020-15017-1 PMC7058034

[42] X. Bai, P. Lant, S. Pratt, The contribution of bacteria to algal growth by carbon cycling. Biotechnol. Bioeng. **112**, 688–695 (2015). 25312046 10.1002/bit.25475

[43] S. Pfeiffer , Rhizosphere microbiomes of potato cultivated in the High Andes show stable and dynamic core microbiomes with different responses to plant development. FEMS Microbiol. Ecol. **93**, fiw242 (2017). 27940644 10.1093/femsec/fiw242

[44] C. A. Bell, W. Mobayed, C. J. Lilley, P. E. Urwin, Monosaccharide constituents of potato root exudate influence hatching of the white potato cyst nematode. PhytoFrontiers **1**, 258–266 (2022).

[45] G. A. Hoysted, C. A. Bell, C. J. Lilley, P. E. Urwin, Aphid colonization affects potato root exudate composition and the hatching of a soil borne pathogen. Front. Plant Sci. **9**, 1278 (2018). 30237805 10.3389/fpls.2018.01278 PMC6136236

[46] A. S. Koroney , Root exudate of *Solanum tuberosum* is enriched in galactose-containing molecules and impacts the growth of *Pectobacterium atrosepticum*. Ann. Bot. **118**, 797–808 (2016). 27390353 10.1093/aob/mcw128 PMC5055634

[47] R. A. Wilkes, C. M. Mendonca, L. Aristilde, A cyclic metabolic network in *Pseudomonas protegens* Pf-5 prioritizes the Entner-Doudoroff pathway and exhibits substrate hierarchy during carbohydrate co-utilization. Appl. Environ. Microbiol. **85**, e02084-18 (2019). 30366991 10.1128/AEM.02084-18 PMC6293094

[48] M. G. Chevrette , Microbiome composition modulates secondary metabolism in a multispecies bacterial community. Proc. Natl. Acad. Sci. U.S.A. **119**, e2212930119 (2022). 36215464 10.1073/pnas.2212930119 PMC9586298

[49] T. Wichard, From model organism to application: Bacteria-induced growth and development of the green seaweed *Ulva* and the potential of microbe leveraging in algal aquaculture. Semin. Cell Dev. Biol. **134**, 69–78 (2023). 35459546 10.1016/j.semcdb.2022.04.007

[50] Z. Lin, L. Wang, M. Chen, X. Zheng, J. Chen, Proteome and microbiota analyses characterizing dynamic coral-algae-microbe tripartite interactions under simulated rapid ocean acidification. Sci. Total Environ. **810**, 152266 (2022). 34896508 10.1016/j.scitotenv.2021.152266

[51] G. Cochrane, I. Karsch-Mizrachi, T. Takagi, International Nucleotide Sequence Database Collaboration, The International Nucleotide Sequence Database Collaboration. Nucleic Acids Res. **44**, D48–D50 (2016). 26657633 10.1093/nar/gkv1323 PMC4702924

