# Supplementary material for: A mutualistic bacterium rescues a green alga from an antagonist
Source: Proc Natl Acad Sci U S A. 2024 Apr 3;121(15):e2401632121. doi: 10.1073/pnas.2401632121 (PMC11009677; doi:10.1073/pnas.2401632121)
Supplement: Supplementary file 1 — Appendix 01 (PDF) [file pnas.2401632121.sapp.pdf]

Supporting Information for
A mutualistic bacterium rescues a green alga from an antagonist

David Carrasco Flores, Vivien Hotter, Trang Vuong, Yu Hou, Yuko Bando, Kirstin Scherlach, Bertille Burgunter-Delamare, Ron Hermenau, Anna J. Komor, Prasad Aiyar, Magdalena Rose, Severin Sasso, Hans-Dieter Arndt, Christian Hertweck, Maria Mittag*

*Corresponding author: Maria Mittag
Email: M.Mittag@uni-jena.de

This PDF file includes:

Fig. S1 - Fig. S15 (pages 2-27)

Table S1 (page 28)

Material and Methods containing Fig. S16 - Fig. S22 and Table S2 (pages 29-56)

Table S3 (page 57-64)

References in *SI* Appendix (pages 65-66)

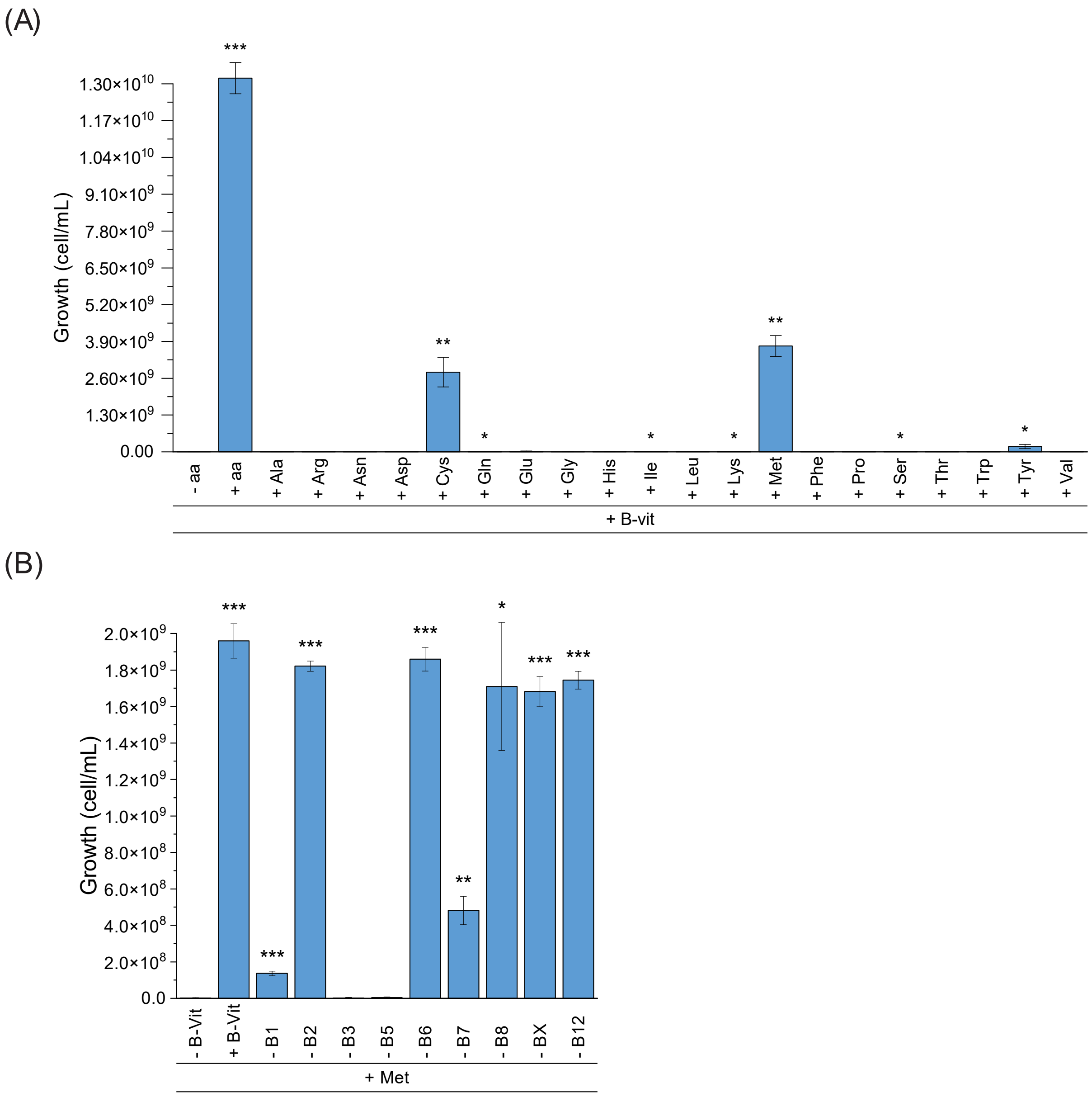

Fig. S1: Amino acids and B vitamins needed for the growth of *M. lacteus*.

(A) Most amino acids cannot supplement the growth of *M. lacteus*. TAP medium with 0.2% (w/v) glucose and a mixture of the nine B-vitamins was used as medium. To investigate the bacterium's need for amino acids, each *M. lacteus* culture was supplemented with either no amino acids (-aa), a mixture of the 20 amino acids (+aa) or one amino acid at a time. For details, see Materials and Methods. The experiments represent the average of three independent biological replicates with the error bars denoting the SD.

(B) *M. lacteus* needs certain B vitamins for its growth. TAP medium supplemented with 0.2% (w/v) glucose and methionine was used as medium. To test the bacterial B vitamin requirements, *M. lacteus* cultures were grown with either no B vitamins (- B-Vit), the mixture

of the nine B vitamins (+ B-Vit) or eight out of the nine B vitamins (one different vitamin always absent). See legend (A) for further details.

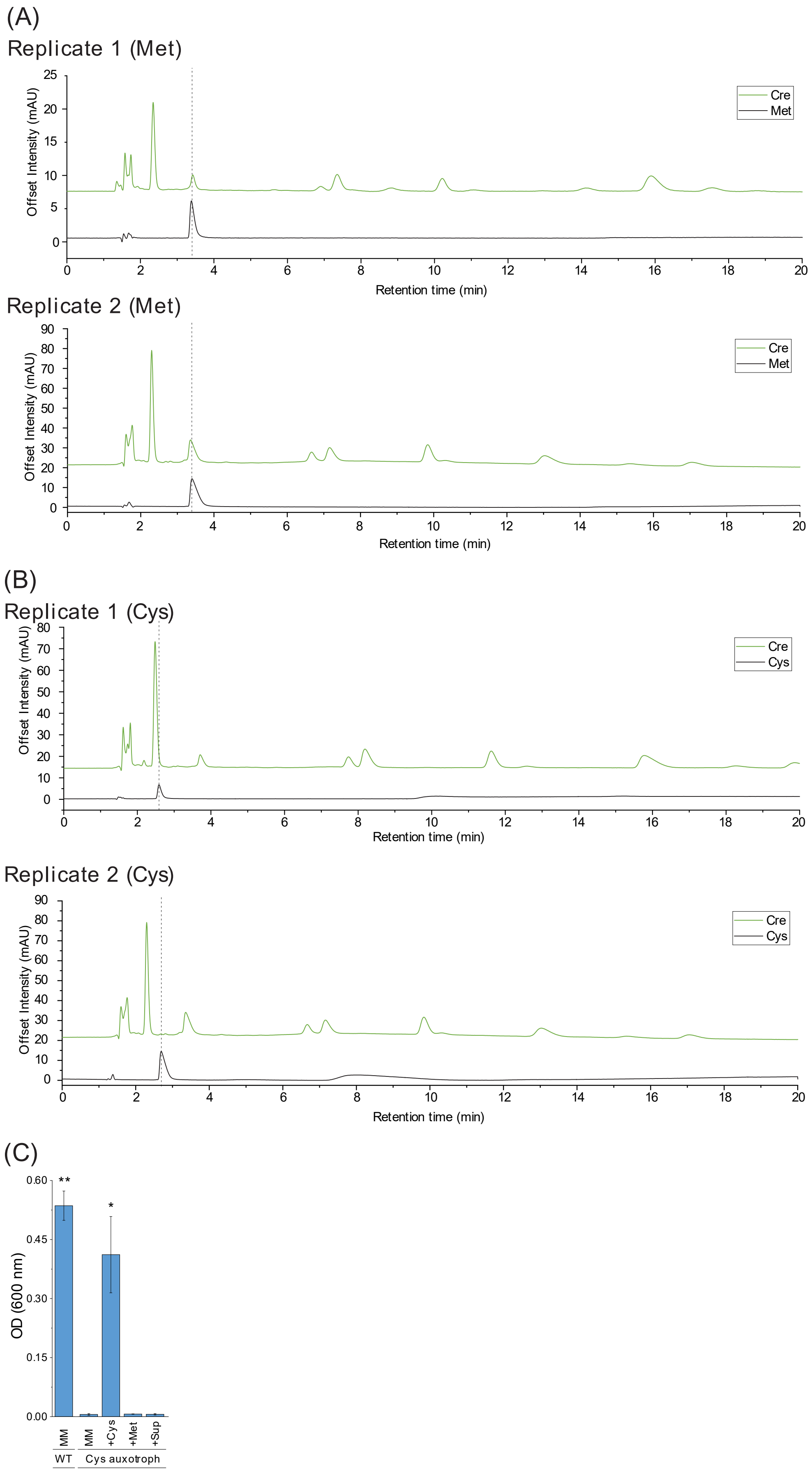
(A)
Replicate 1 (Met)
Offset Intensity (mAU)
Retention time (min)
Cre
Met
Replicate 2 (Met)
Offset Intensity (mAU)
Retention time (min)
Cre
Met
(B)
Replicate 1 (Cys)
Offset Intensity (mAU)
Retention time (min)
Cre
Cys
Replicate 2 (Cys)
Offset Intensity (mAU)
Retention time (min)
Cre
Cys
(C)
OD (600 nm)
**
*
MM
MM
+Cys
+Met
+Sup
WT
Cys auxotroph

Fig. S2: *C. reinhardtii* secretes extracellular methionine, but not cysteine to the culture medium.

(A) *C. reinhardtii* secretes extracellular methionine to the culture medium. HPLC chromatograms of a methionine standard and secreted extracellular amino acids extracted from spent medium of *C. reinhardtii* in axenic culture. The detection wavelength was 260 nm. The experiment was performed twice independently (biological replicates 1 and 2).

(B) *C. reinhardtii* does not secrete extracellular cysteine to the culture medium.

HPLC chromatograms of cysteine standards and secreted extracellular amino acids extracted from spent medium of *C. reinhardtii* in axenic culture. The experiment was performed twice independently (biological replicates 1 and 2).

(C) *C. reinhardtii* spent medium cannot complement an *E. coli* auxotroph mutant to produce cysteine. Comparison of the growth of *E. coli* wild type (WT) in M9 minimal medium (MM) with an *E. coli* auxotroph mutant for the production of cysteine in M9 minimal medium supplemented with either nothing, cysteine (Cys), methionine (Met) as further control or *C. reinhardtii* spent medium (supernatant, Sup); see Methods for details. The asterisk indicates significant differences between the growth of the Cys auxotroph bacteria in minimal medium and Cys supplemented cultures as calculated by Student's t-test (**: $P \leq 0.01$). The error bars indicate SDs with $n = 3$ biological replicates.

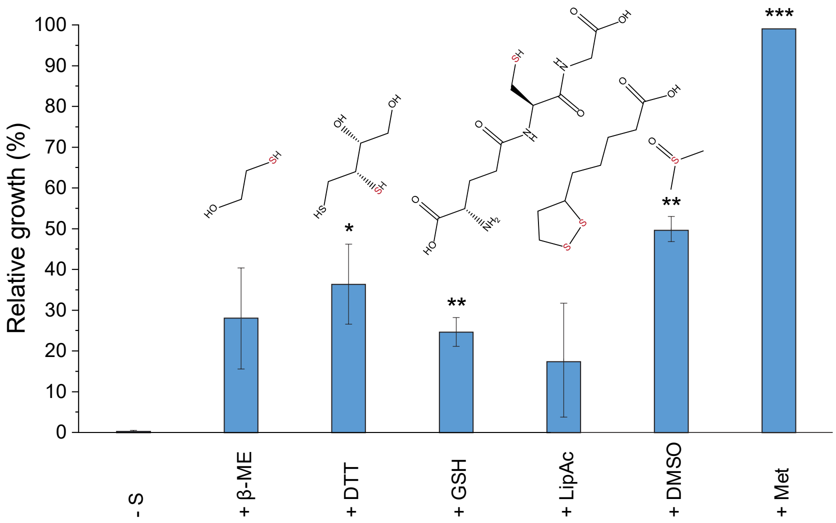

Fig. S3: *M. lacteus* can grow in the presence of organic sulfur compounds.

For the negative control (-S), no methionine was added. As positive control, TAP medium supplemented with 0.2% (w/v) glucose in the presence of methionine (+Met), B_1, B_3, B_5 and B_7 was used. The positive control also served as template for the tests of the individual sulfur compounds by replacing the methionine with 1 mM of the tested compound. ß-ME: β-mercaptoethanol, DTT: dithiothreitol, GSH: glutathione, LipAc: lipoic acid and DMSO: dimethyl sulfoxide.

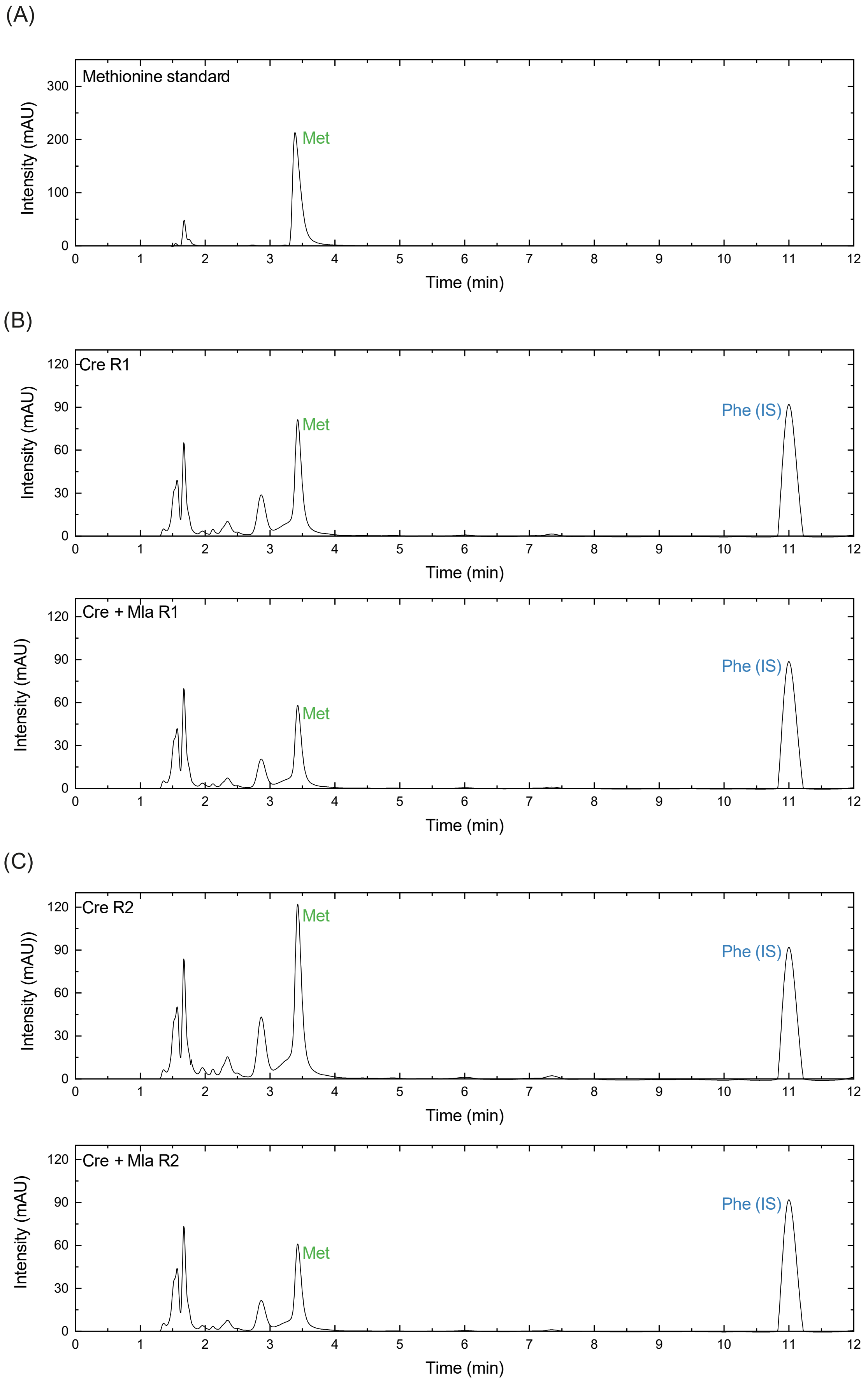
(A)
Methionine standard
Met
Intensity (mAU)
Time (min)
(B)
Cre R1
Met
Phe (IS)
Intensity (mAU)
Time (min)
Cre + Mla R1
Met
Phe (IS)
Intensity (mAU)
Time (min)
(C)
Cre R2
Met
Phe (IS)
Intensity (mAU))
Time (min)
Cre + Mla R2
Met
Phe (IS)
Intensity (mAU)
Time (min)

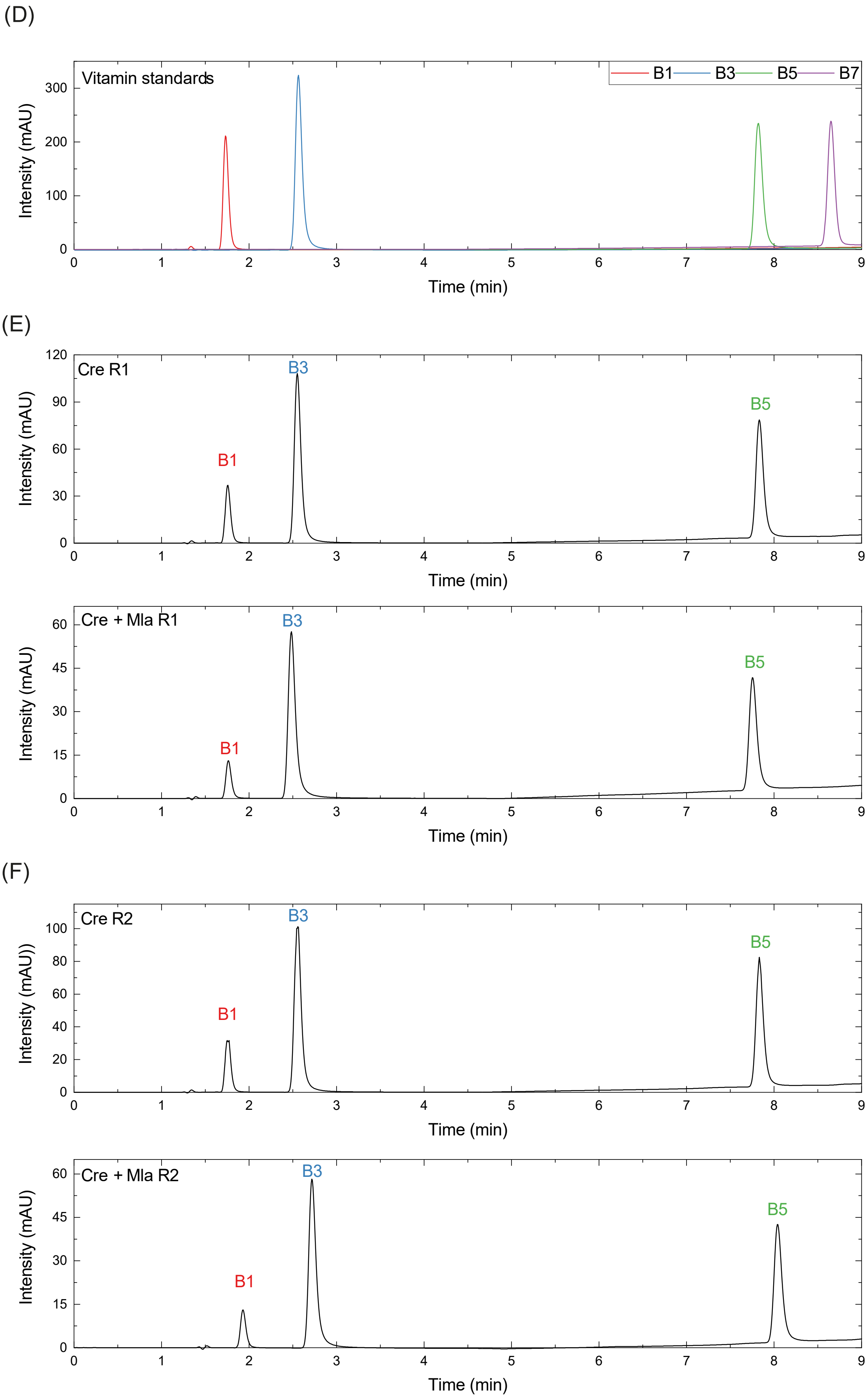

(D)
Vitamin standards
B1
B3
B5
B7
Intensity (mAU)
Time (min)
(E)
Cre R1
Cre + Mla R1
(F)
Cre R2
Cre + Mla R2

Fig. S4: Analysis of methionine and vitamins B_1, B_3, B_5 B_5 and B_7 in supernatants of cultures. HPLC chromatogram of methionine (A) and combined HPLC chromatograms of vitamins B_1, B_3, B_5 and B_7 standards (D). Two biological replicates (R1 and R2) of extracts from spent media of *C. reinhardtii* monocultures (Cre) and *C. reinhardtii* and *M. lacteus* cocultures (Cre+Mla) were used for methionine (B and C, respectively) as well as for the B vitamins (E and F, respectively). The detection wavelength for methionine was 260 nm, and the detection limit 0.001 mg/mL. The detection wavelength for the B vitamins was 220 nm, and the detection limit 0.002 mg/mL.

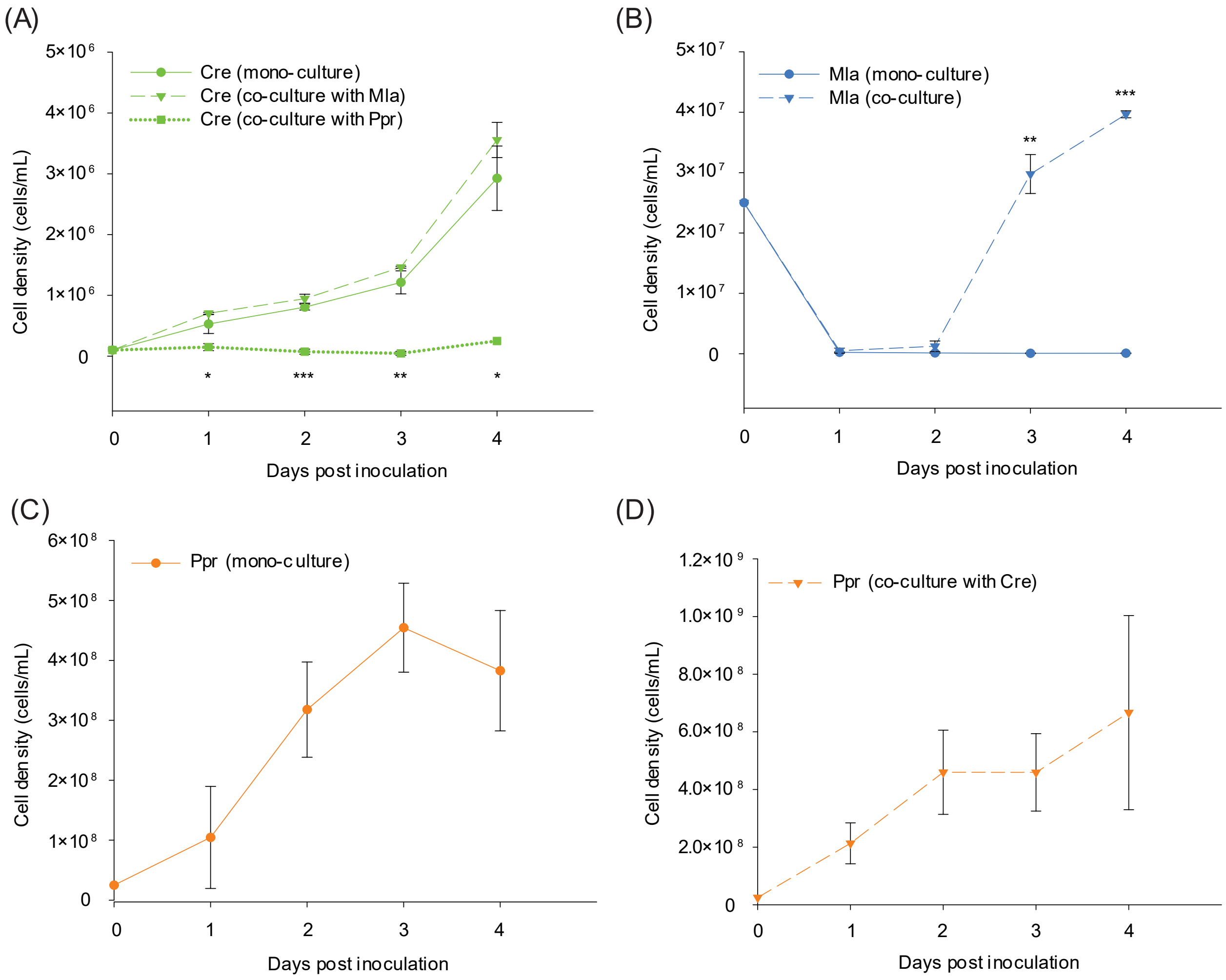

Fig. S5: Growth of *C. reinhardtii* (Cre), *P. protegens* (Ppr) and *M. lacteus* (Mla) in mono- and bipartite cultures under low phosphate conditions. Algal and bacterial cells were grown in TAP medium in the presence of 0.2 (w/v) glucose and 0.1 mM phosphate (*SI Appendix*., Methods). In cocultures, the two organisms were inoculated at a ratio of 1:250 (algae:bacteria) and grown for four days. Bacterial cell densities were calculated by serially diluting and plating on LB medium daily. Colonies were counted after two days of growth, and cell densities were determined considering the dilution factor. Asterisks indicate significant differences between mono- and cocultures as calculated by Student's t-test (*: $P \leqslant 0.05$; **: $P \leqslant 0.01$; and ***: $P \leqslant 0.001$). The error bars indicate SDs with n = 3 biological replicates per time point and culture.

Replicate 1

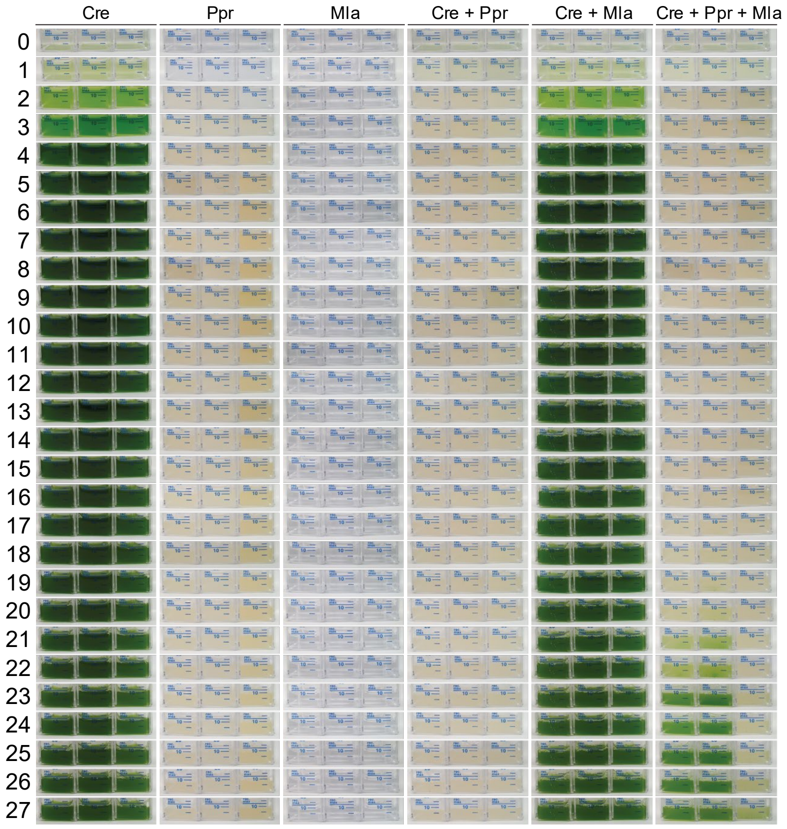

Replicate 2

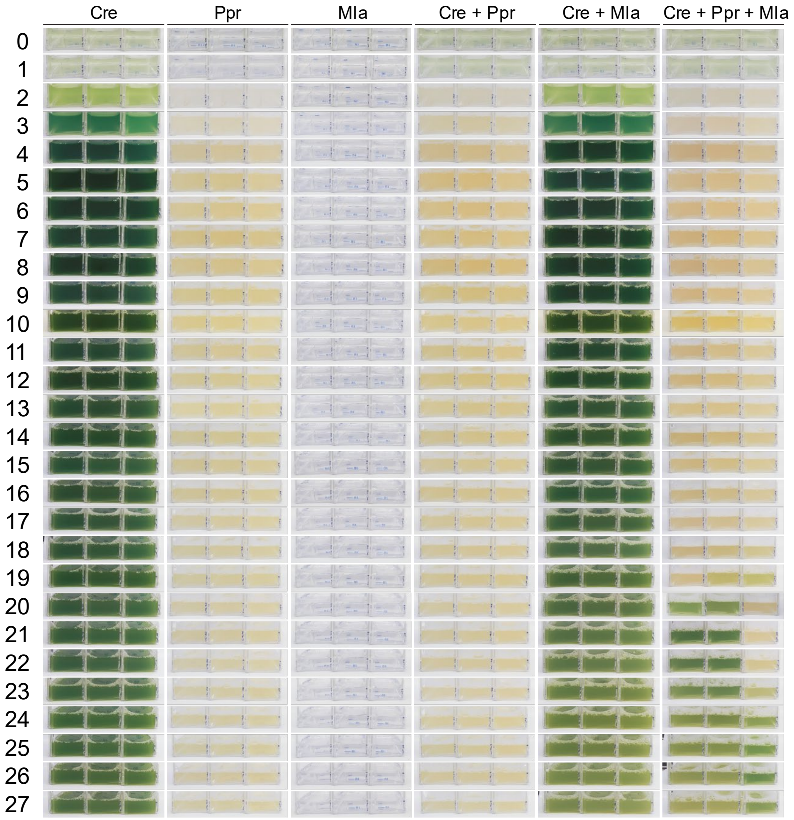

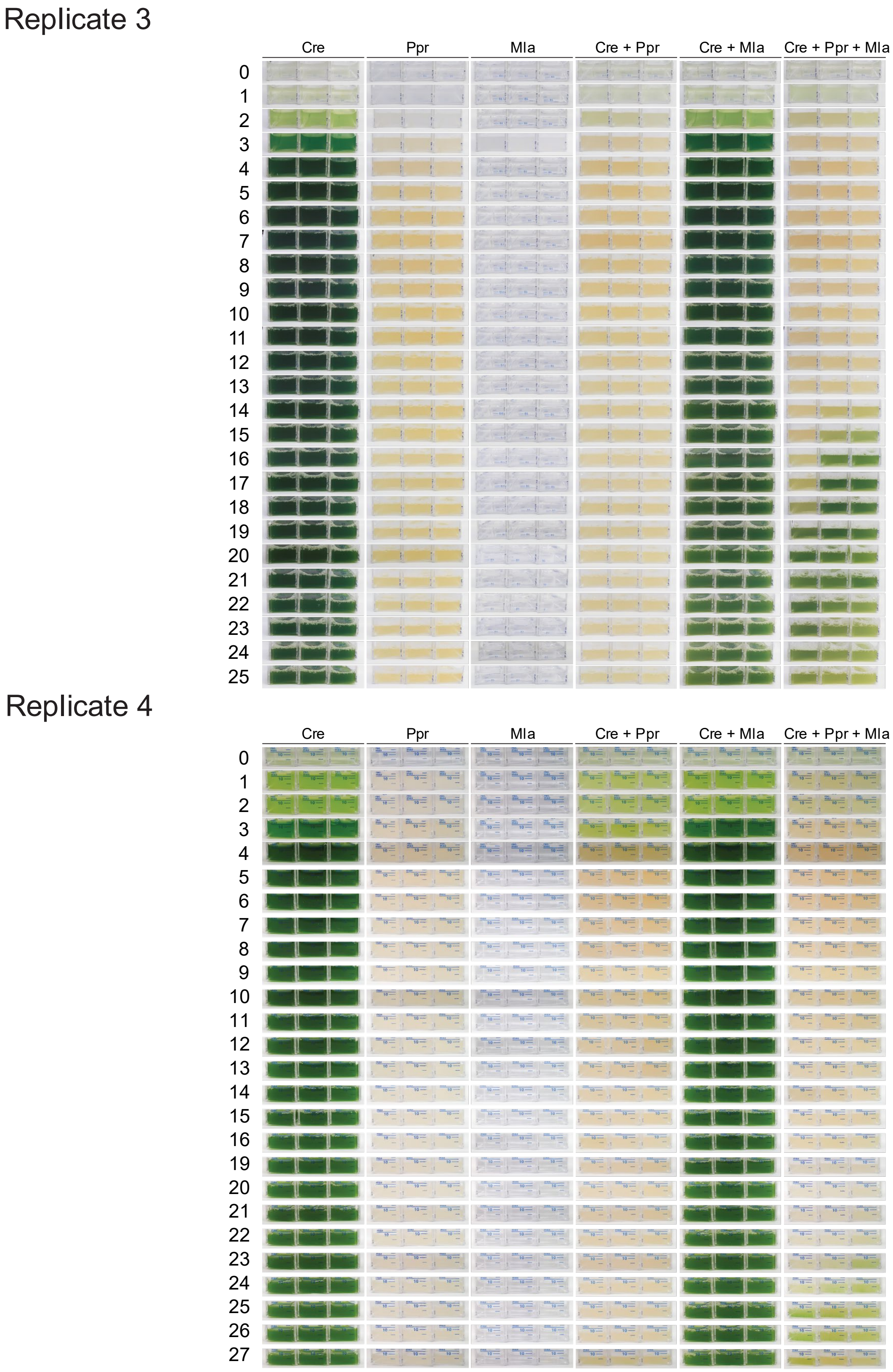

Fig. S6: *C. reinhardtii* recovers from the antagonistic bacterium *P. protegens* with aid of the helper bacterium *M. lacteus*.

Algal and bacterial monocultures as well as bi- and tripartite cocultures. The organisms were cocultivated in TAP medium containing 0.1 mM phosphate supplemented with 0.2% (w/v) glucose at a ratio of 1:250:250 (Cre:Ppr:Mla). Equally treated cultures of *C. reinhardtii* (Cre), *P. protegens* (Ppr) and *M. lacteus* (Mla) served as reference for the growth of the axenic cultures. The full first replicate from Fig. 2*A* and three additional independent biological replicates are shown.

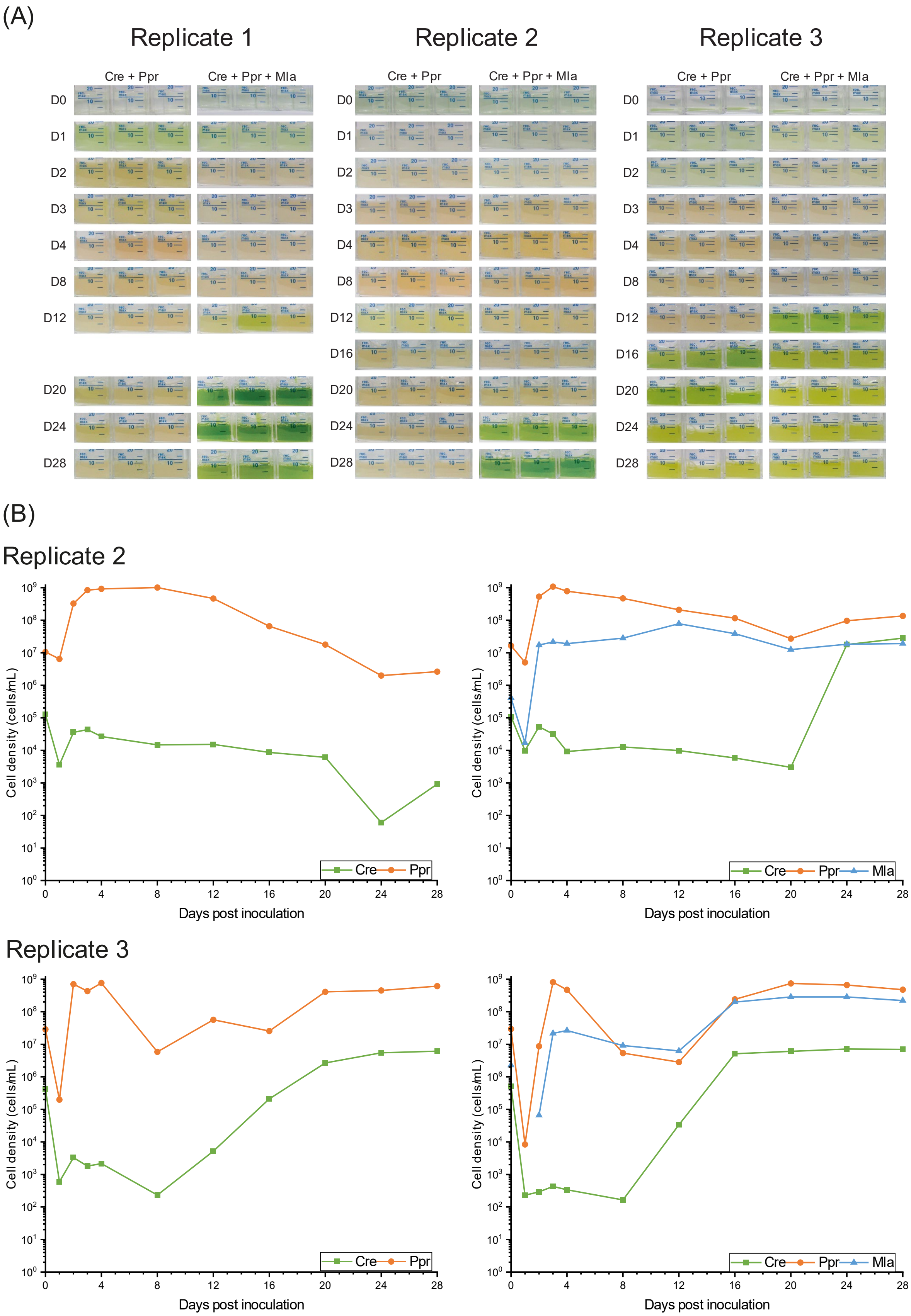
(A)
Replicate 1
Replicate 2
Replicate 3
Cre + Ppr
Cre + Ppr + Mla
D0
D1
D2
D3
D4
D8
D12
D16
D20
D24
D28
(B)
Replicate 2
Replicate 3
Cell density (cells/mL)
Days post inoculation
Cre
Ppr
Mla

(C)

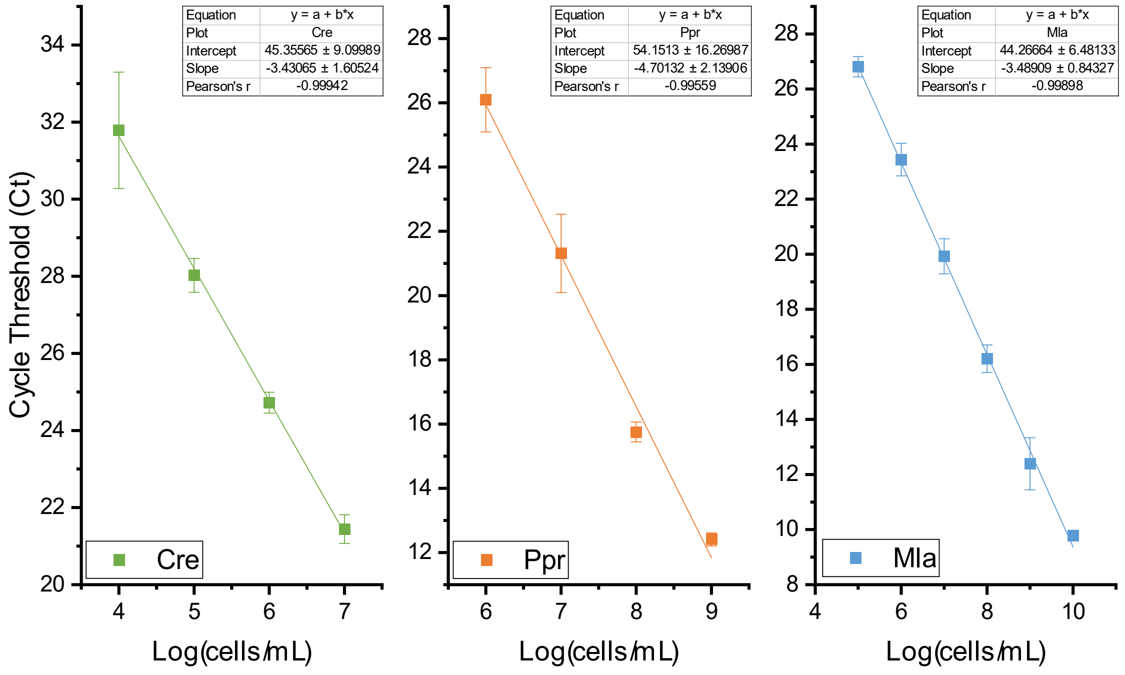

Fig. S7: Algal and bacterial cultures and cell densities of the bi- and tripartite cultures

(A): Photodocumentation of algal and bacterial monocultures as well as bi- and tripartite cocultures used for the qPCR determinations. Growth conditions are detailed in the legend of Fig. S6. *C. reinhardtii* (Cre), *P. protegens* (Ppr), *M. lacteus* (Mla). In one case (day 16 of replicate 1), the documentation is missing due to technical problems.

(B): Cells from 10 mL cell suspension of the cultures shown in (B, replicates 2 and 3) were harvested on days 0-4 post inoculation and then every fourth day. Genomic DNA was extracted and used for qPCR to determine cell densities (see methods for details). Replicate 1 is shown in Fig. 2*B* and *C*.

(C): Calibration curves for algal and bacterial cell densities based on genomic DNA quantification. The cell densities calculated based on cell counts, for *C. reinhardtii* (Cre), or OD_{600} values, for *P. protegens* (Ppr) and *M. lacteus* (Mla) were correlated to the cycle threshold (Ct) of the qPCR.

Replicate 2

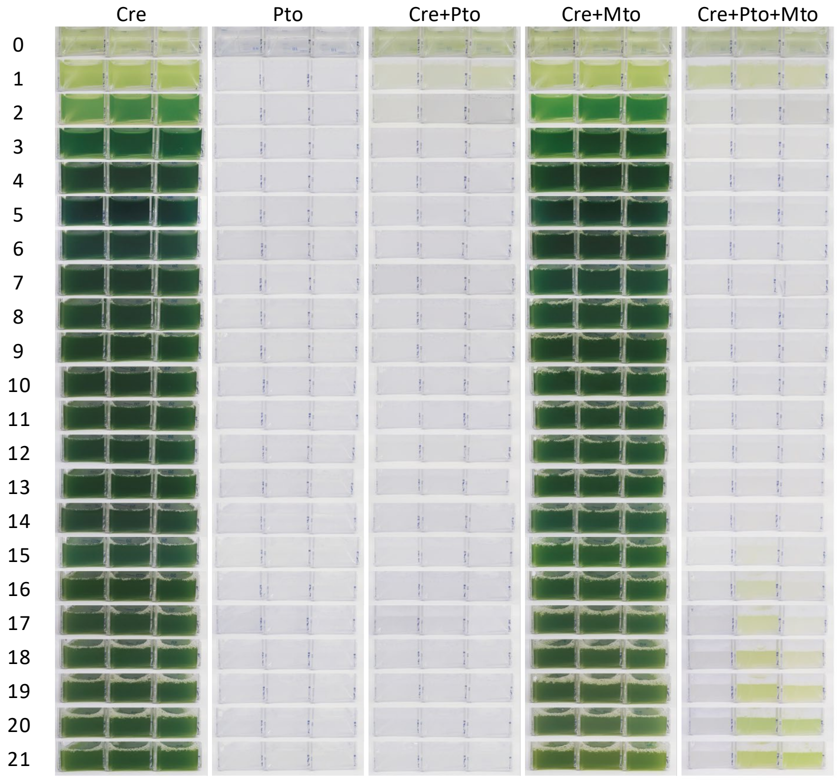

Replicate 3

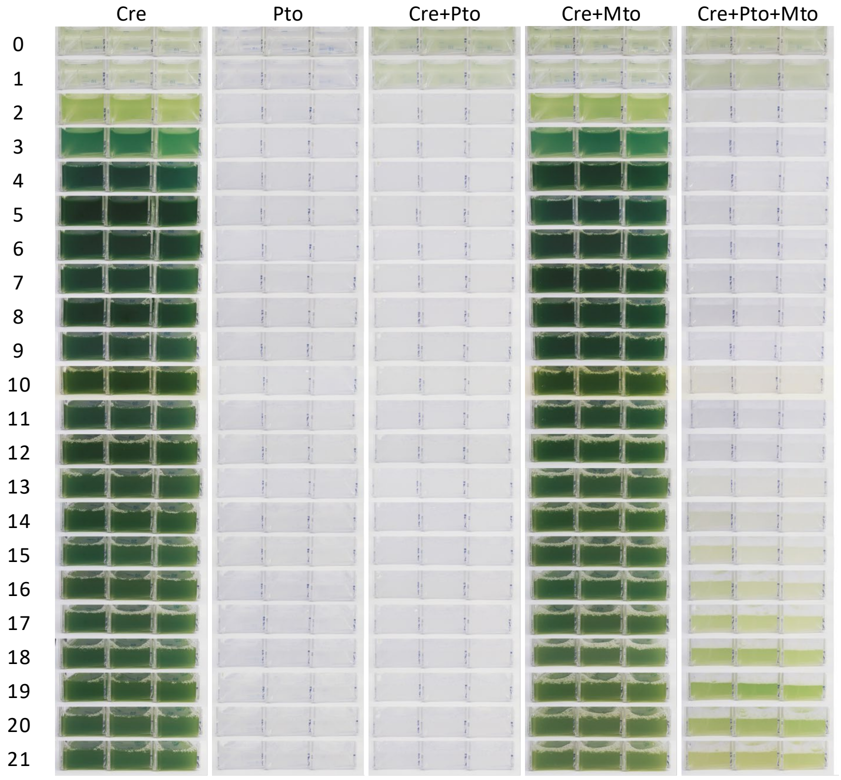

Fig. S8: *C. reinhardtii* recovers from the antagonistic bacterium *P. tolaasii* with the aid of the helper bacterium *M. tolaasinivorans*.

Photodocumentation of algal and bacterial monocultures as well as bi- and tripartite cocultures. *C. reinhardtii* (Cre), *P. tolaasii* (Pto), *M. tolaasinivorans* (Mto). See Fig. 2*D* for details.

Replicate 2

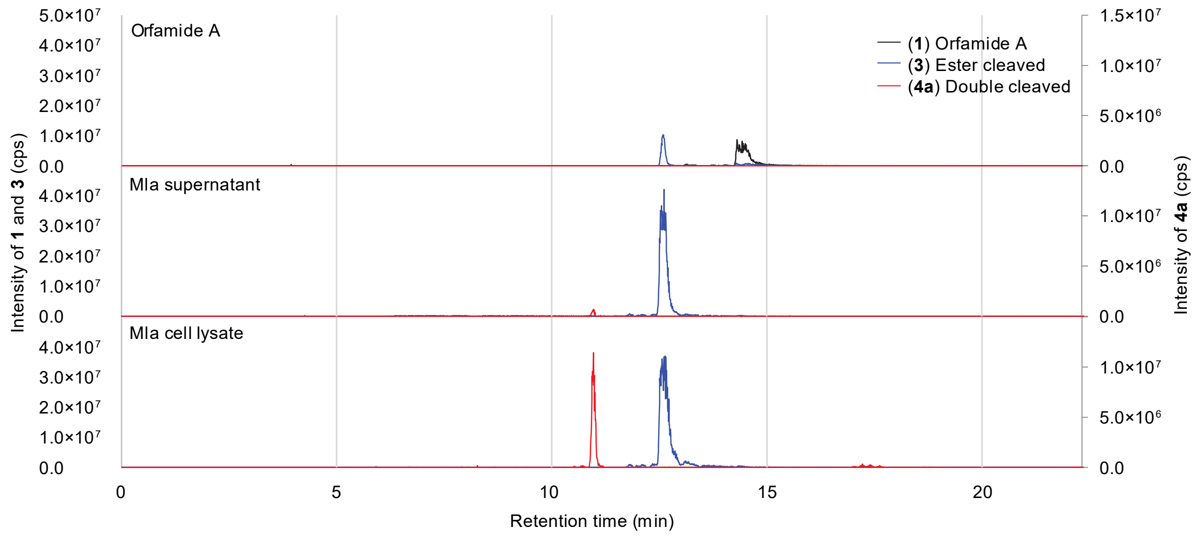

Fig. S9: LC-HRMS analysis of a second biological replicate showing the degradation of intact orfamide A (*m/z* 1295.84 [*M* + H]$^+$).

Orfamide A is cleaved to either orfamide A with one cleavage (*m/z* 1313.85 [*M* + H]$^+$) or orfamide A with two cleavages missing the *C*-terminal residues Leu-Ser-Val (*m/z* 1014.66 [*M* + H]$^+$) after 24 h incubation with the cell lysate of *M. lacteus*.

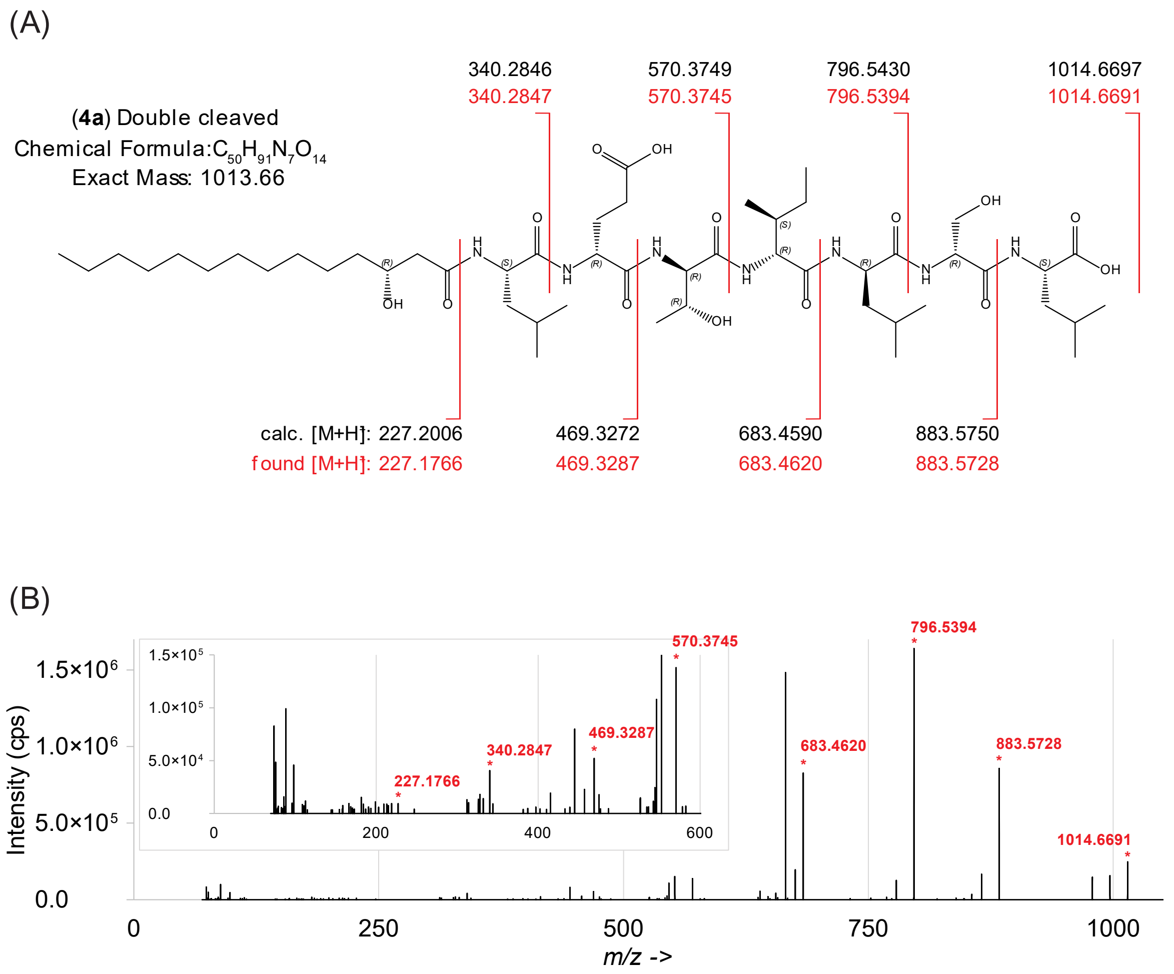

Fig. S10: Evidence of the double-cleaved orfamide A degradation product **4a** (*m/z* 1014.66 [*M* + H]$^+$).

(A) Theoretical α-fragmentation of the compound **4a**, showing the calculated ions (black) and the found ions using LC-HRMSMS (red)

(B) Average of five consecutive MS/MS chromatograms from the precursor ion with *m/z* 1014.66 [*M* + H]$^+$ showing the α- fragments detected (red stars). For the chromatogram with the highest abundance, the experimental m/z have been indicated. Inlets show an amplification of the region 0 to 600 (*m/z*).

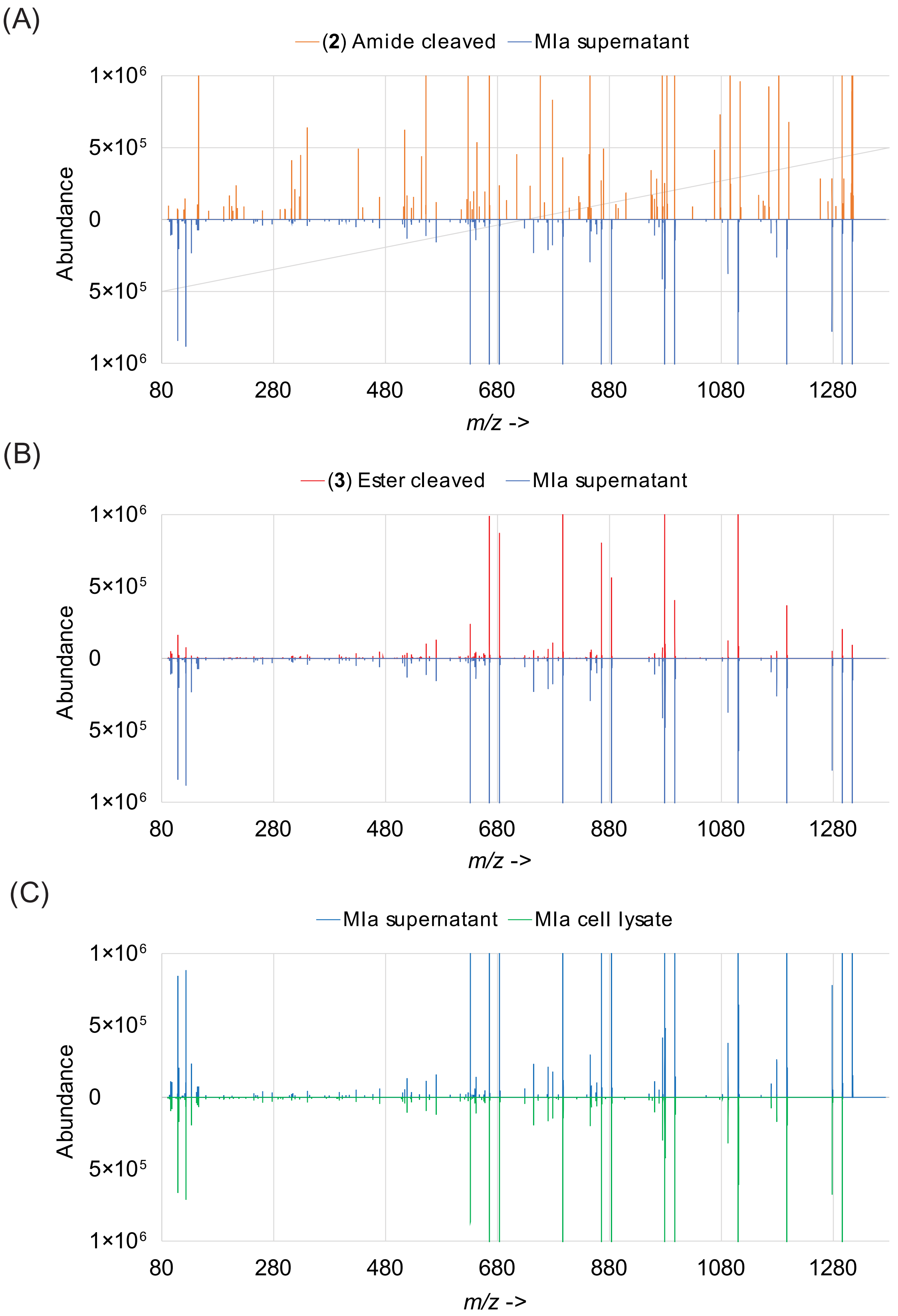
(A)
(2) Amide cleaved
MIa supernatant
Abundance
1×10^6
5×10^5
0
5×10^5
1×10^6
80
280
480
680
880
1080
1280
m/z ->
(B)
(3) Ester cleaved
MIa supernatant
Abundance
1×10^6
5×10^5
0
5×10^5
1×10^6
80
280
480
680
880
1080
1280
m/z ->
(C)
MIa supernatant
MIa cell lysate
Abundance
1×10^6
5×10^5
0
5×10^5
1×10^6
80
280
480
680
880
1080
1280
m/z ->

Fig. S11: MS/MS patterns of both ester (**3**) and amide cleaved (**2**) compounds compared with the double-cleaved **4a** (*m/z* 1313.85 $[M + H]^+$) peak from the degradation assay

(A) Comparison of the fragmentation pattern from the synthetic compound representing the cleavage of the amide bond with the spent supernatant

(B) Comparison of the fragmentation pattern from the synthetic compound representing the cleavage of the ester bond with the spent supernatant

(C) Comparison of the fragmentation pattern from the spent supernatant and the cell lysate

Replicate 2

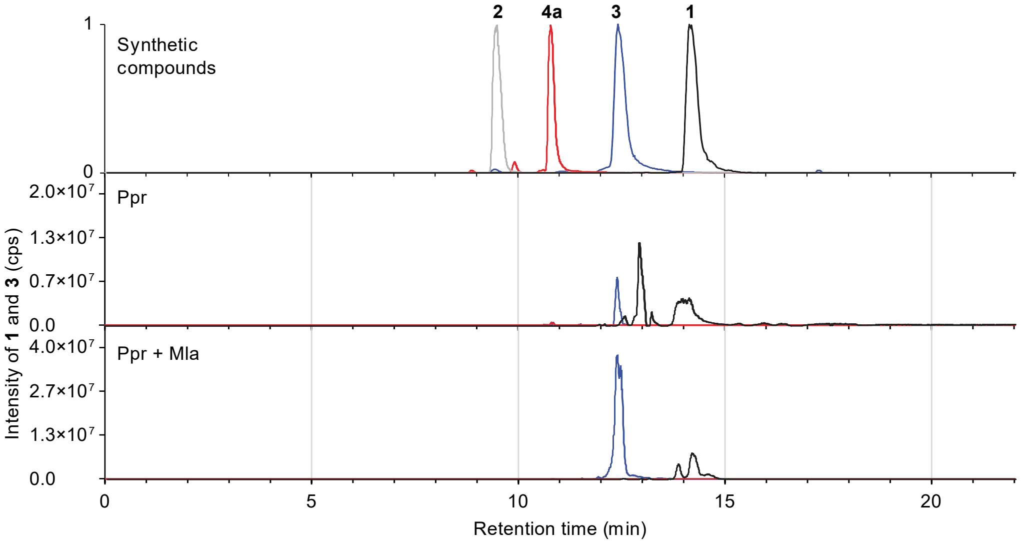

Fig. S12: Coculture of *P. protegens* and *M. lacteus* with the ester cleaved form of orfamide A. Synthetic compounds (**1**, orfamide A; **2**, amide cleaved; **3**, ester cleaved; **4a**, double-cleaved) are used for comparison (Fig. 3*C*).

Replicate 1

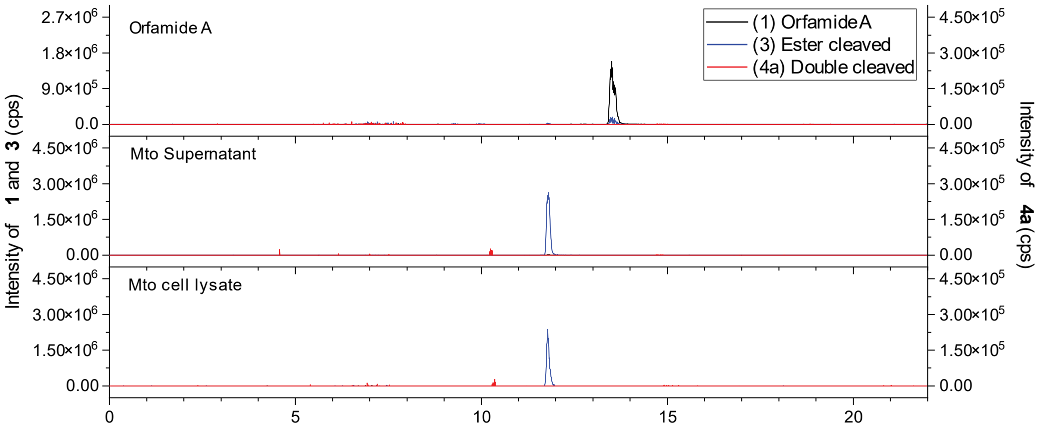

Replicate 2

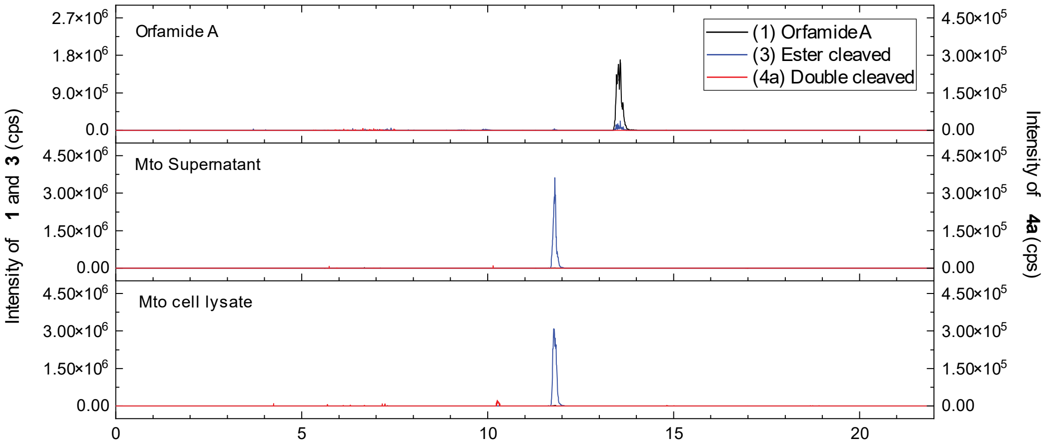

Fig. S13: LC-HRMS analysis showing that *M. tolaasinivorans* is able to cleave orfamide A in a single cleaved product.

(A)

Replicate 1

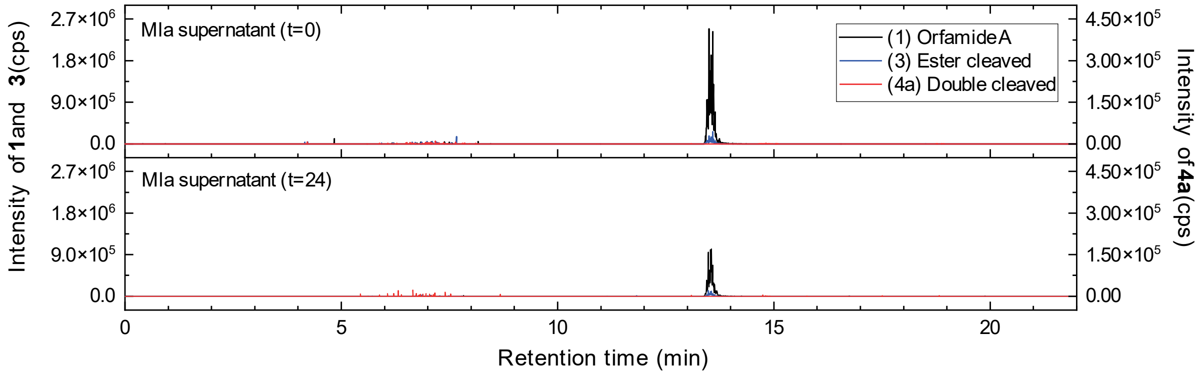

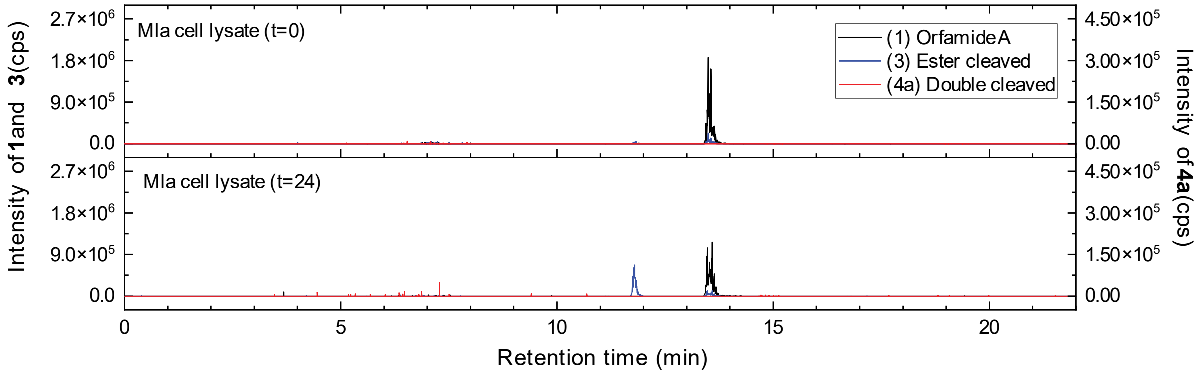

Replicate 2

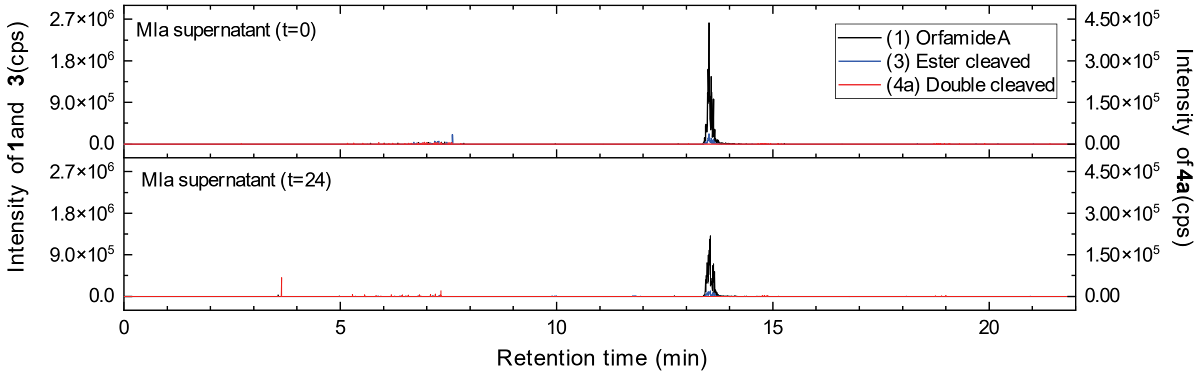

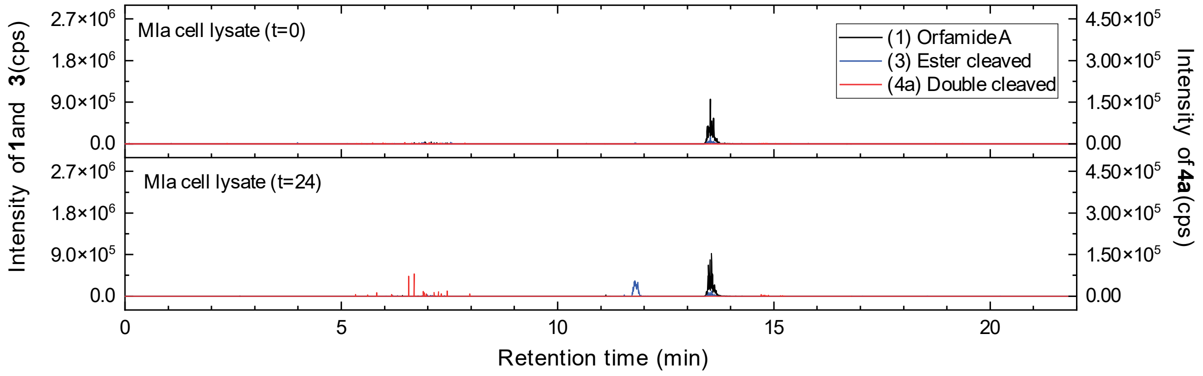

(B)

Replicate 1

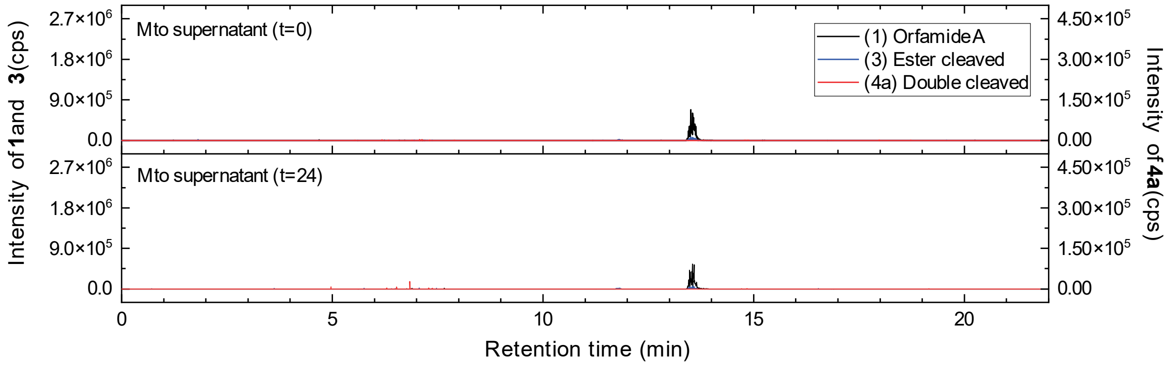

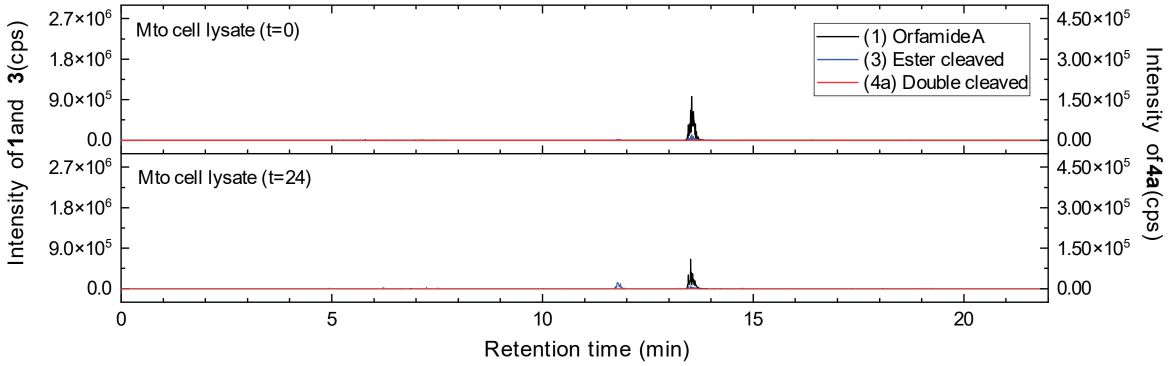

Replicate 2

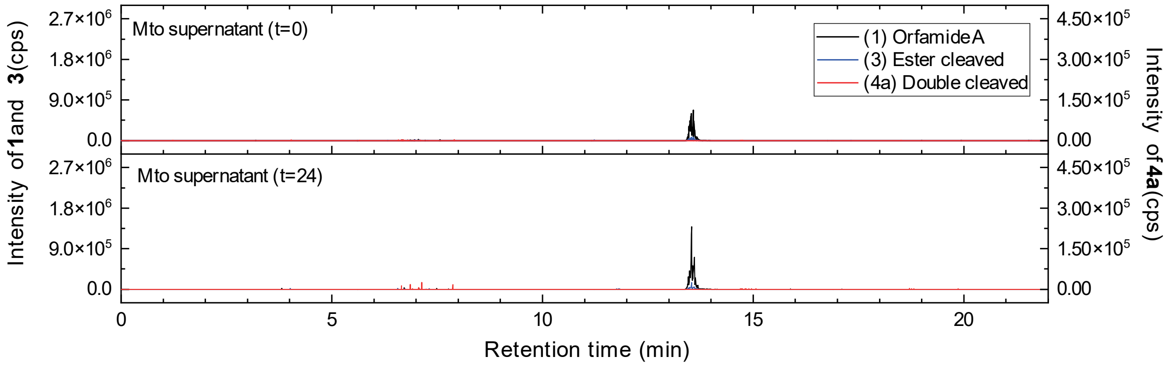

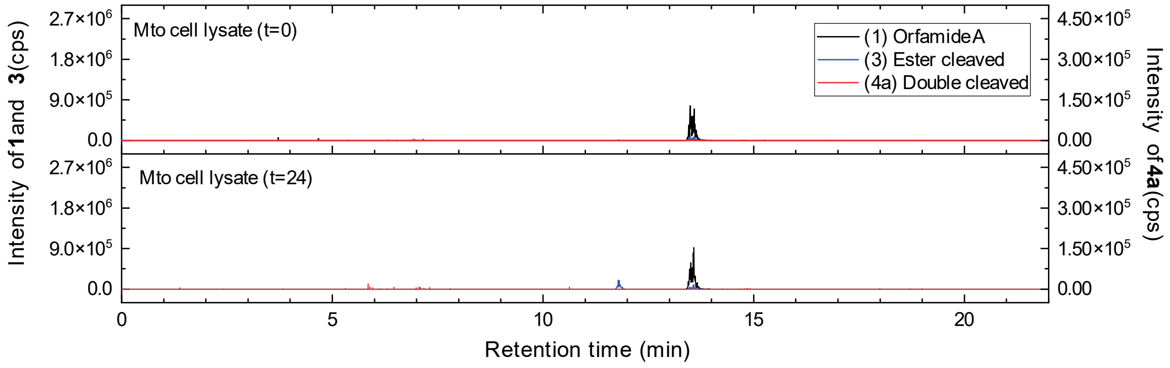

Fig. S14: Heat treatment of the supernatants and cell lysates of *M. lacteus* (Mla, A) and *M. tolaasinivorans* (Mto, B), respectively, prevents the cleavage of orfamide A fully (supernatant) or to a large extent (cell lysate Mla).

LC-HRMS profiles of the potential degradation of intact orfamide A (*m/z* 1295.84 $[M + H]^+$; **1**) to **3** (*m/z* 1313.85 $[M + H]^+$;) or **4a** (*m/z* 1014.66 $[M + H]^+$) after an incubation time of 24 h (t = 24) with heat-treated spent media (supernatant) of the bacterial overnight culture in LB, and heat-treated cell lysate, respectively (see *SI* Appendix, Methods for details). Samples with orfamide A added to heat-treated supernatants and to heat-treated cell lysates, respectively, that were not further incubated (t = 0) were used as controls. Two independent biological replicates (labeled as replicate 1 and 2) are shown for each case.

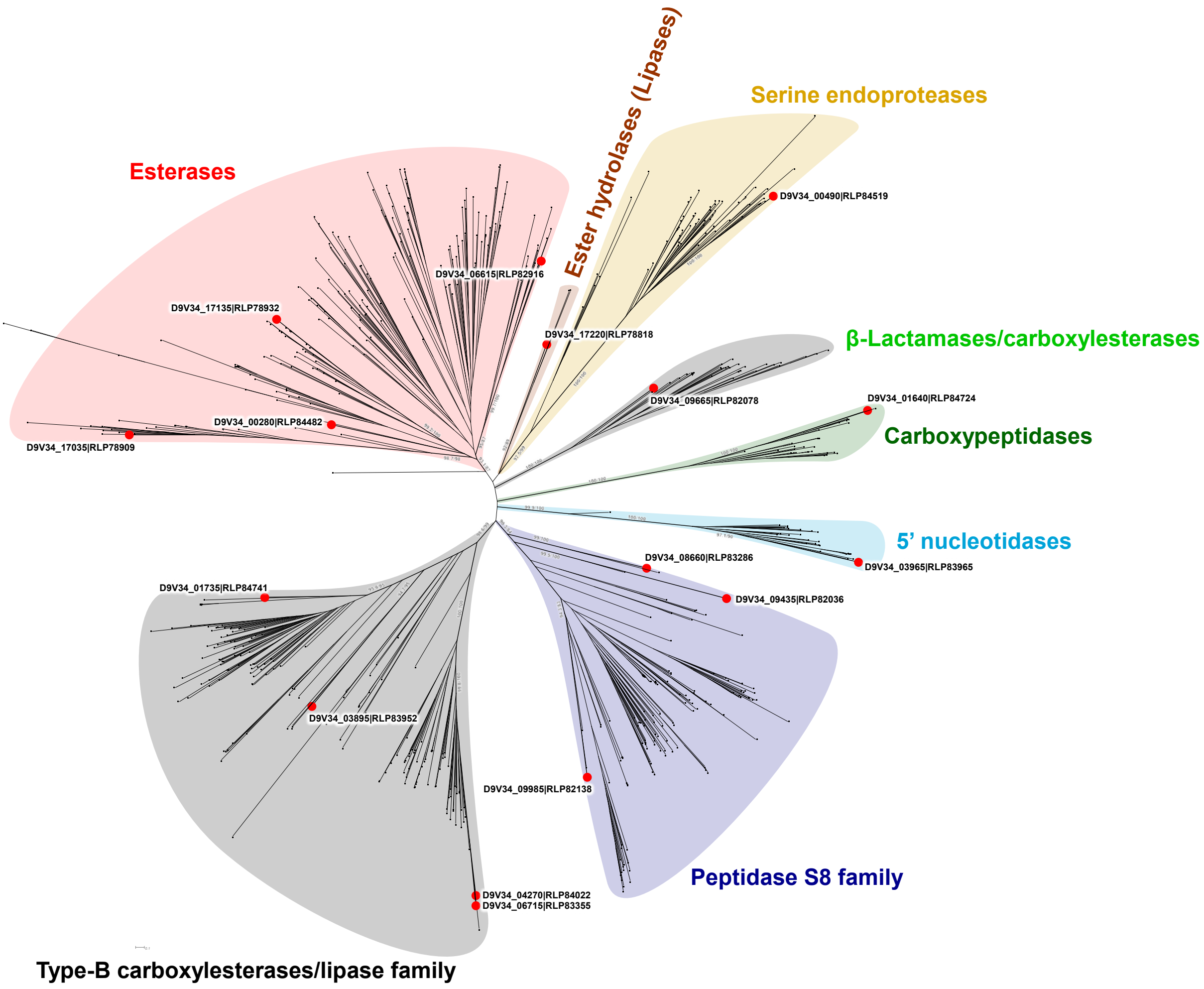

Fig. S15: Phylogenetic tree of distinct hydrolase families including those (red nodes) of *M. lacteus*

The analysis encompasses reference esterases and peptidases that may cleave orfamide A in a peptide bond or in the ester bond. The tree was built using sequences obtained from the uniprot database and the *M. lacteus* genome (*SI Appendix*, Methods). Used sequences are listed in Table S3. The different families are shown. The small group of 5'-nucleotidases appeared after homology enrichment due to a high homology with other esterases. The scale bar (0.1) is indicated, showing the evolutionary distance (expected substitutions per site). The values next to the nodes represent SH-aLRT (SH-like approximate likelihood ratio test) support (%)/UFBoot (ultrafast bootstrap) support (%). Red nodes correspond to the *M. lacteus* candidate enzymes.

Table S1.

Presence of methionine and B vitamins in a monoculture of *C. reinhardtii* (Cre) and in coculture of *C. reinhardtii* and *M. lacteus* (Cre + Mla)

Biomolecule	Concentration (ng/million algal cells)			
	Replicate 1		Replicate 2	
	Cre	Cre + Mla	Cre	Cre + Mla
Methionine	12.18	8.50	18.35	8.65
Vitamin B_1	2.98	1.52	2.71	1.10
Vitamin B_3	1.66	0.45	1.79	0.77
Vitamin B_5	1.14	0.62	1.61	0.68
Vitamin B_7	Non-detected (n.d.)	n.d.	n.d.	n.d.

The amounts for each biomolecule were determined on day five of growth and are indicated in ng per million algal cells. Two biological replicates were performed.

Material and methods (containing Fig. S16-S22 and Table S2)

Strains and culture conditions

Experiments were carried out with *C. reinhardtii* wild-type strain SAG 73.72 (mt^+), also known as CC-3348. It was obtained from the Culture Collection of Algae at Göttingen University (SAG). Strain 73.72 derives from the first isolate of *C. reinhardtii* (Smith 1945 isolated from a potato field near MA, USA) that was passed over to Sager (1953, known as strain 21 gr) and to Tsubo (1954, known as strain C8) (1). SAG73.72 is the direct derivative of C8 (https://sagdb.uni-goettingen.de/detailedList.php?str_number=73.72). The algal cells were grown in TAP medium (1) in the presence of 0.2% (w/v) glucose unless otherwise stated. In some cases, the phosphate concentration was lowered to 0.1 mM phosphate, as indicated. In some other cases, algal cells were grown in TP medium lacking acetate.

The bacterium *P. protegens* Pf-5 BAA-477™ (2) was obtained from the American Type Culture Collection (ATCC). It was isolated from the root surface of cotton (https://www.atcc.org/products/baa-477). The helper bacteria *M. lacteus* DSM15177 and *M. tolaasinivorans* DSM15179 (3) were obtained from the German Collection of Microorganisms and Cell Cultures GmbH (DSMZ, https://www.dsmz.de/) in Braunschweig, Germany. They were isolated from the cultivated mushroom *Pleurotus ostreatus*. *P. tolaasii* DSM19342 was obtained by the Jena Microbial Resource Collection (JMRC). It was isolated from the fungus *Agaricus bisporus* and dates originally also from DSMZ. Axenic bacteria were grown in Luria-Bertani (LB) medium at 28 °C with constant orbital shaking (200 rpm) or in TAP medium with 0.2% (w/v) glucose, as stated.

Correlation between OD_{600} and bacterial cell density of *P. protegens* and *M. lacteus*

Bacterial cultures were grown in LB medium overnight to an $OD_{600} > 2$ (*M. lacteus*) and $OD_{600} > 4$ (*P. protegens*) as described above. OD_{600} was determined with a Jenway 6305 spectrophotometer using diluted samples of bacterial cell suspensions. A series of dilutions was prepared in TAP medium and kept on ice. From each dilution, 100 µL were transferred to an Acuvette ST (Beckman Coulter, No. A35473) containing 10 mL of BCI Isoton II electrolyte (Beckman Coulter, No. 8448011). After gently mixing by inversion, the sample was analyzed by a Beckman Coulter Multisizer 4e Coulter Counter, with an aperture of 30 µm. The current was set to 600 µA, preamp gain to 4, and the analytic volume to 50 µL. Due to the limitation

to detect particles smaller than 0.6 µm, the data were fitted to a gaussian function and integrated using OriginPro (Version 2018, OriginLab Corporation, Northampton, MA, USA). The cell densities measured for each dilution were then correlated to their respective OD_{600} measurements and the resulting calibration curve was used to calculate the bacterial cell densities in all experiments where OD_{600} was measured. The dilutions were made with two biological replicates for *M. lacteus* and with three biological replicates for *P. protegens*, and each sample was measured with at least three technical replicates (Fig. S16). This method was used for the adjustment of bacterial cell densities unless otherwise stated.

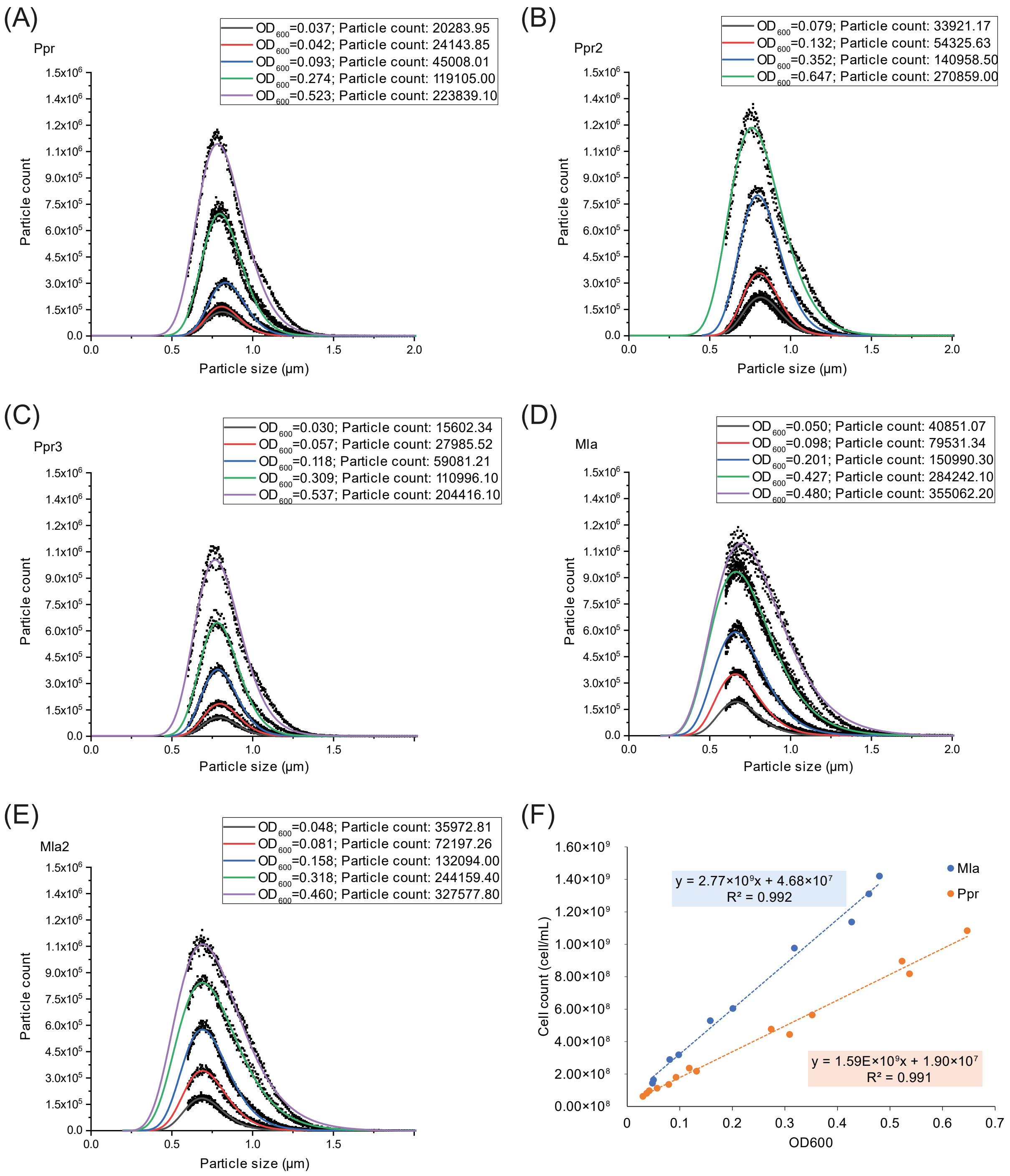

Fig. S16: Correlation of OD_{600} values to bacterial cell densities

(A-C) Cell size distribution of an overnight culture of *P. protegens* (Ppr) diluted to different OD_{600} values. The cell size distribution was fitted to a gaussian function, and its integral was used to calculate the cell density in each dilution.

(D-E) Distribution of cell sizes in an overnight culture of *M. lacteus* (Mla) diluted to different OD_{600} values. The measured cell size distribution was fitted to a gaussian function and its integral was used to calculate the cell density of each dilution.

(F) Linear regression of bacterial cell density over OD_{600} values of *P. protegens* or *M. lacteus* dilutions.

Correlation between OD_{600} and bacterial cell density of *P. tolaasii* and *M. tolaasinivorans*

Bacterial cultures were grown in LB medium overnight to an $OD_{600} > 2$ (*M. tolaasinivorans*) and $OD_{600} > 4$ (*P. tolaasii*) as described above for *M. lacteus* and *P. protegens*. OD_{600} was determined with a Jenway 6305 spectrophotometer using diluted samples of bacterial cell suspensions. A series of dilutions were prepared in TAP medium and kept on ice. From each dilution, either 20 µL, 100 µL or 200 µL were transferred to an LB plate and incubated for 24 h or 48 h until colonies were properly grown and visible for counting. The CFU counts were then used together with the OD_{600} to make a calibration curve (Fig. S17).

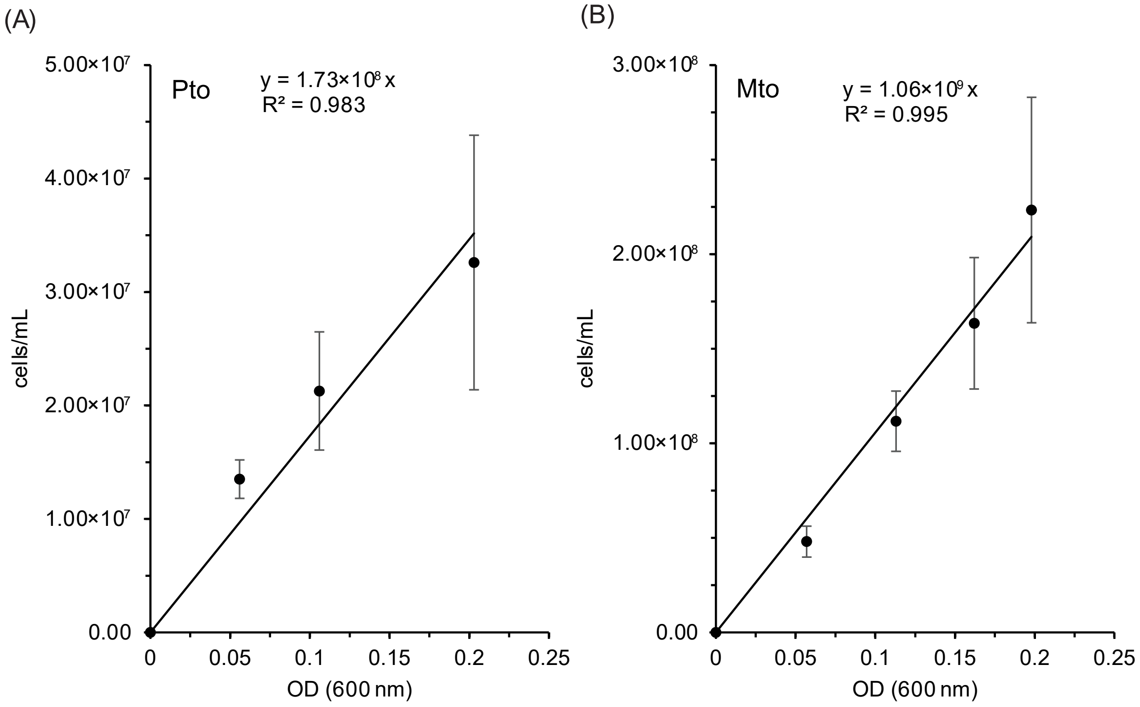

Fig. S17: Correlation of OD_{600} values to bacterial cell densities of *P. tolaasii* (Pto, A) and *M. tolaasinivorans* (Mto, B)

Algal and bacterial mono- and cocultures

For the mono- and cocultures, *C. reinhardtii* wild-type cells were precultured to a cell density of 3–6 × 10^6 cells mL^{-1}. *C. reinhardtii* was grown in liquid TAP medium in the presence of 0.2% (w/v) glucose and with 0.1 mM phosphate when indicated at 23 °C under a 12:12 light–dark cycle under white light (Osram L36W/840, lumilux, cool white, Osram) with a light intensity of about 60 µmol m^{-2} s^{-1} and orbital shaking (100 rpm). Bacterial precultures were grown in LB medium overnight to an OD_{600} > 2 (for *M. lacteus* and *M. tolaasinivorans*) and an OD_{600} > 4 (for *P. protegens* and *P. tolaasii*), as determined by OD_{600} measurements of diluted cultures. OD values were correlated with bacterial cell densities (Fig. S16 and method above; Fig. S17). Prior to cocultivation, bacterial cells were washed twice with TAP medium. Cells were then resuspended in TAP medium supplemented with 0.2% glucose and 0.1 mM phosphate as indicated, at a starting algal cell density of 1 × 10^5 cells mL^{-1} and a bacterial cell density of 2.5 × 10^7 cells mL^{-1}, giving a final ratio of 1:250 algal cells to bacteria in bipartite cultures. In tripartite cultures, a final ratio of 1:250:250 algal cells to bacterium 1 (for *P. protegens* and *P. tolaasii*) to bacterium 2 (for *M. lacteus* and *M. tolaasinivorans*) was applied. These cultures were grown under the conditions mentioned above for the growth of *C. reinhardtii*. The cultures were photodocumented daily.

Cell quantifications of *M. lacteus* and *P. protegens via* plating

Cell suspensions were serially diluted, plated on LB plates, and grown at 28 °C for two days. Colonies were counted and cell densities were calculated considering the dilution factor.

C. reinhardtii cell quantification *via* counting

Algal cell density was determined with a Thoma cell counting chamber.

Genomic DNA extraction for qPCR

Samples were harvested by centrifugation at 3435 x *g* for 20 min. The supernatant was discarded, and the cell pellet was stored at -20 °C until extraction. For DNA extraction, the protocol from Pollock (4) was adjusted as follows: The extraction buffer consisted of 100 mM Tris, 10 mM Na_2EDTA, 10x Yellow Sample Buffer; pH was adjusted to 8. Yellow Sample Buffer 40x was obtained from ThermoFisher Scientific (No. R1381). An aliquot of 200 µl of

extraction buffer was added to the frozen cell pellets. The pellets were thawed at room temperature for 30-40 min, thoroughly vortexed to full resuspension and incubated in a water bath at 100 °C for 10 min. The mixture was briefly vortexed and centrifuged at 3435 x *g*, 4 °C for 20 min. The supernatant was transferred to new vials and stored at -20 °C for further use and the remaining cell debris was discarded.

DNA-based cell quantification *via* qPCR

Cell densities were determined by correlating the quantified DNA to its equivalent cell density using calibration curves for each microbial culture. Algal cultures were inoculated at a cell density of 4–6 × 10^6 cells · mL^{-1} and incubated for 3 to 4 days. The cultures were then counted with a Thoma cell counting chamber and adjusted to 10^7 cells · mL^{-1} in 50 mL TAP. The suspension was then serially diluted in TAP, resulting in cell densities of 10^7, 10^6, 10^5, and 10^4 cells mL^{-1}. Overnight cultures of the bacteria were adjusted to 10^{10} cells · mL^{-1} in 50 mL TAP using the correlation between OD_{600} and cell density. The suspension was then serially diluted in TAP, resulting in cell densities of 10^9, 10^8, 10^7, 10^6, 10^5, and 10^4 cells mL^{-1}. The gDNA from the dilutions was then extracted as described above and quantified as for the samples. The resulting calibration curves were used to correlate the Ct to cell densities (Fig. S7*C*).

Quantitation of gDNA *via* qPCR was performed using the Luna Universal qPCR Master Mix (New England Biolabs) on an AriaMx Real-time PCR System (Agilent) by following the manufacturer's protocol. No template control and no amplification control were included in each run. After baseline correction, the Ct values of the samples were obtained. The following organism-specific primers were used that do not cross-amplify any of the other two organisms: CCCCAGTCAACGTTTGCTTA and CAAAGTGTAGCGCTAGGGAC for *C. reinhardtii*, for *P. protegens* CCCCCTATAGAAGCGTAGACCG and CTCTTTCTGTTGTTATTGGCTGTG, and ATAATGGCGATCACGAGTGCTG and TTGGAATCTTCT TTTGGCGTCT for *M. lacteus*. The extraction of the gDNAs for a cell concentration of 10^6 cells mL^{-1} for *C. reinhardtii*, for either 10^8 cells mL^{-1} or 10^9 cells mL^{-1} for *M. lacteus* and either 10^8 cells mL^{-1} or 10^9 cells mL^{-1} for *P. protegens* were used as internal controls in all qPCR rounds.

M. lacteus auxotrophy test for B vitamins and its need for organic sulfur

M. lacteus from an overnight culture that was grown in LB medium was washed twice and resuspended in TAP medium with 0.2% (w/v) glucose. All cultures were inoculated with the bacteria to a final cell density of 2×10^5 cells mL^{-1} and incubated at 28 °C, under shaking conditions for five days.

To test if *M. lacteus* needs any amino acids, TAP medium was supplemented with 0.2% (w/v) glucose and a mixture of nine B vitamins (B_1, B_2, B_3, B_5, B_6, B_7, B_8, B_{10}, B_{12}) at a final concentration of 1 mg L^{-1}. Each culture was supplemented with one of the 20 proteinogenic amino acids (L-Aspartic acid, L-Tryptophan, L-Asparagine·H_2O, L-Cysteine, L-Glutamic acid, L-Glutamine, L-Leucine, L-Methionine, L-Tyrosine, L-Isoleucine, L-Phenylalanine, L-Alanine, L-Arginine, Glycine, L-Lysine·2HCl, L-Proline, L-Serine, L-Threonine, L-Valine and L-Histidine·HCl·H_2O) at a final concentration of 1 g L^{-1}. Finally, the pH was adjusted to 7.0 with 1 M NaOH. As a negative control, TAP plus 0.2% glucose was used. As a positive control, TAP plus 0.2% glucose with 20 amino acids and the nine above-mentioned B vitamins was used, and was used as the reference to calculate the final relative growth. To determine bacterial cell growth, OD_{600} values were measured after five days of culturing, when maximal cell densities were reached.

To test if *M. lacteus* is auxotrophic for any B vitamins, TAP medium was supplemented with 0.2% (w/v) glucose and 1 g mL^{-1} of methionine. Each culture was supplemented with eight B vitamins out of nine, one of the vitamins was missing. The final concentration of each vitamin was 1 mg L^{-1}. Finally, the pH was adjusted to 7.0 with 1 M NaOH. As a negative control, TAP medium plus 0.2% glucose was used. As a positive control, TAP with 0.2% glucose with 1 mg L^{-1} methionine and nine B vitamins was used.

The positive controls were used as references to calculate the relative growth amounts.

To test the possible usage of other sulfur sources by *M. lacteus*, different reduced and organic sulfur sources were assayed in TAP medium supplemented with 0.2% (w/v) glucose and B vitamins (B_1, B_3, B_5, B_7) at a final concentration of 1 mg L^{-1}. For that, a stock solution of 100 mM of each sulfur compound (β-mercaptoethanol, dithiothreitol, glutathione, lipoic acid and dimethyl sulfoxide) was prepared in ddH_2O. For lipoic acid, the solid powder was solubilized in a small amount of ethanol and brought to the right concentration using ddH_2O. The final concentration assayed for the different sulfur sources was 1 mM. As positive control, 1 g L^{-1} of methionine was used.

M. lacteus auxotrophy test for glucose in mono- and coculture

The *M. lacteus* mono- and cocultures with *C. reinhardtii* cells were prepared and cultivated following the conditions as mentioned above ("Algal and bacterial mono- and cocultures"). They were cocultivated for 5 days in TAP medium lacking acetate, called TP medium.

Extraction of free amino acids from *C. reinhardtii*

The experiments were performed according to Araya et al. (5) with some modifications. The axenic culture of *C. reinhardtii* was inoculated with a starting concentration of 1×10^5 cells mL^{-1}. The culture was grown at 23 °C, 12:12 hour light-dark cycle, under shaking conditions for 5 days prior to extraction. 500 mL of supernatant from the 5-day old *C. reinhardtii* culture was collected and evaporated at 50 °C. After drying completely, 600 µL of H_2O: $CHCl_3$: MeOH mixture (3: 5: 12) was added and the solution was mixed. 300 µL of chloroform and 450 µL of water were added and the resulting solution was rotated and evaporated up to dryness. The residue was reconstituted in 500 µL of borate buffer (0.1 M, pH 10.2 in HPLC grade water). The extract was filtered using a 0.22 µm filter.

HPLC analysis of free amino acids from *C. reinhardtii*

The o-Phthaldialdehyde (OPA) pre-column (Sigma Aldrich) derivatizing agent was prepared at a concentration of 0.268 M by dissolving it in borate buffer and 0.35% 3-mercaptopropionic acid. Before injecting to the HPLC (HPLC Agilent 1220 Infinity, Agilent Technologies, USA), 20 µL of each standard or sample and 30 µL of borate buffer, were mixed, followed by the addition of 20 µL of OPA. The mixture was deposited in a vial and homogenized before injection (5).

For detection, two mobile phases were used: (A) 0.1 M borate buffer pH 7.8 in HPLC grade water; (B) methanol. The flow rate was 0.8 mL min^{-1}. An isocratic elution was applied: 95% A:5% B. Separation was performed on a reversed phase column ZORBAX Eclipse AAA (150 × 4.6 mm, 3.5 µm) from Agilent Technologies (USA) thermostated at 25 °C. The injection volume was 10 µL and the chromatographic run time set as 20 min. The detection was performed by recording the spectra between 240 nm and 400 nm. The measurement was made at 320 nm.

Vitamin B_{12} deficient *E. coli* mutant and complementation

The *E. coli* mutant (6) was obtained from the DSMZ culture collection (DSM 4261, Braunschweig). Methionine and B_{12} were supplied at 0.2 g/L and 40 µg/L, respectively, to M9 minimal medium (7). A culture of 50 mL of *C. reinhardtii* in TAP was started at an initial density of 1 × 10^5 cell mL^{-1} and the supernatant was collected after 5 days.

Cysteine deficient *E. coli* mutant and complementation

The *E. coli* mutant (YM138, bacterial strain 61953) was obtained from Addgene (USA) (8). Cysteine and methionine were supplied at 0.2 g/L to M9 minimal medium (7). A culture of 50 mL of *C. reinhardtii* in TAP was started at an initial density of 1 × 10^5 cell mL^{-1} and the supernatant was collected after 5 days.

Extraction of B vitamins

Monocultures of *C. reinhardtii* and cocultures of *C. reinhardtii* and *M. lacteus* were prepared in 250 mL of liquid TAP medium (1) in the presence of 0.2% (w/v) glucose. The starting algal cell density was 1 × 10^5 cells mL^{-1} and the bacterial cell density was 2.5 × 10^7 cells mL^{-1}. Cultures were harvested after 5 days and supernatants were evaporated at 50 °C. After drying completely, the residue was reconstituted in 500 µL H_2O (HPLC grade). The extract was passed through a 0.22 µm filter.

The B vitamins were analyzed as described in Glinko et al. (9) with the following specifications. 5 µL of the sample were injected into the HPLC (HPLC Agilent 1220 Infinity, Agilent Technologies). Two mobile phases were used: (A) 25 mM NaH_2PO_4 (pH 2.5); (B) methanol. The flow rate was 1 mL min^{-1}. The gradient was 0% B at 0 min, 0% B at 2 min, 30% B at 8 min and 30% B at 10 min. Separation was performed on a reversed-phase ZORBAX Eclipse Plus (150 × 4.6 mm, 5 µm) column (Agilent Technologies) thermostated at 35 °C. The detection was performed by recording the absorption spectra between 210 nm and 400 nm. The measurement was made at 220 nm.

Quantification of B vitamins and methionine

For quantification, L-methionine as well as vitamins B_1, B_3, B_5 and B_7 were used as standard compounds. Calibration curves were built based on peak areas at 1, 0.5 and 0.05 mg mL^{-1}. L-phenylalanine was used as an internal standard.

Orfamide A cleavage by M. *lacteus* and *M. tolaasinivorans*, extraction and LC-HRMS(MS) measurements

M. lacteus and *M. tolaasinivorans* were grown by inoculating 2 x 15 mL of LB and incubating it at 28 ºC overnight. The overnight cultures were transferred to an ice box. From this point onwards, unless stated otherwise, everything was always kept on ice.

The overnight cultures (30 mL in total) were transferred to a 50 mL falcon tube and centrifuged 10 min at 3435 x *g* and 4 ºC to pellet the bacteria. A 15 mL aliquot of the supernatant was transferred to a new 15 mL falcon tube and kept as "active supernatant". The remaining supernatant was discarded. The pellet was weighed. The right volume (4 mL g^{-1} pellet) of B-PER (Thermo Scientific, No. 90078) was adjusted to pH 7.5 using NaOH/HCl. Lysozyme (50 mg mL^{-1}, included) and DNAse I (2500U mL^{-1}, included) were added at 2 µL mL^{-1} of B-PER. The sample was pipetted up and down until completely solubilized.

In parallel, an Eppendorf with a volume of LB corresponding to the volume of the pellet was prepared and the same amounts of B-Per and enzymes as for the sample above were added. This sample was used as a blank.

The crude lysate and blank were incubated at room temperature for 10 min to enable the cell lysis and immediately transferred to ice and incubated for an extra 30 min. Due to the nature of the enzyme of interest, no proteinase inhibitor was added. For that reason, the time at room temperature was kept as short as possible but long enough for the lysozyme and DNAse I to act.

For both the crude lysate and the blank, a new 15 mL falcon tube was prepared containing a volume of the lysis mixture equivalent to 15 mL of the original culture and filled up to 15 mL with LB medium. The crude lysate sample is called "cell lysate" and the degradation control "orfamide A" in Fig. 3*B*.

Two heat-treated controls of the cell lysate and the active supernatant were prepared by autoclaving the samples (121°C, 15 min).

Orfamide A was then added to each sample to a final concentration of 20 µM. The samples were transferred to 28 ºC in a Hulamixer at low speed. An aliquot of 2 mL was taken after 24 h for extraction.

Orfamide A and its degradation products were extracted by adding an equal volume of ethyl acetate to the sample and incubating at room temperature with gentle shaking for 30 min. The ethyl acetate phase was then recovered, and the procedure was repeated one more time. The combined ethyl acetate phases were dried in a rotary evaporator. Once the samples were fully dried, 200 µL of HPLC-grade methanol was added and the sample was filtered through a 0.22 µm PFTE filter.

Samples were run in a Q-exactive LC-HRMS device as described below.

Orfamide A cleavage by *M. lacteus* used for the determination of the α-fragmentation in the double-cleaved degradation product (*m/z* 1013.66)

M. lacteus was grown by inoculating 15 mL of King's B media and incubating it at 28 ºC overnight. The overnight culture (1 mL) was transferred to an Eppendorf tube and centrifuged at 11000 x g. The supernatant was discarded, and the pellet was resuspended in 200 µL of fresh Kings B media. Cells were lysed by applying 3 rounds of sonication using a Sonotrode on ice. Orfamide A (1 mg/ml) was then added and incubated overnight at 28 ºC. An equal volume of methanol (200 µL) was added, and the sample was filtered through a 0.22 µm PFTE filter. Samples were then run in a Q-exactive LC-HRMSMS device as described below.

LC-HRMS(MS) measurements

HPLC-HRESI-MS and MS/MS measurements were performed using a QExactive Orbitrap High Performance Benchtop LC-MS with an electron spray ion source and an UltiMate™ 3000 HPLC System with PDA (Thermo Fisher Scientific, Germany) employing a C18 column (Accucore C18 2.6 µm, 100 × 2.1 mm, Thermo Fisher Scientific, Germany) and the following solvent system: acetonitrile and distilled water (both supplemented with 0.1% formic acid), flow rate: 0.2 mL min^{-1}; program: 0-10 min gradient 5–98% acetonitrile, hold until 14 min 98% acetonitrile. Compounds were identified by comparison with synthetic references (retention time, UV spectrum, HR-MS and MS/MS spectra).

Phylogeny of hydrolases from *M. lacteus* including putative enzymes degrading orfamide A

The starting reference includes a list of 22 proteins representing all families of proteases and esterases, including known CLiP-degrading enzymes (Table S3). That reference list was used

to run BLAST on annotated genes of *M. lacteus* and identify putative genes coding for proteases/esterases. The candidates found in the genome were then used to find the closest homologs in UniprotKB and in PDB (Table S3). The candidates from *M. lacteus*, the homologs and the reference proteins were then used to generate a Multiple Sequence Alignment using MAFFT-DASH with default settings and Mafft-homologs on. The extra homologs found by MAFFT-DASH during the alignment generation were also kept in the MSA. The phylogenetic tree was then generated using IQ-tree with the following settings: -ninit 1000 -bb 5000 -bnni -alrt 5000 -wbt -nstop 1000 -st AA -nm 5000 -wbtl -m WAG+R8 -nt AUTO.

Microbiota reanalysis for the presence of *Chlamydomonas*, *Pseudomonas* and *Mycetocola*

Three microbiotas (A, rice wash water microbiome; B, *C. reinhardtii* soil microbiome and C, rhizosphere microbiomes of potato) were reanalyzed for the presence of *Pseudomonas* and *Mycetocola* together with *Chlamydomonas*. The selected sequencing runs were downloaded from the NCBI SRA server. For each project, all the sequencing runs were merged and split into forward and reverse reads when needed. To identify the bacteria, the merged sequencing runs were uploaded to One codex for classification. For the identification of *C. reinhardtii* within the samples, BLAST was used to search the sequencing reads against the nuclear genome or, in the case of 16S-amplified libraries, against the chloroplast genome. Matching reads were then searched using BLAST against the full NCBI nucleotide collection to verify that the best match belonged to *Chlamydomonas*.

(A) Rice microbiome from rice wash water (10). The inoculum from the water of a rice crop field was used to do Illumina metagenomic WGS in which *Chlamydomonas*, *Mycetocola* and *Pseudomonas* were detected [Supplemental Table 2 in the preprint (10) and verified in the raw data].

PRJNA592260 - SRP234851 (SRR10595456).

(B) *C. reinhardtii* soil microbiome: Shared features and reciprocal complementation of the *Chlamydomonas* and *Arabidopsis* microbiota (11). From the selected 16S PCR amplified sequence libraries chlamy_011 and chlamy_018 which correspond to the pot experiments and the surface soil samples, both *Mycetocola* and *Pseudomonas* were detected.

PRJEB43117 - ERP127048 (ERR7265013, ERR7265014, ERR7265015, ERR7265016, ERR7265017, ERR7265018, ERR7265019, ERR7265020, ERR7265021, ERR7265022, ERR7265023, ERR7265024, ERR7265025, ERR7265026, ERR7265027, ERR7265028, ERR7265029, ERR7265030, ERR7265031, ERR7265032, ERR7265033, ERR7265034, ERR7265035, ERR7265036, ERR7265037, ERR7265038, ERR7265039, ERR7265040,

ERR7265041, ERR7265042, ERR7265043, ERR7265044, ERR7265045, ERR7265046, ERR7265047, ERR7194249, ERR7194250, ERR7194251, ERR7194252, ERR7194253, ERR7194254, ERR7194300, ERR7194301, ERR7194302, ERR7194303, ERR7194304, ERR7194305, ERR7194306, ERR7194307, ERR7194308, ERR7194309, ERR7194310, ERR7194311, ERR7194312, ERR7194313, ERR7194314, ERR7194315, ERR7194316, ERR7194317, ERR7194335, ERR7194336, ERR7194337, ERR7194338, ERR7194339, ERR7194340).

(C) Rhizosphere microbiomes of potato cultivated in the High Andes show stable and dynamic core microbiomes with different responses to plant development (12)

PRJNA226221 - SRP032503 (all runs): Both *Mycetocola* and *Pseudomonas* are found in the soil samples. *Chlamydomonas* chloroplastic 16S could also be found (SRR1175872.HJVZO6O02EY1TP, SRR1175874.HJVZO6O02ET8FY, SRR1175860.HJVZO6O01CCX81) among other microalgae.

Chemical synthesis of orfamide A fragments including Fig. S18-S23 and Table S2

1. Chemical Methods

1.1 List of chemical abbreviations

A.A.	Amino acid
Alloc	allyloxycarbonyl
Boc	*tert*-butyloxycarbonyl
DIC	*N,N'*-Diisopropylcarbodiimide
DIEA	*N,N*-Diisopropylethylamine
DMAP	4-Dimethylaminopyridine
DMF	*N,N*-Dimethylformamide
DMSO	Dimethylsulfoxide
eq.	Stoichiometric equivalent
F.A.	Fatty acid
Fmoc	9-fluorenylmethyloxycarbonyl
HATU	1-[Bis(dimethylamino)methylene]-1*H*-1,2,3-triazolo[4,5*b*]pyridinium3-oxid hexafluorophosphate
HBTU	*N,N,N',N'*-Tetramethyl-*O*-(1*H*-benzotriazol-1-yl)uronium hexafluorophosphate
HFIP	Hexafluoro-2-propanol
HOAt	1-Hydroxy-7-azabenzotriazole
HOBt	1-Hydroxybenzotriazole
SPPS	Solid phase peptide synthesis
TBS	*tert*-butyldimethylsilyl
THF	tetrahydrofran
TIS	Triisopropylsilane

1.2 Synthesis of the orfamide A degradation products

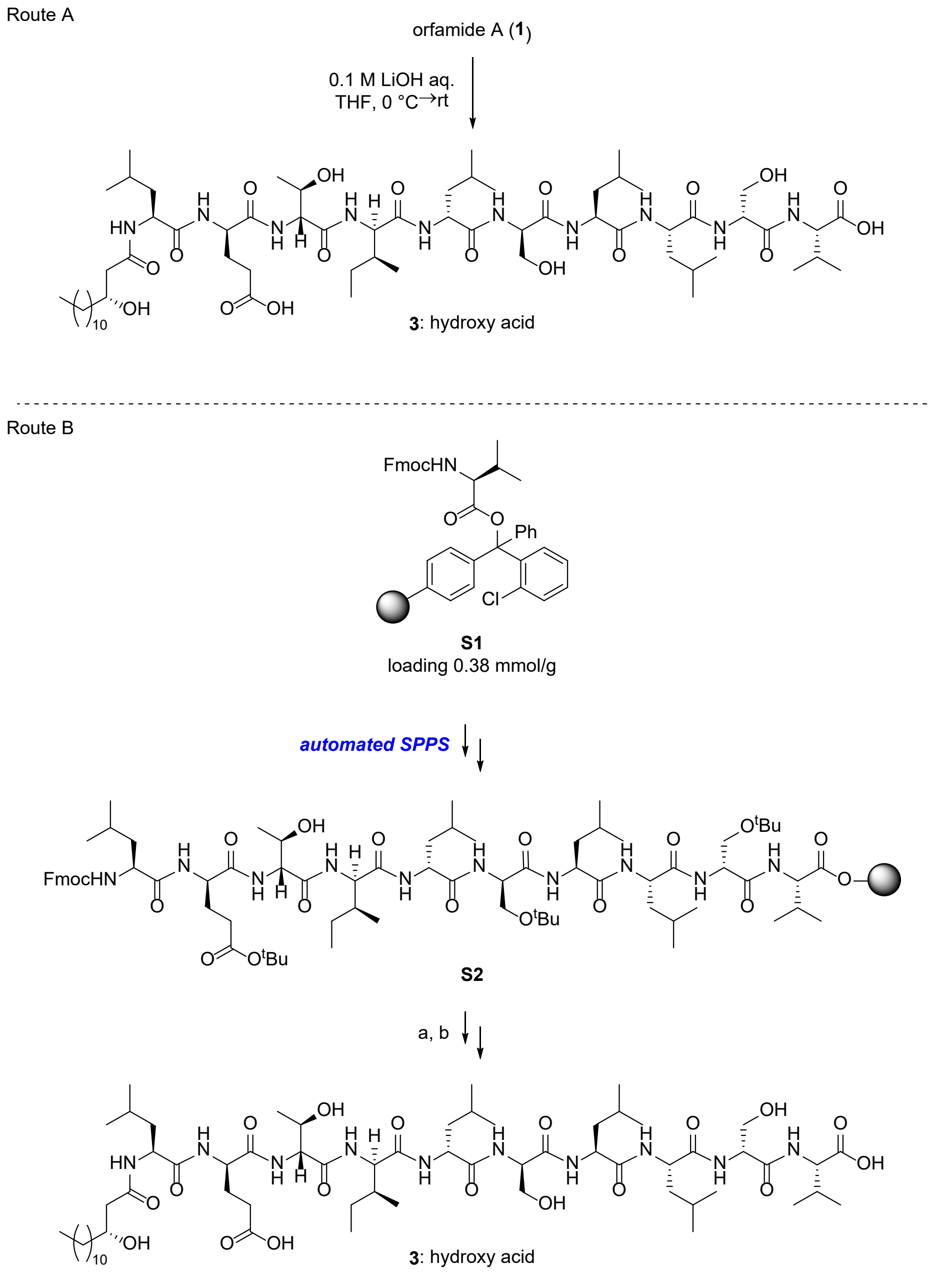

Reagents and conditions for Route B: a) 20% piperidine/DMF (v/v), rt; (*R*)-3-TBSoxytetradecanoic acid (1.5 eq.), HBTU (1.5 eq.), HOBt (1.5 eq.), DIEA (3 eq.), DMF (0.4 M to F.A.), rt, 4 h; (*R*)-3-TBSoxytetradecanoic acid (1.2 eq.), HATU (1.2 eq.), HOAt (1.2 eq.), DIEA (2.4 eq.), DMF (0.32 M to F.A.), rt, 28 h; b) 0.1 N HCl/HFIP + 1% TIS, rt (13, 14).

Fig. S18: Synthesis of the degradation product of orfamide A (**3**)

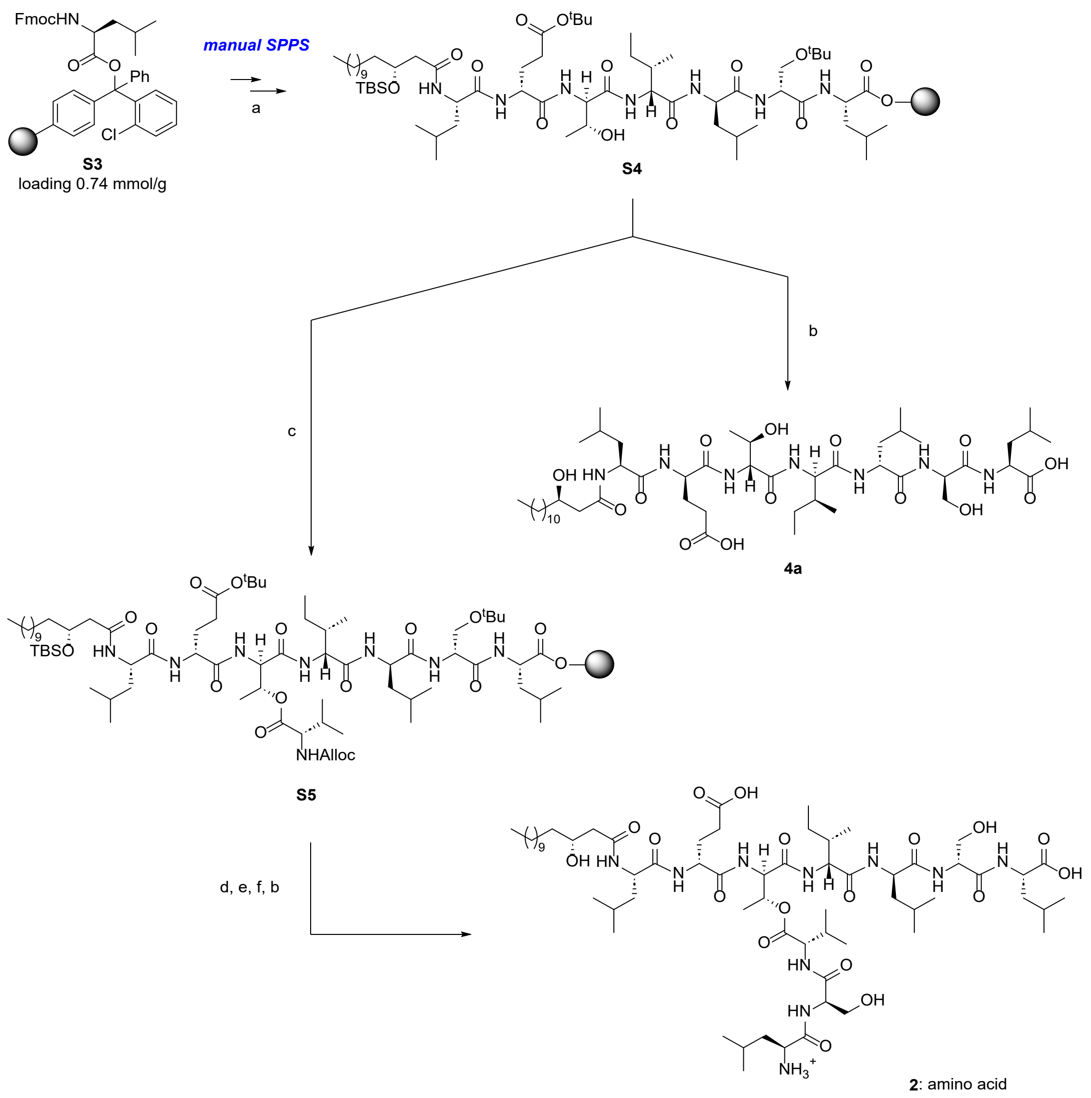

Reagents and conditions: a) 20% piperidine/DMF (v/v), rt; Fmoc-A.A. or (*R*)-TBSoxytetradecanoic acid (2 eq.), HBTU (2 eq.), HOBt (2 eq.), DIEA (4 eq.), DMF (0.4 M to A.A.or F.A.), rt, 2 h; b) 0.1 N HCl/HFIP + 1% TIS, rt (13, 14); c) Alloc-L-Valine, DIC, DMAP, THF, 0 °C, rt; d) *cat.* $Pd(PPh_3)_4$, phenylsilane, CH_2Cl_2, rt; e) Fmoc-D-Ser(O^tBu)-OH (4 eq.), HBTU (4 eq.), HOBt (4 eq.), DIEA (8 eq.), DMF (0.25 M to A.A.), rt, 2 h; 20% piperidine/DMF (v/v), rt; f) Boc-L-Leu-OH(4 eq.), HBTU (4 eq.), HOBt (4 eq.), DIEA (8 eq.), DMF (0.25 M to A.A.), rt, 1.5 h.

Fig. S19: Synthesis of **2** and the degradation product of orfamide A (**4a**)

1.3 General Synthesis Methods

1.3.1 Reagents and reaction conditions

All reagents were purchased from Acros Chemicals, Alfa Aesar, ABCR, Carbolution Chemicals, Carbosynth, Fischer Chemical, fluoroChem, GL Biochem (Shanghai), GRÜSSING, Manchester Organics, Merck, Novabiochem, Sigma-Aldrich, TCI Europe and VWR. All solvents, if not purchased in purity or dryness suitable, were distilled. Deionized water was used for all experiments.

1.3.2. NMR spectroscopy

^{1}H-NMR spectra were recorded using Bruker Advance III HD 500 or Advance III 600 system. Spectra were calibrated to appropriate residual solvent peaks (methanol-d_4, DMSO-d_6) (15).

1.3.3 Analytical reverse phase HPLC (RP-HPLC)

Analyses were performed on a SHIMADZU system consisting of a system controller (SLC-10A VP), a column oven (CTO-10AC VP), an auto-injector (SIL-10ADVP), a degasser (DGU-14A), three pumps (LC-10AT VP), a diode array detector (SPD-M20A), a fluorescence detector (RF-10AXL) and an analytical column. The column was equilibrated to starting condition of each method prior to sample injections.

Eluent System: A = acetonitrile, B = water, C = 2% TFA in water, Flow rate: 1 mL/min, Column oven: 25 °C, Detection: diode array 190 – 800 nm.

Column: MACHEREY-NAGEL NUCLEODUR C18 Gravity, 5 µm, 125 × 4 mm.

RP-HPLC Method 50

Gradient: Eluent A: 50% (1 min), 50-95% (10 min), 95% (5 min), 95-50% (0.2 min), 50% (5.8 min). Eluent C: 5% (22 min).

RP-HPLC Method 70

Gradient: Eluent A: 70% (1 min), 70-95% (10 min), 95% (5 min), 95-70% (0.2 min), 70% (5.8 min). Eluent C: 5% (22 min).

1.3.4 Reverse phase preparative HPLC (RP-Prep. HPLC)

Purifications of final products were performed on SHIMADZU systems. Columns were equilibrated to starting conditions of each method prior to sample injections. Eluent System: A = 0.1% TFA in acetonitrile, B= 0.1% TFA in water, Detection: UV-VIS 220 nm. Flow rate: 10 mL/min.

Prep.-RP-HPLC-A consists of a communications bus module (CBM-20A), two pumps (LC-20AP), a column oven (CTO-10AC) and an UV-VIS detector (SPD-10AVP).

Prep.-A Method 60

Column: MACHEREY-NAGEL NUCLEODUR C18 Gravity, 5 µm, 250 × 16 mm.

Gradient: Eluent A: 60-70% (35 min), 70-80% (10 min), 80-95% (5 min), 95% (10 min), 95-60% (2 min), 60% (10 min).

Prep.-A Method 70

Column: M&W Chromatographietechnik GmbH Hypersil ODS, 5 µm, 250 × 20 mm.

Gradient: Eluent A: 70% (1 min), 70-95% (34 min), 95% (15 min), 95-70% (2 min), 70% (10 min).

Prep.-A Method 80

Column: MACHEREY-NAGEL NUCLEODUR C18 Gravity, 5 µm, 250 × 16 mm.

Gradient: Eluent A: 80% (1 min), 80-95% (20 min), 95% (5 min), 95-80% (2 min), 80% (25 min).

Prep.-RP-HPLC-B consists of a system controller (SCL-10A), two pumps (LC-8A), and an UV-VIS detector (SPD-10AVP).

Prep.-B Method 70

Column: MACHEREY-NAGEL NUCLEODUR C18 Gravity, 5 µm, 250 × 16 mm.

Gradient: Eluent A: 70-75% (10 min), 75-80% (10 min), 80-95% (15 min), 95% (10 min), 95-70% (2 min), 70% (13 min).

1.3.5 Liquid chromatography mass spectrometry (LC/MS)

Analyses were performed on a SHIMADZU system consisting of a system controller (SLC-10A VP), a column oven (CTO-10AC VP), an auto-injector (SIL-10AD VP), a degasser (DGU-14A), two pumps (LC-10AT VP), an analytical column (MACHEREY-NAGEL NUCLEODUR C18 Isis, 3 µm), a post-column flow splitter (Thermo scientific, ICP-04-20), a UV-VIS detector (SPD-10A VP) and an ESI ion trap mass spectrometer (Finnigan LCQ spectrometer). The column was equilibrated to starting conditions of each method prior to the sample injections.

Eluent System: A = 0.1% HCOOH in acetonitrile, B= 0.1% HCOOH in water, Flow rate: 1 mL/min, Detection: UV/VIS 220 or 254 nm, Column oven: 25 °C.

1.3.6 Liquid chromatography high resolution mass spectrometry (LC/HRMS)

Analyses were performed on a Thermo scientific UltiMate 3000 UHPLC system consisting of a pump, an auto sampler, a column compartment, a diode array detector and a high resolution Q-TOF MS spectrometer (maXis Impact, BRUKER DALTRONICS, Bremen Germany). The column was equilibrated to starting conditions of each method prior to the sample injections. Eluent System: A = 0.1% HCOOH in acetonitrile, B= 0.1% HCOOH in water, Flow rate: 0.5 mL/min, Detection: Diode array 200 - 400 nm.

1.4 Synthesis procedures and physical data

1.4.1 Synthesis of hydroxy acid (3) by route A (Fig. S18)

0.1 M LiOH (aq.) (in total 369 μL) was added by portion to the solution of synthetic orfamide A (**1**) (1.6 mg, 1.23 μmol) in THF (0.25 mL) and stirred at 0 °C for 4 h. Then the reaction mixture was warmed up to room temperature and stirred for another 15 h. The reaction was quenched by addition of 1M HCl (aq.) and the resulting mixture was purified on RP-Prep-HPLC (Prep.-B Method 70) to give **3** in less than 1 mg as a colorless glass. **3** obtained from Route A was used as a standard for the LC-HRMS/MS analysis. This procedure had different outcomes by batch to batch, and therefore **3** was also synthesized by route B for obtaining a substantial amount for the biological assays.

RP-HPLC: t_R = 10.2 min (Method 70)

HRMS (ESI-TOF) calculated for $C_{64}H_{116}N_{10}O_{18}$ $[M+H]^+$ 1313.8542; found 1313.8548.

1.4.2 General procedures for solid phase peptide synthesis

All peptide syntheses were performed on solid support. Reactions were monitored by small scale cleavage of the peptidyl resin using one of the following conditions and subjecting the supernatant after the work up (evaporation, dissolving the residue in acetonitrile or MeOH and then filtration) to LC/MS and/or RP-HPLC analyses. Amounts of reagents and solvents used as well as yields were calculated based on the initial amino acid loading on resin. Synthetic orfamide A (**1**) and (*R*)-TBSoxytetradecanoic acid were synthesized as reported previously (16).

<u>Peptide cleavage conditions</u>

Condition A: 30% HFIP in CH_2Cl_2 (v/v), rt, 15 min.

Condition B: 0.1 N HCl/HFIP (10 μL 37% aq. HCl / 990 μL HFIP) + 1% TIS, rt, 30 – 60 min (13, 14).

1.4.2.1 Fmoc amino acid loading on 2-chlorotrityl chloride resin

Fmoc-L-Val-OH (**S1**) loading (Fig. S18, Route B):

2-chlorotrityl chloride resin (302 mg of 1.50 mmol/g, 0.453 mmol, 1 eq.) was swollen with CH_2Cl_2 (3 mL) for 20 min (2 ×). Then the solution of Fmoc-L-Val-OH (46.2 mg, 0.136 mmol, 0.3 eq.) and DIEA (237 µL, 1.36 mmol, 3 eq.) in CH_2Cl_2 (3 mL) was added to a syringe containing resin and the syringe was shaken for 2 h at room temperature. The resin was washed with CH_2Cl_2 (6 ×) and then treated with a solution of CH_2Cl_2/MeOH/DIEA (17:2:1, v/v/v) for 20 min (2 ×). After the removal of solvents the resin was washed with CH_2Cl_2 (3 ×), DMF (3 ×), CH_2Cl_2 (3 ×) and MeOH (1 ×) and dried in vacuo overnight. The loading was determined to be 0.38 mmol/g by a small scale Fmoc cleavage using 20% piperidine/DMF (v/v) and measurement of the absorption of the cleaved piperidine-dibenzofulvene adduct.

Fmoc-L-Leu-OH loading (**S3**) (Fig. S19):

2-chlorotrityl chloride resin (300 mg of 1.60 mmol/g, 0.480 mmol, 1 eq.) was swollen with CH_2Cl_2 (3 mL) for 20 min (2 ×). Then the solution of Fmoc-L-Leu-OH (102 mg, 0.289 mmol, 0.6 eq.) and DIEA (418 µL, 2.40 mmol, 5 eq.) in CH_2Cl_2 (3 mL) was added to a syringe containing resin and the syringe was shaken for 2 h at room temperature. The resin was washed with CH_2Cl_2 (6 ×) and then treated with a solution of CH_2Cl_2/MeOH/DIEA (17:2:1, v/v/v) for 20 min (2 ×). After the removal of solvents the resin was washed with CH_2Cl_2 (3 ×), DMF (3 ×), CH_2Cl_2 (3 ×) and MeOH (1 ×) and dried in vacuo overnight. The loading was determined to be 0.74 mmol/g by a small scale Fmoc cleavage using 20% piperidine/DMF (v/v) and measurement of the absorption of the cleaved piperidine-dibenzofulvene adduct.

1.4.2.2 Fmoc deprotection conditions

The deprotection solution (20% piperidine/DMF (v/v)) was added to the peptidyl resin in a syringe and the syringe was shaken for 5 min at room temperature. The Fmoc deprotection solution was removed, and the deprotection step was repeated once for another 15 min, and the resulting peptidyl resin was washed with DMF (6 ×) prior to the subsequent coupling.

1.4.2.3 SPPS using an automated parallel peptide synthesizer (Fig. S18, Route B)

An automated peptide synthesizer (MultiSyn Tech, Syro ll) was programmed to couple amino acids sequentially from *C*-terminus to *N*-terminus for **S2** synthesis. A single coupling cycle consisted of Fmoc deprotection, amino acid coupling and washing with DMF (3 ×) after each step. All steps were performed at room temperature in open air. The detailed protocol used to

program the peptide synthesizer is described in Table S2. The resulting peptidyl resin was washed with CH_2Cl_2 (3 ×) and MeOH (1 ×) and was dried under high vacuum.

Table S2: One cycle of the SPPS using automated peptide synthesizer (Syro ll)

Step	Operation	Solvent/Reagent	Time (repetitions)
1	Swelling	DMF	20 min 30 s (2 ×) [a, b]
2	Fmoc deprotection	20 % piperidine in DMF (v/v)	3 min (1 ×) [a, c]<br>7 min (2 ×) [a, c]
3	Wash	DMF	3 min (3 ×) [a, c]
4	Coupling	Fmoc-A.A. solution in DMF (0.4 M, 4 eq.); add HBTU/HOBt in DMF (0.3 M each, 4 eq.); add DIEA in NMP (1.3 M)	1 or 2 h (1 ×) [a, d]
5	Wash	DMF	3 min (6 ×) [a, c]

[a] Vortex = 20 sec.; [b] Break = 3 min; [c] Break = 1 min; [d] Break = 5 min.

1.4.2.4 Manual SPPS

The resin was swollen prior to all steps. After Fmoc deprotection, the coupling cocktail of corresponding amino acids or the β-hydroxy fatty acid were added to the syringe containing peptidyl resin and shaken till the completion of the coupling. The coupling step was repeated if necessary. The reagents were removed and the resulting resin were washed with DMF (6 ×) prior to the next step. When needed, after evaporation of the solvents under reduced pressure, the peptidyl resin was stored at -25 °C in the dark.

Coupling cocktail: A.A. or building block (1.2 - 4 eq. to the peptidyl resin), HBTU or HATU (1.2 - 4 eq. to the peptidyl resin), HOBt or HOAt (1.2 - 4 eq. to the peptidyl resin), DIEA (2.4 - 8 eq. to the peptidyl resin) in DMF (0.25 - 0.4 M; to A.A. or F.A.). The conditions for each coupling are indicated in the Fig. S18-S19 above.

1.4.2.5 On-resin esterification (manual SPPS) (Fig. S19)

To the reaction flask containing peptidyl resin (**S4**) (115 mg, 59.5 µmol, 1 eq.) were added Alloc-L-Valine (120 mg, 596 µmol, 10 eq.), DIC (92 µL, 594 µmol, 10 eq.) and DMAP (7.3 mg, 59.8 µmol, 1 eq.) in THF (0.5 M to Alloc-L-Valine) at 0 °C. The temperature was allowed to warm up to room temperature and the reaction was stirred for 18 h. The reaction

mixture was removed and the peptidyl resin was washed with CH_2Cl_2 (3 ×), DMF (3 ×), CH_2Cl_2 (3 ×) and MeOH (1 ×). The resulting peptidyl resin was dried under high vacuum.

1.4.2.6 Alloc deprotection (manual SPPS) (Fig. S19)

The peptidyl resin (**S5**) (104.5 mg, 52.5 µmol, 1 eq.) was dried under high vacuum prior to Alloc deprotection and the resin was swollen in CH_2Cl_2 under Ar atmosphere. A solution of $Pd(PPh_3)_4$ (15.5 mg, 13.4 µmol, 0.26 eq.) and $PhSiH_3$ (170 µL, 1.34 mmol, 26 eq.) in CH_2Cl_2 (3 mL) was added to the reaction pot containing the resin and the mixture was stirred at room temperature for 2 h in the dark. The resin was washed with CH_2Cl_2 (3 ×), DMF (3 ×), CH_2Cl_2 (3 ×) and MeOH (1 ×). The resulting peptidyl resin was dried under high vacuum, and a part of the resulting dried peptidyl resin (45.5 mg, 26 µmol) was used for the final steps of amino acid **2** synthesis.

1.4.2.7 Cleavage and global deprotection of peptide from resin

Peptidyl resin was dried under high vacuum prior to peptide cleavage from resin. Peptides were cleaved from resin by cleavage condition B (ca. 100 mg peptidyl resin/1 mL cleavage cocktail). After the addition of the cleavage cocktail, the syringe containing peptidyl resin was shaken for 20 min at room temperature and the solution was collected in a round bottom flask. This procedure was repeated three times. The resulting mixture was stirred further at room temperature until complete deprotection was observed by LC/MS. Then the mixture was evaporated with toluene and the crude mixture was purified by RP-prep. HPLC.

1.5 Analytical data of the synthesized lipopeptides by SPPS

1.5.1 amino acid (2)

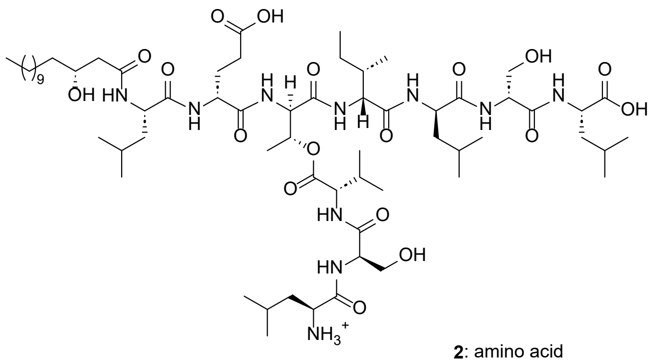

2: amino acid

After the RP-Prep-HPLC purification (Prep.-A Method 60), **2** was obtained as colorless glass (9.9 mg, 6.93 µmol, 27% from resin loading 0.74 mmol/g, based on the calculation as mono(trifluoroacetate)).

RP-HPLC: t_R = 10.1 min (Method 50)

HRMS (ESI-TOF) calculated for $C_{64}H_{116}N_{10}O_{18}$ $[M+H]^+$ 1313.8542; found 1313.8541.

^{1}H-NMR (MeOH-d$_4$, 500 MHz, 297 K) δ =0.86 - 1.03 (m, 39 H), 1.18 (m, 1 H), 1.24 - 1.37 (m, 20 H), 1.37 – 1.53 (m, 4 H), 1.56 – 1.83 (m, 12 H), 1.95 (m, 2 H), 2.14 (m, 1 H), 2.23 (m, 1 H), 2.32 (dd, J = 14.2, 8.7 Hz, 1 H), 2.39 (m, 2 H), 2.44 (dd, J = 14.2, 3.9 Hz, 1 H), 3.76 (dd, J = 11.1, 5.3 Hz, 1 H), 3.81 (m, 2 H), 3.85 (dd, J = 11.1, 6.0 Hz, 1 H), 4.01 (m, 2 H), 4.31 (m, 2 H), 4.37 (d, J = 5.1 Hz, 1 H), 4.41 – 4.49 (m, 4 H), 4.70 (d, J = 9.2 Hz, 1 H), 4.76 (t, J = 6.3 Hz, 1 H), 5.11 (m, 1 H) ppm.

1.5.2 hydroxy acid (3)

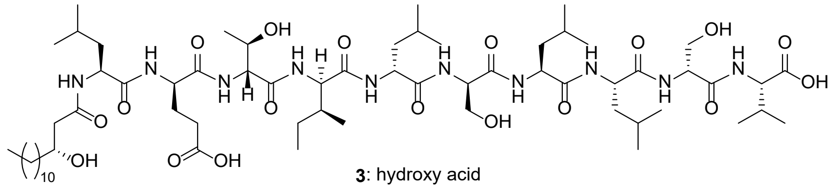

3: hydroxy acid

Route B:

After the RP-Prep-HPLC purification (method: Prep.-A Method 80), **3** was obtained as colorless glass (31.9 mg, 24.3 µmol, 60% from resin loading 0.38 mmol/g). **3** obtained from Route B was used for measuring the biological activities (Fig. 4).

RP-HPLC: t_R = 10.2 min (Method 70)

^{1}H-NMR (DMSO-d$_6$, 600 MHz, 297 K) δ = 0.78 - 0.95 (m, 39 H), 1.08 (d, J = 7.9 Hz, 3 H), 1.12 (m, 1 H), 1.16 - 1.34 (m, 21 H), 1.35 - 1.51 (m, 8 H), 1.53 - 1.63 (m, 4 H), 1.71 (m, 1 H), 1.89 (m, 2 H), 2.04 (m, 1 H), 2.12 - 2.26 (m, 4 H), 3.51 - 3.57 (m, 3 H), 3.62 (dd, J = 10.6, 6.2 Hz, 1 H), 3.76 (m, 1 H), 3.82 (m, 1 H), 4.17 (dd, J = 8.8, 6.2 Hz, 1 H), 4.23 (m, 1 H), 4.24 - 4.33 (m, 6 H), 4.34 (m, 2 H), 7.81 (d, J = 8.6 Hz, 2 H), 7.73 - 7.88 (m, 4 H), 7.91 (d, J = 8.2 Hz, 1 H),7.93 (d, J =7.8 Hz, 1 H), 7.98 (d, J = 7.9 Hz, 1 H), 8.13 (d, J = 7.8 Hz, 1 H), 12.34 (br s, 2 H) ppm.

HRMS (ESI-TOF) calculated for $C_{64}H_{116}N_{10}O_{18}$ $[M+H]^+$ 1313.8542; found 1313.8539.

1.5.3 4a

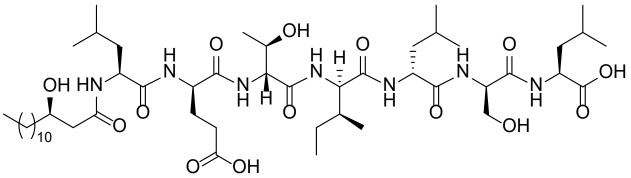

4a

After the RP-Prep-HPLC purification (method: Prep.-A Method 70), **4a** was obtained as colorless glass (9.9 mg, 9.76 µmol, 40% from resin loading 0.74 mmol/g).

RP-HPLC: t_R = 11.8 min. (Method 50)

^{1}H-NMR (DMSO-d_6, 500 MHz, 297 K) δ = 0.73 – 0.94 (m, 27 H), 1.09 (d, J = 6.1 Hz, 3 H), 1.12 (m, 1 H), 1.17 – 1.38 (m, 22 H), 1.38 – 1.66 (m, 8 H), 1.71 (m, 1 H), 1.90 (m, 2 H), 2.20 (m, 4 H), 3.55 (m, 2 H), 3.77 (m, 1 H), 3.82 (m, 1 H), 4.22 (m, 1 H), 4.26 – 4.32 (m, 5 H), 4.36 (q, J = 7.9 Hz, 1 H), 4.58 (brs, 1 H), 4.82 (brs, 1 H), 5.12 (d, J = 4.9 Hz, 1 H), 7.77 (d, J = 8.0 Hz, 1 H), 7.81 (d, J = 8.6 Hz, 1 H), 7.84 (d, J = 8.3 Hz, 1 H), 7.89 (d, J = 8.2 Hz, 1 H), 7.92 (d, J = 8.1 Hz, 1 H), 7.97 (d, J = 7.8 Hz, 1 H), 8.11 (d, J = 7.9 Hz, 1 H), 12.28 (brs, 2 H) ppm.

HRMS (ESI-TOF) calculated for $C_{50}H_{91}N_7O_{14}$ $[M+H]^+$ 1014.6697; found 1014.6699.

1.6 Copies of NMR spectra

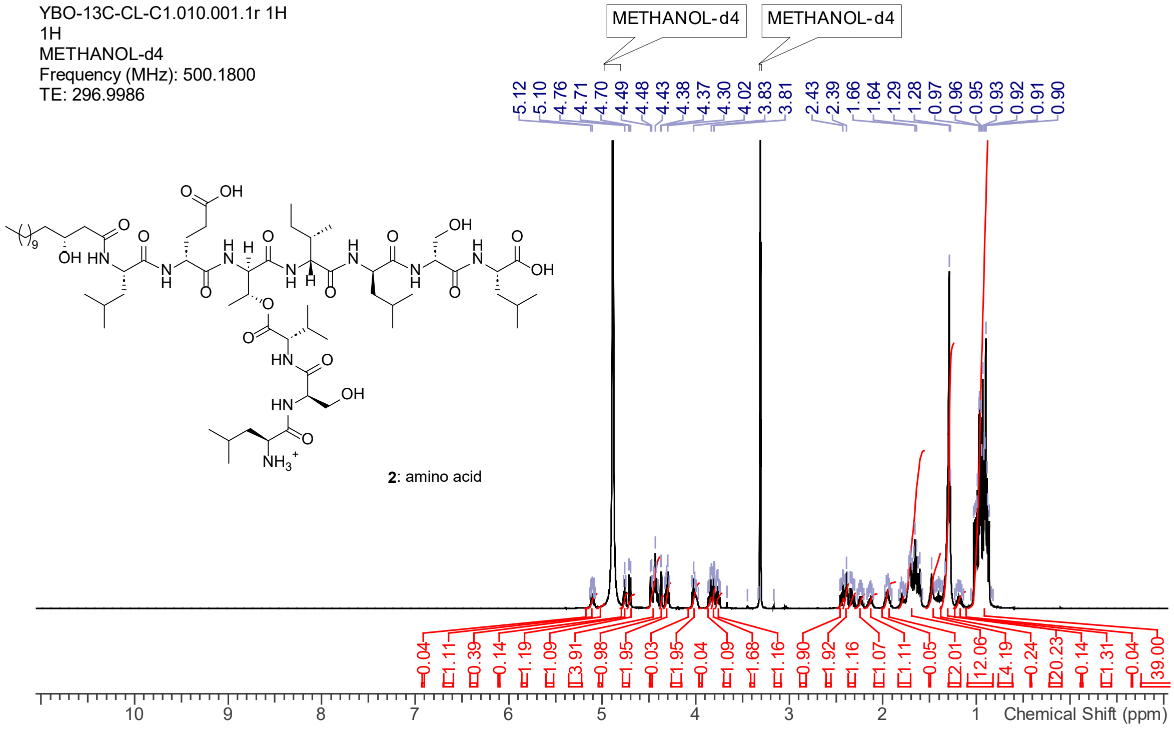

Fig. S20: ^{1}H NMR spectrum of **2**

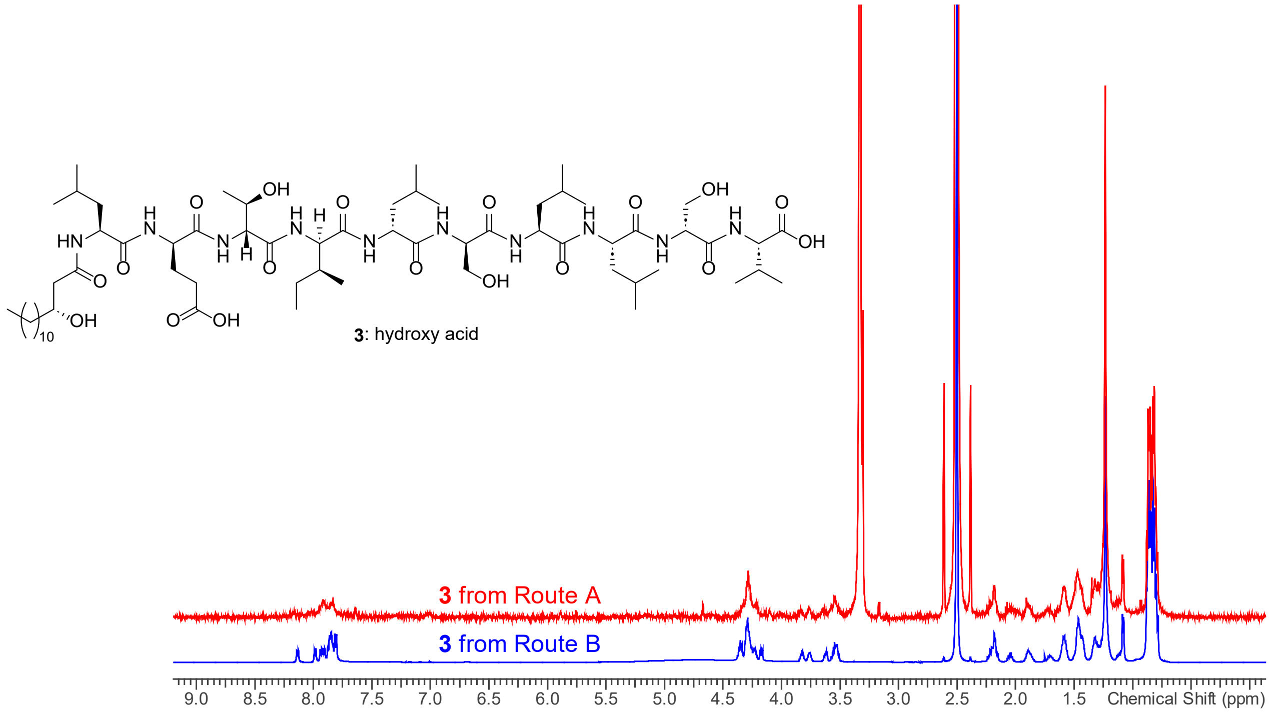

Fig. S21: ^{1}H NMR spectrum (500 MHz, DMSO-d6) of 3 from Route A (Red, top) overlaid to the ^{1}H NMR spectrum (600 MHz, d-DMSO6) of **3** from Route B (Blue, bottom).

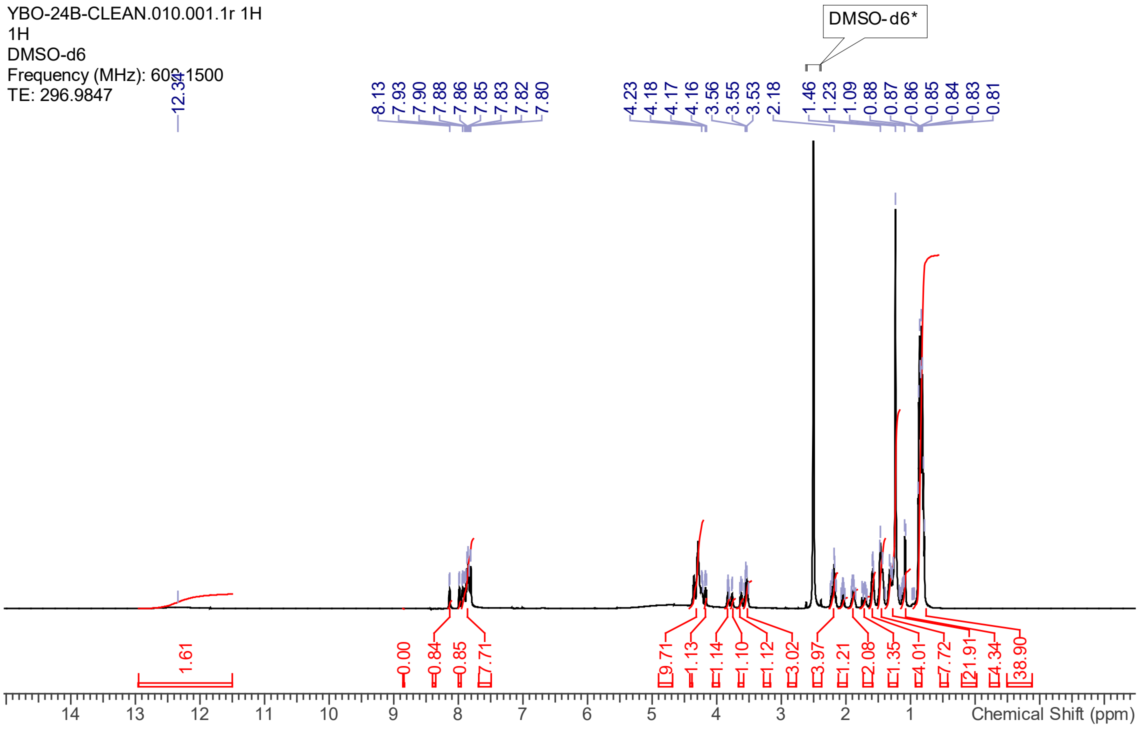

Fig. S22: ^{1}H NMR spectrum of **3** from Route B

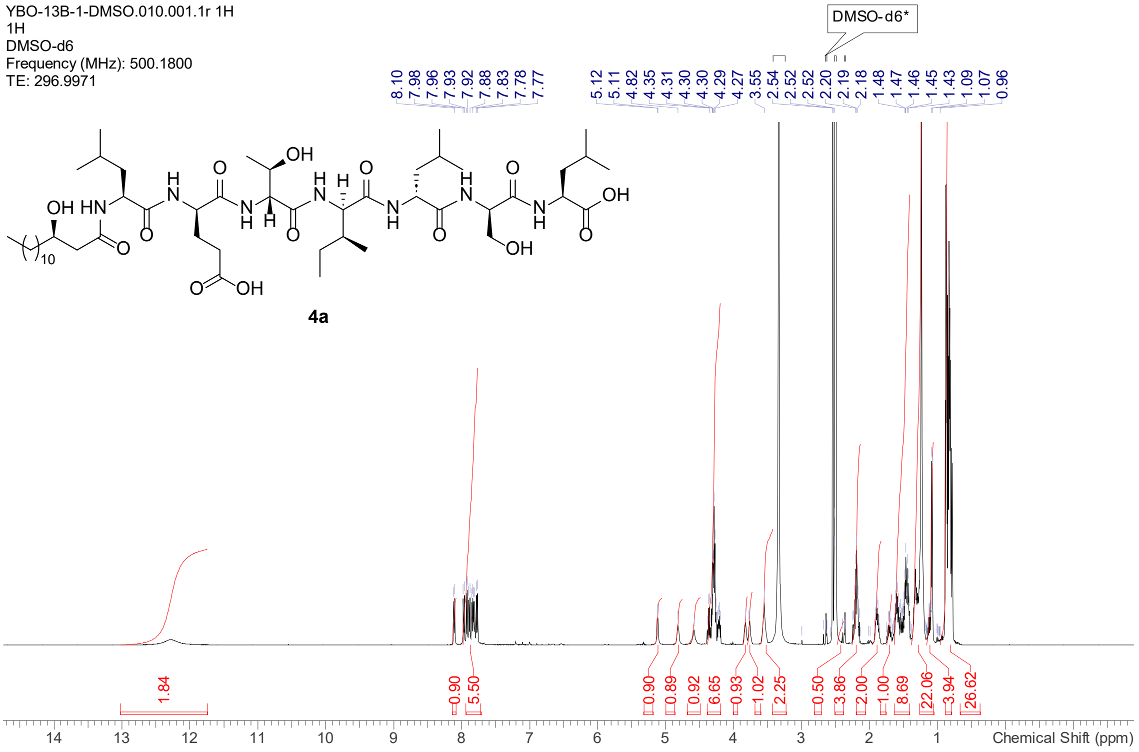

Fig. S23: ^{1}H NMR spectrum of **4a**

Ca^{2+} assay with an aequorin reporter system

Ca^{2+} measurements were adapted from Aiyar et al. (17). Cells from the transgenic apo-aequorin expressing line AEQ34 were grown in TAP medium for 48 hours at 23 °C within a light-dark (LD) 12:12 cycle, and then incubated with coelenterazine overnight. The cell density was adjusted to 4–5 × 10^6 cells · mL^{-1} prior to measurements. Obtained relative luminescence units (RLUs) were converted to molar Ca^{2+} concentrations as described in Fricker et al. (18).

Deflagellation assay

The experiments were performed according to Aiyar et al. (17) with some modifications. Cells from the *C. reinhardtii* wild-type strain SAG 73.72 were treated with 5 µM of compounds for 1 min followed by fixation with 10% (v/v) Lugol's Solution (MERCK, Germany). The cells were imaged with a DIC microscope (Axiophot, Zeiss).

Table S3: List of putative hydrolases and esterases capable of cleaving orfamide A

Reference sequences	*M. lacteus* candidate proteins	BLAST homologs	MAFFT-homologs	PDB-DASH homologs
ref|ABI94047	D9V34_00280|RLP84482	pdb|4YPV	ho|UniRef50_A0A067FPF9	dash|5JD4
ref|AEO72359	D9V34_00490|RLP84519	sp|A0A0E4AET8	ho|UniRef50_A0A068Z267	dash|5JD6
ref|AEO72360	D9V34_01640|RLP84724	sp|A0A2I4HXH5	ho|UniRef50_A0A075QZL7	dash|5NFQ
ref|AEO72362	D9V34_01735|RLP84741	sp|A0QNL1	ho|UniRef50_A0A077WCT3	dash|5NG7
ref|CAE80483	D9V34_03895|RLP83952	sp|A1VUV0	ho|UniRef50_A0A094PPU0	dash|6EB3
ref|EDX21764	D9V34_03965|RLP83965	sp|A4ST17	ho|UniRef50_A0A098QZ41	dash|A0A088CB91
ref|EDX24144	D9V34_04270|RLP84022	sp|A7MFY0	ho|UniRef50_A0A098RYH5	dash|A0A0B5KBT7
ref|EDX26411	D9V34_06615|RLP82916	sp|A7MST3	ho|UniRef50_A0A0A1WG21	dash|A0A0B5KGV8
ref|K4HQE7	D9V34_06715|RLP83355	sp|B3A0N5	ho|UniRef50_A0A0A9YZC7	dash|A0A0B5WSQ6
ref|P00780	D9V34_08660|RLP83286	sp|B4TD53	ho|UniRef50_A0A0C5C405	dash|A0A0H2WW38
ref|P0AEE3	D9V34_09435|RLP82036	sp|B5BLW5	ho|UniRef50_A0A0E9NH89	dash|A0A0M3KKZ9
ref|P23953	D9V34_09665|RLP82078	sp|B5EXN3	ho|UniRef50_A0A0F2P3A7	dash|A0A0M4AV81
ref|P28296	D9V34_09985|RLP82138	sp|B7M3W8	ho|UniRef50_A0A0F7VQB7	dash|A0A0M5I297
ref|P29118	D9V34_17035|RLP78909	sp|B7VHH1	ho|UniRef50_A0A0F9GVU5	dash|A0A0R5NGA4
ref|P29142	D9V34_17135|RLP78932	sp|B8M2K0	ho|UniRef50_A0A0F9M9H4	dash|A0A0X1KHD1
ref|P29600	D9V34_17220|RLP78818	sp|C0NM08	ho|UniRef50_A0A0G0P7Y4	dash|A0A182DWC8
ref|P35211		sp|C4LA13	ho|UniRef50_A0A0J0YNC1	dash|A0A2H4A2Y5
ref|Q03420		sp|M2YJ38	ho|UniRef50_A0A0K1Q5E7	dash|A0A2R2JFW8
ref|Q7SIG1		sp|O05213	ho|UniRef50_A0A0M0KEH7	dash|A0A3B6UEW8
ref|Q86WA6		sp|O06734	ho|UniRef50_A0A0M9VJU7	dash|A0QVS8
ref|Q9WXD6		sp|O07427	ho|UniRef50_A0A0N0MB81	dash|A3MVR4
ref|WP_037795014		sp|O22609	ho|UniRef50_A0A0N7JF09	dash|A6G7B1
		sp|O31158	ho|UniRef50_A0A0P8AZ07	dash|A9ST85
		sp|O31168	ho|UniRef50_A0A0Q8MXH5	dash|B1YPY7
		sp|O31431	ho|UniRef50_A0A0R2BEA1	dash|B4E794
		sp|O53695	ho|UniRef50_A0A0R2QZ53	dash|B6I985
		sp|P00781	ho|UniRef50_A0A0R3UCH6	dash|D0AB41
		sp|P00811	ho|UniRef50_A0A0S6XIE4	dash|D2J2T6
		sp|P04189	ho|UniRef50_A0A0U2ZP60	dash|E1VFE0
		sp|P06196	ho|UniRef50_A0A0V1CL24	dash|E6Y8B9
		sp|P06276	ho|UniRef50_A0A135L162	dash|E7DJY5
		sp|P07024	ho|UniRef50_A0A142YCE6	dash|G2PVG6
		sp|P07383	ho|UniRef50_A0A143HHH4	dash|G3CR02
		sp|P07518	ho|UniRef50_A0A160IQG5	dash|G4I2J6
		sp|P0A3G2	ho|UniRef50_A0A172ZFQ3	dash|K0ACL0
		sp|P0C0V0	ho|UniRef50_A0A177CBT6	dash|O06441
		sp|P11018	ho|UniRef50_A0A1B0G6N0	dash|O07015
		sp|P12337	ho|UniRef50_A0A1B3NW56	dash|O28735
		sp|P15292	ho|UniRef50_A0A1C2FY22	dash|O82777
		sp|P15293	ho|UniRef50_A0A1C7NZQ0	dash|P00730
		sp|P15304	ho|UniRef50_A0A1D2MNH2	dash|P00782
		sp|P15926	ho|UniRef50_A0A1E3T6L8	dash|P04058
		sp|P16271	ho|UniRef50_A0A1E7I9K2	dash|P06873

		sp|P16396	ho|UniRef50_A0A1E7Z597	dash|P07140
		sp|P16854	ho|UniRef50_A0A1F3BHH7	dash|P08594
		sp|P18143	ho|UniRef50_A0A1F3SQ44	dash|P09954
		sp|P19076	ho|UniRef50_A0A1F4U3X1	dash|P09955
		sp|P21836	ho|UniRef50_A0A1F5ZS68	dash|P13001
		sp|P22303	ho|UniRef50_A0A1F6GV27	dash|P15085
		sp|P22848	ho|UniRef50_A0A1F6IGL6	dash|P15086
		sp|P22862	ho|UniRef50_A0A1F8MSZ9	dash|P22643
		sp|P23141	ho|UniRef50_A0A1F8PCE2	dash|P23872
		sp|P24484	ho|UniRef50_A0A1F8WN84	dash|P28321
		sp|P25026	ho|UniRef50_A0A1G1ECJ6	dash|P33912
		sp|P26982	ho|UniRef50_A0A1G7BJP1	dash|P34913
		sp|P27693	ho|UniRef50_A0A1G9K999	dash|P34914
		sp|P29068	ho|UniRef50_A0A1H3FB32	dash|P59336
		sp|P29139	ho|UniRef50_A0A1H9GZP2	dash|P71702
		sp|P29141	ho|UniRef50_A0A1I2F7C9	dash|P77044
		sp|P29599	ho|UniRef50_A0A1J5DY67	dash|P96965
		sp|P29715	ho|UniRef50_A0A1J5IC86	dash|P9WNH5
		sp|P34211	ho|UniRef50_A0A1P8B4Y8	dash|Q01471
		sp|P35835	ho|UniRef50_A0A1P8MX87	dash|Q07596
		sp|P37967	ho|UniRef50_A0A1Q4C365	dash|Q0ZPV7
		sp|P39041	ho|UniRef50_A0A1Q5NZC9	dash|Q13KT2
		sp|P39099	ho|UniRef50_A0A1Q5PMI7	dash|Q1GDP2
		sp|P39668	ho|UniRef50_A0A1Q7GZH4	dash|Q1JU72
		sp|P41363	ho|UniRef50_A0A1R0GP43	dash|Q1QBB9
		sp|P44569	ho|UniRef50_A0A1T3P3M0	dash|Q1QEU6
		sp|P44764	ho|UniRef50_A0A1U7XNF8	dash|Q25252
		sp|P45129	ho|UniRef50_A0A1U8FYH3	dash|Q29550
		sp|P47229	ho|UniRef50_A0A1V4HAX8	dash|Q2KIG3
		sp|P48052	ho|UniRef50_A0A1V4THE6	dash|Q2PCE5
		sp|P49323	ho|UniRef50_A0A1V5AUG4	dash|Q3HV58
		sp|P58099	ho|UniRef50_A0A1V5UPG3	dash|Q5EPH2
		sp|P58502	ho|UniRef50_A0A1V5US96	dash|Q62765
		sp|P65822	ho|UniRef50_A0A1V5WH94	dash|Q68GV9
		sp|P72780	ho|UniRef50_A0A1V6FVU8	dash|Q6DND9
		sp|P75736	ho|UniRef50_A0A1V8NCN8	dash|Q6L545
		sp|P77619	ho|UniRef50_A0A1W1W321	dash|Q6N9M9
		sp|P86325	ho|UniRef50_A0A1W6ZS38	dash|Q6NAM1
		sp|P9WHR3	ho|UniRef50_A0A1W9MJM3	dash|Q75WN8
		sp|P9WK86	ho|UniRef50_A0A1W9P3Z0	dash|Q83PW0
		sp|P9WNH1	ho|UniRef50_A0A1X7CBC6	dash|Q868D6
		sp|Q01470	ho|UniRef50_A0A1Y1MNK7	dash|Q8IVL8
		sp|Q13QH4	ho|UniRef50_A0A1Y5GTM5	dash|Q8N0W4
		sp|Q1PET6	ho|UniRef50_A0A1Z9FHT4	dash|Q8N2Q7
		sp|Q2VLB9	ho|UniRef50_A0A210QBV2	dash|Q8Z0Q1
		sp|Q325C0	ho|UniRef50_A0A238V5U7	dash|Q8ZLI9

		sp|Q39547	ho|UniRef50_A0A239FRD0	dash|Q8ZRA1
		sp|Q45670	ho|UniRef50_A0A256ZZ96	dash|Q8ZRI7
		sp|Q47M62	ho|UniRef50_A0A257G8Q6	dash|Q92035
		sp|Q48EU9	ho|UniRef50_A0A285CK03	dash|Q96IY4
		sp|Q49418	ho|UniRef50_A0A2A5CHD3	dash|Q97KV0
		sp|Q49KF8	ho|UniRef50_A0A2A5W4W2	dash|Q988D4
		sp|Q49WF1	ho|UniRef50_A0A2C9K6T0	dash|Q99685
		sp|Q504N0	ho|UniRef50_A0A2D5G357	dash|Q99K10
		sp|Q52894	ho|UniRef50_A0A2D5K6B1	dash|Q9A919
		sp|Q54410	ho|UniRef50_A0A2D7I4V4	dash|Q9GPG0
		sp|Q55921	ho|UniRef50_A0A2D8GMX7	dash|Q9HZF5
		sp|Q59695	ho|UniRef50_A0A2D8LF58	dash|Q9I638
		sp|Q5QZC0	ho|UniRef50_A0A2D9HXJ3	dash|Q9MAA7
		sp|Q5UQ83	ho|UniRef50_A0A2D9XD92	dash|Q9SQR3
		sp|Q64176	ho|UniRef50_A0A2E2JE49	dash|Q9UI42
		sp|Q6AW46	ho|UniRef50_A0A2E3A4A0	dash|Q9VMC9
		sp|Q6GMI0	ho|UniRef50_A0A2E3IN72	dash|U3KRE9
		sp|Q6NT32	ho|UniRef50_A0A2E4HPX3	dash|V5J5W4
		sp|Q82SL8	ho|UniRef50_A0A2E5KVG2	
		sp|Q869C3	ho|UniRef50_A0A2E7HY55	
		sp|Q86GC8	ho|UniRef50_A0A2E7IJW4	
		sp|Q89AP5	ho|UniRef50_A0A2E7USP9	
		sp|Q8DFG4	ho|UniRef50_A0A2E7W6A1	
		sp|Q8VCT4	ho|UniRef50_A0A2E8HZB5	
		sp|Q8VYP9	ho|UniRef50_A0A2E9JDH3	
		sp|Q8XBJ0	ho|UniRef50_A0A2E9Y9F7	
		sp|Q8YAJ5	ho|UniRef50_A0A2G4FC93	
		sp|Q8ZN80	ho|UniRef50_A0A2H0PK44	
		sp|Q93LQ6	ho|UniRef50_A0A2H5N9K6	
		sp|Q94AS5	ho|UniRef50_A0A2H5Q0M9	
		sp|Q99405	ho|UniRef50_A0A2H6HHT3	
		sp|Q9AQM4	ho|UniRef50_A0A2J6KIK5	
		sp|Q9FCD7	ho|UniRef50_A0A2J6NX09	
		sp|Q9KX40	ho|UniRef50_A0A2K1JQQ1	
		sp|Q9LFR7	ho|UniRef50_A0A2K8N3K8	
		sp|Q9LNU1	ho|UniRef50_A0A2M7ALU9	
		sp|Q9LVJ1	ho|UniRef50_A0A2M7WGB9	
		sp|Q9SZV5	ho|UniRef50_A0A2N1UUE4	
		sp|Q9US38	ho|UniRef50_A0A2N3F424	
		tr|A0A023T3X2	ho|UniRef50_A0A2N5K364	
		tr|A0A0H2VD83	ho|UniRef50_A0A2N6C048	
		tr|A0A0H4B872	ho|UniRef50_A0A2N9LX72	
		tr|A0A0S4G6Z4	ho|UniRef50_A0A2R6JQ66	
		tr|A0A143I3F8	ho|UniRef50_A0A2S6MWP1	
		tr|A0A2B6CI11	ho|UniRef50_A0A2S6TVK5	
		tr|A0A384LFU1	ho|UniRef50_A0A2S7K4A3	

		tr|A0QQ47	ho|UniRef50_A0A2T1A528	
		tr|A3JB27	ho|UniRef50_A0A2T4VEN3	
		tr|A5VAT9	ho|UniRef50_A0A2U2S5K7	
		tr|A7TUE6	ho|UniRef50_A0A2U3LEU5	
		tr|B0V9K7	ho|UniRef50_A0A2U3LGC0	
		tr|B2CZF4	ho|UniRef50_A0A2V9HUZ0	
		tr|B4EA96	ho|UniRef50_A0A2V9MRB6	
		tr|B6ETQ5	ho|UniRef50_A0A2W4J4K4	
		tr|D3AU79	ho|UniRef50_A0A2Z5JZ48	
		tr|D5GA36	ho|UniRef50_A0A316TR45	
		tr|F0NDQ1	ho|UniRef50_A0A317E510	
		tr|G2J5T2	ho|UniRef50_A0A317I779	
		tr|G9BEX6	ho|UniRef50_A0A321L860	
		tr|I6YRG4	ho|UniRef50_A0A321LJ07	
		tr|K0C6T6	ho|UniRef50_A0A323VIZ6	
		tr|L7PYQ2	ho|UniRef50_A0A328A5L7	
		tr|O28558	ho|UniRef50_A0A350G0J5	
		tr|O53896	ho|UniRef50_A0A350UVM0	
		tr|P94288	ho|UniRef50_A0A354ZCR1	
		tr|Q23734	ho|UniRef50_A0A355UVA6	
		tr|Q3HWU8	ho|UniRef50_A0A366LPM1	
		tr|Q5G935	ho|UniRef50_A0A370TLK4	
		tr|Q5NU42	ho|UniRef50_A0A378K5K0	
		tr|Q5SIP1	ho|UniRef50_A0A378LID2	
		tr|Q65DC7	ho|UniRef50_A0A398BY28	
		tr|Q84II3	ho|UniRef50_A0A399WUU0	
		tr|Q8GCC7	ho|UniRef50_A0A3A0B3V9	
		tr|Q8GJP7	ho|UniRef50_A0A3A0DZ37	
		tr|Q8XDQ2	ho|UniRef50_A0A3A0ENX5	
		tr|Q976W8	ho|UniRef50_A0A3A0G9X6	
		tr|Q9KJG6	ho|UniRef50_A0A3A0GEL6	
		tr|Q9V2D6	ho|UniRef50_A0A3A1WUR4	
			ho|UniRef50_A0A3A4BF78	
			ho|UniRef50_A0A3A4ZA56	
			ho|UniRef50_A0A3A5FW30	
			ho|UniRef50_A0A3B1CE70	
			ho|UniRef50_A0A3B4U9R9	
			ho|UniRef50_A0A3B8Z128	
			ho|UniRef50_A0A3B9MRG0	
			ho|UniRef50_A0A3C0ICY5	
			ho|UniRef50_A0A3C1DI76	
			ho|UniRef50_A0A3D3YV81	
			ho|UniRef50_A0A3D4UE01	
			ho|UniRef50_A0A3D4XRD1	
			ho|UniRef50_A0A3D8LE24	
			ho|UniRef50_A0A3E1YF31	

			ho|UniRef50_A0A3M1QZM8	
			ho|UniRef50_A0A3M2BER7	
			ho|UniRef50_A0A3M7IFE9	
			ho|UniRef50_A0A3N4R794	
			ho|UniRef50_A0A3N5FHY3	
			ho|UniRef50_A0A3N5MEB1	
			ho|UniRef50_A0A3N5VWC2	
			ho|UniRef50_A0A3Q9HQI4	
			ho|UniRef50_A0A3R8R702	
			ho|UniRef50_A0A402FZA2	
			ho|UniRef50_A0A418KYC8	
			ho|UniRef50_A0A419E5J2	
			ho|UniRef50_A0A427ZJY1	
			ho|UniRef50_A0A431P865	
			ho|UniRef50_A0A443SD88	
			ho|UniRef50_A0A448KB33	
			ho|UniRef50_A0A450PE40	
			ho|UniRef50_A0A485E972	
			ho|UniRef50_A0A494RXS1	
			ho|UniRef50_A0A497ML03	
			ho|UniRef50_A0A4D6MMC7	
			ho|UniRef50_A0A4D8Y0M1	
			ho|UniRef50_A0A4P6UVI9	
			ho|UniRef50_A0A4Q1TAX0	
			ho|UniRef50_A0A4Q2B2P8	
			ho|UniRef50_A0A4Q3PMA0	
			ho|UniRef50_A0A4Q3UGV6	
			ho|UniRef50_A0A4Q7KQE7	
			ho|UniRef50_A0A4R2EVY3	
			ho|UniRef50_A0A4R3KRL5	
			ho|UniRef50_A0A4R4Q9Z4	
			ho|UniRef50_A0A4R4XEP2	
			ho|UniRef50_A0A4R6VQT9	
			ho|UniRef50_A0A4R7LJF7	
			ho|UniRef50_A0A4R7UX36	
			ho|UniRef50_A0A4S2J9X3	
			ho|UniRef50_A0A4U1JBC8	
			ho|UniRef50_A0A4V2BGD5	
			ho|UniRef50_A0A4W4FFC9	
			ho|UniRef50_A0A511TBM0	
			ho|UniRef50_A0A517R6S6	
			ho|UniRef50_A0A521YRI1	
			ho|UniRef50_A0A532TPL5	
			ho|UniRef50_A0A533VAS6	
			ho|UniRef50_A0A536ABA3	
			ho|UniRef50_A0A536FRI2	

			ho|UniRef50_A0A536N0V4	
			ho|UniRef50_A0A537S2L5	
			ho|UniRef50_A0A540VLC7	
			ho|UniRef50_A0A542E5G2	
			ho|UniRef50_A0A545T7R5	
			ho|UniRef50_A0A561QEM2	
			ho|UniRef50_A0A5A5TG70	
			ho|UniRef50_A0A5B8RKU7	
			ho|UniRef50_A0A5B8YDW8	
			ho|UniRef50_A0A5C4VE90	
			ho|UniRef50_A0A5C8ZQJ8	
			ho|UniRef50_A0A5C9F1E3	
			ho|UniRef50_A0A5E4KCQ6	
			ho|UniRef50_A0A5E4MEJ6	
			ho|UniRef50_A0A5J4E3Q3	
			ho|UniRef50_A0A5J5GIQ8	
			ho|UniRef50_A0A5K1GHS1	
			ho|UniRef50_A0YQR5	
			ho|UniRef50_A1ASS9	
			ho|UniRef50_A5CU31	
			ho|UniRef50_A6F4A4	
			ho|UniRef50_A6TU64	
			ho|UniRef50_A8TT96	
			ho|UniRef50_B0T7I5	
			ho|UniRef50_B2IZH4	
			ho|UniRef50_B3PI89	
			ho|UniRef50_B3S320	
			ho|UniRef50_B4D3Q8	
			ho|UniRef50_B4DCL8	
			ho|UniRef50_B7P7C8	
			ho|UniRef50_C3Y0X9	
			ho|UniRef50_C3ZD77	
			ho|UniRef50_D3Q7M8	
			ho|UniRef50_D5MN57	
			ho|UniRef50_D6TJ02	
			ho|UniRef50_D9VEE5	
			ho|UniRef50_E0UID1	
			ho|UniRef50_E1SM52	
			ho|UniRef50_E1VSI5	
			ho|UniRef50_F3L1Y6	
			ho|UniRef50_F4G0R8	
			ho|UniRef50_F4KSN2	
			ho|UniRef50_F4LVJ6	
			ho|UniRef50_F5SF31	
			ho|UniRef50_F8DZ48	
			ho|UniRef50_F8GUP4	

			ho|UniRef50_F9F9A3	
			ho|UniRef50_G2LIP5	
			ho|UniRef50_G8R595	
			ho|UniRef50_H1G3E2	
			ho|UniRef50_H5WNX9	
			ho|UniRef50_J3KJ43	
			ho|UniRef50_K9GVG0	
			ho|UniRef50_L0IE41	
			ho|UniRef50_M0AXM2	
			ho|UniRef50_M0JZ16	
			ho|UniRef50_M0S199	
			ho|UniRef50_M7NIA0	
			ho|UniRef50_N0E1W7	
			ho|UniRef50_O34592	
			ho|UniRef50_P15088	
			ho|UniRef50_Q04QY1	
			ho|UniRef50_Q2QSI6	
			ho|UniRef50_Q39NZ2	
			ho|UniRef50_Q39WE2	
			ho|UniRef50_Q45669	
			ho|UniRef50_Q62889-3	
			ho|UniRef50_Q70GK8	
			ho|UniRef50_Q7RTL7	
			ho|UniRef50_Q83ZF0	
			ho|UniRef50_Q8MZM0	
			ho|UniRef50_Q8RVA0	
			ho|UniRef50_Q98RH3	
			ho|UniRef50_R1GC70	
			ho|UniRef50_R6HZ86	
			ho|UniRef50_R6LMD1	
			ho|UniRef50_S5TFX7	
			ho|UniRef50_UPI00039E34AD	
			ho|UniRef50_UPI0003F0C383	
			ho|UniRef50_UPI00041865CA	
			ho|UniRef50_UPI00041BF1ED	
			ho|UniRef50_UPI0004296061	
			ho|UniRef50_UPI00047AFCDE	
			ho|UniRef50_UPI00052795A2	
			ho|UniRef50_UPI0005AAAAA6	
			ho|UniRef50_UPI00068C9C1A	
			ho|UniRef50_UPI0006C8F8B9	
			ho|UniRef50_UPI0007077F1F	
			ho|UniRef50_UPI0007168208	
			ho|UniRef50_UPI0008345B4B	
			ho|UniRef50_UPI0009019D3E	
			ho|UniRef50_UPI0009ACB5FC	

			ho|UniRef50_UPI0009E09AF7	
			ho|UniRef50_UPI0009E45E80	
			ho|UniRef50_UPI0009E99AF4	
			ho|UniRef50_UPI00101F6243	
			ho|UniRef50_UPI001115DF2E	
			ho|UniRef50_UPI0011CBC7C4	
			ho|UniRef50_V7HYK8	
			ho|UniRef50_W2UDL9	
			ho|UniRef50_W5XQ41	
			ho|UniRef50_W7UH79	
			ho|UniRef50_W8EVD3	
			ho|UniRef50_X5M993	

References in *SI Appendix*

1. E. H. Harris, *The* Chlamydomonas *sourcebook: a comprehensive guide to biology and laboratory use* (Academic Press Inc, 1989).

2. A. Ramette, *et al.*, *Pseudomonas protegens* sp. nov., widespread plant-protecting bacteria producing the biocontrol compounds 2,4-diacetylphloroglucinol and pyoluteorin. *Syst. Appl. Microbiol.* **34**, 180–188 (2011).

3. T. Tsukamoto, M. Takeuchi, O. Shida, H. Murata, A. Shirata, Proposal of *Mycetocola* gen. nov. in the family *Microbacteriaceae* and three new species, *Mycetocola saprophilus* sp. nov., *Mycetocola tolaasinivorans* sp. nov. and *Mycetocola lacteus* sp. nov., isolated from cultivated mushroom, *Pleurotus ostreatus*. *Int. J. Syst. Evol. Microbiol.* **51**, 937–944 (2001).

4. S. V. Pollock, "Quick and easy genomic DNA prep for PCR analysis" (Laudon M. St. Paul, Minnesota: Chlamydomonas Resource Center., 2003).

5. M. Araya, S. García, J. Rengel, S. Pizarro, G. Álvarez, Determination of free and protein amino acid content in microalgae by HPLC-DAD with pre-column derivatization and pressure hydrolysis. *Mar. Chem.* **234**, 103999 (2021).

6. R. H. Girdwood, The microbiological assay of vitamins and amino acids. *J. Med. Lab. Technol.* **20**, 26–33 (1963).

7. J. H. Miller, *Experiments in molecular genetics* (Cold Spring Harbor Laboratory, 1972).

8. M. T. Lin, *et al.*, "*Escherichia coli* auxotroph host strains for amino acid-selective isotope labeling of recombinant proteins" in *Methods in Enzymology*, (Elsevier, 2015), pp. 45–66.

9. A. Glinko, M. J. Bozym, M. L. Owens, K. M. Usher, R. E. Majors, Reversed-phase HPLC separation of water-soluble vitamins on agilent ZORBAX eclipse plus columns. *Methods Agil. Technol.*, 1–8 (2008).

10. L. Szydlowski, *et al.*, Evolutionary dynamics of microbial communities in bioelectrochemical systems (2019) https:/doi.org/10.1101/725580 (December 19, 2023).

11. P. Durán, *et al.*, Shared features and reciprocal complementation of the *Chlamydomonas* and *Arabidopsis* microbiota. *Nat. Commun.* **13**, 406 (2022).

12. S. Pfeiffer, *et al.*, Rhizosphere microbiomes of potato cultivated in the High Andes show stable and dynamic core microbiomes with different responses to plant development. *FEMS Microbiol. Ecol.* **93**, fiw242 (2017).

13. P. Palladino, D. A. Stetsenko, New TFA-free cleavage and final deprotection in fmoc solid-phase peptide synthesis: Dilute HCl in fluoro alcohol. *Org. Lett.* **14**, 6346–6349 (2012).

14. M. De Vleeschouwer, *et al.*, Identification of the molecular determinants involved in antimicrobial activity of pseudodesmin A, a cyclic lipopeptide from the viscosin group. *Front. Microbiol.* **11**, 646 (2020).

15. G. R. Fulmer, *et al.*, NMR chemical shifts of trace impurities: Common laboratory solvents, organics, and gases in deuterated solvents relevant to the organometallic chemist. *Organometallics* **29**, 2176–2179 (2010).

16. Y. Bando, *et al.*, Total synthesis and structure correction of the cyclic lipodepsipeptide Orfamide A. *Chem. – Eur. J.* **28**, e202104417 (2022).

17. P. Aiyar, *et al.*, Antagonistic bacteria disrupt calcium homeostasis and immobilize algal cells. *Nat. Commun.* **8**, 1756 (2017).

18. M. D. Fricker, *et al.*, "Fluorescence and luminescence techniques to probe ion activities inliving plant cells" in *Fluorescent and luminescent probes for biological activity*, (Elsevier, 1999), pp. 569–596.
